# Exploring plastic biofilm formation and *Escherichia coli* colonisation in marine environments

**DOI:** 10.1111/1758-2229.13308

**Published:** 2024-06-24

**Authors:** Elisenda Ballesté, Hongxia Liang, Laura Migliorato, Laura Sala‐Comorera, Javier Méndez, Cristina Garcia‐Aljaro

**Affiliations:** ^1^ Departament de Genètica, Microbiologia i Estadística, Facultat de Biologia Universitat de Barcelona Barcelona Spain; ^2^ College of Resources and Environment University of Chinese Academy of Sciences Beijing China; ^3^ Beijing Municipal Ecological and Environmental Monitoring Center Beijing China

## Abstract

Microorganisms, including potential pathogens, can colonise plastic surfaces in aquatic environments. This study investigates the colonisation of plastic pellets by *Escherichia coli* (*E. coli*) as a proxy for faecal pathogens in aquatic environments. Plastic pellets from a polluted beach were placed in seawater aquaria spiked with *E. coli*. Diverse bacteria, primarily from the Proteobacteria phylum, rapidly colonised the pellets within 24 h, with notable species known for plastic or hydrocarbon degradation. Over 26 days, biofilms formed on the plastic surfaces, reaching bacterial populations of up to 6.8·10^5^ gene copies (gc) of the 16S rRNA mm^−2^. *E. coli*, was detected in the pellets for up to 7 days using culture methods, exhibiting varying attachment densities regardless of source or environmental factors. The study highlights plastic biofilms as reservoirs for *E. coli*, contributing to the survival and persistence of faecal bacteria in aquatic systems. These findings deepen our understanding of the risks associated with plastic pollution in marine settings, offering insights into the behaviour of faecal indicators and their implications for water quality assessments, while providing valuable information on potential pathogen dissemination within plastic‐associated microbial communities.

## Graphical abstract

This study examines how *Escherichia coli* and other bacteria colonise plastic pellets in seawater. Within 24 h, diverse bacteria colonised the pellets, forming a mature biofilm from day 12. *E. coli* was detected for up to 7 days, showing varying attachment densities. Plastisphere act as reservoir for *E. coli*, influencing its survival and dissemination in aquatic systems, indicating implications for water quality assessment and pathogen dissemination in plastic‐associated microbial communities.

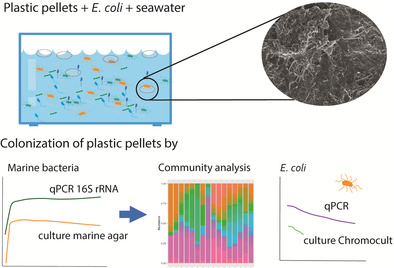

## INTRODUCTION

Plastic debris has become ubiquitous in aquatic ecosystems, spanning from heavily impacted areas to the most pristine environments (González‐Pleiter et al., 2020; McCormick et al., 2014). The improper disposal of plastic, estimated at 58% of the total plastic disposal, accumulates in landfills or in natural environments, where it can persist from 58 years (PET bottles) to 1200 years (HDPE pipes) (Chamas et al., 2020; Geyer et al., 2017). The most ambitious scenario suggests that between 20 and 53 Mt of plastic waste could enter aquatic ecosystems by 2030 (Borrelle et al., 2020). Once in seawater, plastic surfaces rapidly adsorb organic and inorganic matter, generating a nutrient‐rich layer within hours and facilitating the attachment of bacteria in <24 h (Qian et al., 2022). The term plastisphere defines the microbial community thriving on plastics (Zettler et al., 2013) and exhibits densities ranging from 1.5 × 10^5^ to 8.7 × 10^6^ 16S rRNA gene copies (gc) mm^−2^ covering the floating surfaces (Liang et al., 2023).

Biofilms function as a complex self‐sustaining ecosystem with a cooperation and competition relationship between microorganisms (Nadell et al., 2016). The biofilm provides protection against grazing, desiccation, solar radiation, and exposure to antibiotics. Bacteria within the biofilm can readily absorb nutrients produced as wastes by neighbouring bacteria, while horizontal gene transfer enhances the dissemination of novel functions within the plastisphere (Qian et al., 2022). Considering that any substrate immersed in seawater is rapidly colonised by bacteria, the large amount of already floating plastics represents a huge new potential artificial substrate drifting through the waterbodies carrying microorganisms lasting longer than natural floating substrates.

Bacteria identified on plastic biofilms include hydrocarbon degraders like *Pseudoalteromonas* spp. or *Phormidium* spp. but also members of potential pathogenic clades such as *Campylobacteraceae*, *Enterobacteriaceae, Pseudomonas*, or *Vibrio* (McCormick et al., 2014; Wu et al., 2019; Zettler et al., 2013). These potential pathogens are normally identified after sequencing a 300 bp fragment of the 16S rRNA gene that limits their classification at the genus level and provides limited information about viability or pathogenicity.

Due to the challenges in directly monitoring pathogens in water samples, bacterial faecal indicators such as *E. coli* and Enterococci are used as proxies and are included in water quality regulations like the European bathing directive (European Commission, 2006). A recent study has detected both indicators on plastics collected from coastal waters with human impact, detecting higher densities of *E. coli* per item compared to the surrounding water (Liang et al., 2023). Moreover, these faecal indicators have demonstrated the capacity to adhere to plastic biofilms (Metcalf et al., 2023) and they have also been detected on plastic debris found on beaches (Hernández‐Sánchez et al., 2023; Rodrigues et al., 2019). The presence of *E. coli* attached to plastics raises concerns about the potential presence of faecal pathogens. Incubation experiments in sewage have revealed the colonisation of plastics by potential pathogens such as *Pseudomonas*, *Arcobacter*, and *Mycobacterium* and by bacteria carrying antibiotic resistance genes *sul*I and *tet*M (Martínez‐Campos et al., 2021). However, although another study detected *E. coli* producing extended‐spectrum beta‐lactamase in water, it was not detected in plastic polymers incubated in the same water (Song et al., 2020). Therefore, it seems that *E. coli* can attach to plastic biofilms, but there may be differences in colonisation patterns due to environmental conditions and the detection techniques used.

To address these knowledge gaps, this study aimed to investigate the ability of *E. coli*, serving as a proxy for bacterial faecal pathogens, to colonise plastics in seawater. Additionally, we sought to evaluate the persistence and stability of *E. coli* within the evolving biofilm, which becomes enriched by marine bacteria over time. Four independent microcosm experiments were conducted, involving the incubation of environmental plastic pellets with different strains of *E. coli* in seawater. The abundance of *E. coli*, as well as the composition and abundance of marine bacteria, were monitored using a combination of culture‐based and molecular techniques.

By elucidating the colonisation dynamics of *E. coli* on plastic surfaces, this study contributes to our understanding of the risks associated with plastic pollution in marine ecosystems. The findings will provide valuable insights into the behaviour of *E. coli* as a faecal indicator bacterium in biofilms, aiding in the development of models for assessing the risk associated with plastic biofilms and informing effective mitigation strategies.

## EXPERIMENTAL PROCEDURES

### Microcosms and sampling process

We performed four independent microcosms (MC1–4). Each microcosm was set in one aquarium which included (i) 170 plastic pellets collected from a beach (ii) seawater provided by the ‘Centres Científics i Tecnològics’ from Universitat de Barcelona (CCiTUB) and (iii) a mix of environmental strains of *E. coli* (Figure S1). The microcosms were performed at different time periods, so the seawater was different (Table S1).

We used environmental plastics since they have gone through a natural process of weathering, so they allow to reproduce a real environmental colonisation; thus, a mix of plastic pellets was collected from the sand of a beach of la Pineda (Tarragona, Spain). Since plastic pellets were collected from the environment, they presented variability in their composition, size, and degree of abrasion. They had a mean diameter of 4.6 mm (3.9–5.6 mm) and an estimated mean surface sphere area of 77 mm^2^ (58–107 mm^2^). Analyses performed using a Perkin Elmer Frontier FT‐IR Spectrometer Fourier transform infrared spectrophotometry in CCiTUB identified them as polyethylene (PE) and polypropylene (PP) (the 80% and 20%, respectively). To reduce the variability between pellets, for each sampling and each analysis, we pooled a given number of randomly selected pellets, and we mixed them (Schneider et al., 2012). Before use, the plastic pellets were disinfected with H_2_O_2_ 7.5% for 6 h and washed with sterile seawater. Disinfected pellets were used as control for the different analyses.

For MC1 and MC2, we used a mix of three *E. coli* strains isolated from sewage and for MC3 and MC4, and we used a mix of three *E. coli* strains isolated from plastic samples collected in coastal waters from a previous study (Liang et al., 2023). The strains were isolated from sewage or plastic pellets using the selective and differential media Chromocult and identified using API20E strips (bioMerieux, Paris, France) and by Sanger sequencing of the 16S rRNA gene with universals primers 27f and 1492r (Table S2). The selected *E. coli* strains showed >98% of identity with *E. coli*.

The aquariums were filled with 20 L of seawater from the wet lab facilities and the absence of *E. coli* was verified by culture. We spiked the aquaria with 1.1 × 10^4^–6.4 × 10^4^ cfu of the mixed overnight grown *E. coli* strains per ml^−1^, and we added 170 disinfected plastic pellets. Aquariums were kept in stable conditions in the wet lab of CCiTUB. Water was kept at 20°C (±2°C), recirculated using a pump, oxygenated using an aerator, and kept with alternation between light and dark every 12 h. A total of 20 plastic pellets were collected randomly at days 1, 2, 5, 7, 12, 19, and 26 to characterise and enumerate bacteria from the plastisphere and were used for the different analysis. Meanwhile, water was collected at the beginning (day 0) and at the end of the experiment (day 26) to monitor potential changes. The physicochemical characteristics were measured including pH, dissolved oxygen, salinity using a portable multiparameter probe HI‐98194 (Hanna Instruments), and total organic carbon, inorganic carbon, total carbon, and total nitrogen were measured in CCiTUB with a TOC analyser multi N/C 3100 (Jena).

### *Enumeration of* E. coli *and marine bacteria by culture media*

*E. coli* was measured using Chromocult® Coliform Agar (Merck, Darmstadt, Germany) including the *E. coli*/Coliform selective supplement (Merk) (2.5 mg of cefsulodine and vancomycin per 500 mL of Chromocult) and incubated at 37°C for 24 h (ISO, 2000a). The abundance of heterotrophic marine bacteria was quantified using Marine Agar 2216 (Difco, Madrid, Spain) after incubation for 48 h at 20°C. To evaluate culturable bacteria, we pooled five plastic pellets for each time and for each microcosm. Bacteria on the plastic biofilm were detached in sterile seawater after 1 min of sonication in an ultrasound bath, obtaining a bacterial suspension. The water samples and the biofilm bacterial suspension were diluted using sterile seawater if the bacterial concentration was too high. Alternatively, if the bacterial concentration was too low, they were concentrated by filtration through a 0.45 μm pore size filter (EZ‐PAK, Millipore, Darmstadt, Germany) before seeding in the required media. Results were expressed as cfu mm^−2^ in plastic pellets or cfu mL^−1^ in water.

### Enumeration of microorganisms by molecular methods

#### DNA extraction

For each time and microcosms, we pooled five plastic pellets to avoid differences between plastic pellets and to extract enough DNA. Besides, we extracted the DNA from 0.5 L of seawater concentrated by filtration with a 0.22 μm pore size cellulose ester membrane (SO‐PAK, Millipore, Darmstadt, Germany) from the beginning (T0) and from the end of the experiment (T26) to evaluate changes in the water microbial community. The DNeasy PowerBiofilm Extraction Kit (Qiagen, Hilden, Germany) was used following the manufacturer's instructions, and the DNA extracted was eluted to a final volume of 100 μL. DNA extraction controls including disinfected pellets and filtered seawater were run together with the samples.

#### *Quantification of* E. coli *and total 16S rRNA gene*

Total *E. coli* was quantified by targeting a fragment of the 16S rRNA gene by qPCR, as previously described (Huijsdens et al., 2002). The total 16S rRNA gene was quantified using the primers 341F and 534R (Muyzer et al., 1995, 1996).

Amplification of *E. coli* a was performed using TaqMan Environmental Master Mix 2.0 (Applied Biosystems, Foster City, CA, USA) by a StepOne Real‐Time PCR System (Applied Biosystems, Foster City, CA, USA). Each mixture, with a final volume of 20 μL, was composed of 10 μL of TaqMan Environmental Master Mix 2.0 (Applied Biosystems), 300 nM of the primers and 100 nM of the probe (Table S2), 5 μL of the DNA template, and nuclease‐free water to reach the final volume. Amplifications were done under the following conditions: 10 min of an initial denaturation at 95°C, followed by 40 cycles of 15 s of denaturation at 95°C and 1 min of annealing and extension at 60°C.

PCR amplification of the 16S rRNA gene was carried out in a 20 μL reaction mixture with 10 μL of PowerUp SYBR Green Master Mix (Thermo Fisher Scientific, Waltham, MA, USA), 1000 nM of the primers 341F and 534R (Table S2), 1 μL of the DNA template, and nuclease‐free water to reach the final volume. The PCR program was initiated at 95°C for 10 min, followed by 40 cycles of denaturation at 95°C for 15 s, annealing at 60°C for 15 s, and extension at 60°C for 1 min.

All samples, negative controls, and extraction and filtration blanks were run in duplicate. Molecular results of microorganisms from plastics and water were expressed as gc mm^−2^ and gc ml^−1^, respectively. Five points of the standard curves were included in duplicate for each run and were generated from different 10‐fold serial dilutions of a gBlock gene fragment (Integrated DNA Technologies, Coralville, IA, USA) containing the target sequences. The qPCR quality controls, the description of the standard curves including the slope, intercept, *R* ^2^ and efficiency together with the limit of detection are shown in Table S3. Only amplification efficiencies between 90% and 110% were considered as acceptable for quantification.

The limit of detection was 6 gene copies per reaction for *E. coli* and 80 gene copies per reaction for 16S rRNA gene.

### Scanning electron microscopy

For each time and microcosms, three plastic pellets were fixed with 2.5% glutaraldehyde in phosphate buffer at pH 7.4 at 4°C until processing (less than 3 weeks). Samples were successively washed with phosphate buffer at pH 7.4 (4 × 10 min), fixed with 1% of osmium tetroxide, washed with Milli‐Q water (4 × 10 min), and dehydrated with different EtOH solutions in water: 50% (1 × 10 min), 70% (ON), 80% (1 × 10 min), 90% (3 × 10 min), 96% (3 × 10 min), and 100% EtOH (3 × 10 min). Finally, the samples were dried using Emitech K850 critical point dryer, mounted on double‐coated carbon conductive tape and carbon coated to improve their conductivity. Scanning electron microscope (SEM) observation was done with a JEOL JSM 7001F (JEOL, Akishima, Japan) at the CCiTUB.

### Illumina 16S rRNA amplicon sequencing

Sample sequencing of two microcosms (MC2 and MC3) was performed using the Illumina MiSeq platform at the Genomics Unit of Centre for Genomic Regulation Core Facilities (CRG, Barcelona). The V4 region was amplified from DNA sample extracts using the primers from the Earth Microbiome Project [515F (Parada et al., 2016) (5′‐GTGYCAGCMGCCGCGGTAA‐3′) and 806R (Apprill et al., 2015) (5′‐GGACTACNVGGGTWTCTAAT‐3′)] (following IPUAC ambiguity codes for nucleotide degeneracy: Y = C, T; M = A, C; W = A, T; V = A, C, G; N = A, C, G, T). The PCR included a primer concentration of 0.2 mM and KAPA HiFi HotStart ReadyMix (Roche) in a final volume of 25 μL. Cycling conditions consisted of an initial denaturation of 3 min at 95°C, followed by 25 cycles of 95°C for 30 s, 55°C for 30 s, and 72°C for 30 s, and a final elongation step of 5 min at 72°C. Reactions were purified using AgenCourt AMPure XP beads (Beckman Coulter). The first PCR primers contained overhangs allowing the addition of full‐length Nextera adapters with barcodes for multiplex sequencing, obtaining libraries with approximately 450 bp insert sizes. Five μl of the first amplification was used as template for the second PCR with Nextera XT v2 adaptor primers in a final volume of 50 μL using the same PCR mix and thermal profile as for the first PCR with just 8 cycles. After the second PCR, 25 μL of the final product was used for purification and normalisation with SequalPrep normalisation kit (Thermo Fisher Scientific), according to manufacturer's protocol. Libraries were eluted and pooled for sequencing. Final pool libraries were analysed using Agilent Bioanalyzer or Fragment analyser High Sensitivity assay to estimate the quantity and check size distribution and were then quantified by qPCR using the KAPA Library Quantification Kit (KapaBiosystems) prior to sequencing with Illumina's Miseq 2 × 300 bp.

Sequencing included negative controls including blanks from the DNA extraction process, the DNA extracted from disinfected pellets, as well as from the DNA amplification. The data are available at Mendeley Data public repository (doi: 10.17632/zp6htysmy2.1).

### Bioinformatic analyses

Cutadapt was used to trim adapters, primers, barcodes and leading Ns from sequencing reads. Sequences were processed to amplicon sequence variants (ASV) using the default parameters of the Dada2 workflow (Callahan et al., 2016). Firstly, quality filtering and the trimming of sequences was set to 220 bp (for forward reads) and 175 bp (for reverse reads) with a maximum number of expected errors allowed per read set at two (EE = 2). This parameter has been shown to be a better filter than simply averaging quality scores (Edgar & Flyvbjerg, 2015). Filtered sequences were dereplicated, the forward and reverse reads were aligned and merged, chimeras were removed and an amplicon sequence variant (ASV) table was obtained. Taxonomy was assigned to the resulting ASVs using the SILVA SSU 138 reference database and was imported to the phyloseq R package for microbiome analyses. To obtain a more accurate profile of microbial communities, the ‘decontam’ (Davis et al., 2018) R package was used to remove sequences derived from contaminating DNA present in extraction or sequencing reagents. In addition, chloroplast and mitochondrial reads were removed.

### Data analyses

Microbial abundances were log_10_ converted and analysed by descriptive statistics and plotted using the statistical software R version 4.0.3 (R Development Core Team, 2016) through the RStudio interface including the packages ‘Rmisc,’ ‘reshape2’ and ‘ggplot2’ v. 3.0.1 (Wickham, 2007, 2016).

## RESULTS

In this experiment, we studied the colonisation of environmental plastic pellets by *E. coli* and marine bacteria in aquaria with controlled conditions. We sampled plastic pellets regularly for 4 weeks, and we measured the attachment and persistence of *E. coli* within the plastisphere using culture and molecular methods.

### Colonisation of marine bacteria on plastic pellets

#### Characterisation of the water

The seawater samples used in the experiment had a total organic carbon concentration of 2.8 ± 1.12 ppm, inorganic carbon concentration of 26.28 ± 2.62 ppm, and total nitrogen concentration of 23 ± 0.35 ppm, which remained relatively stable throughout the experiment (Table S1). The water temperature was maintained at 20 ± 2°C, with daily variations not exceeding 1°C. The salinity was around 38.5 ± 1 PSU, pH was 8.0 ± 0.1, and dissolved oxygen was 4.5 ± 0.3 mg/L.

**FIGURE 1:**
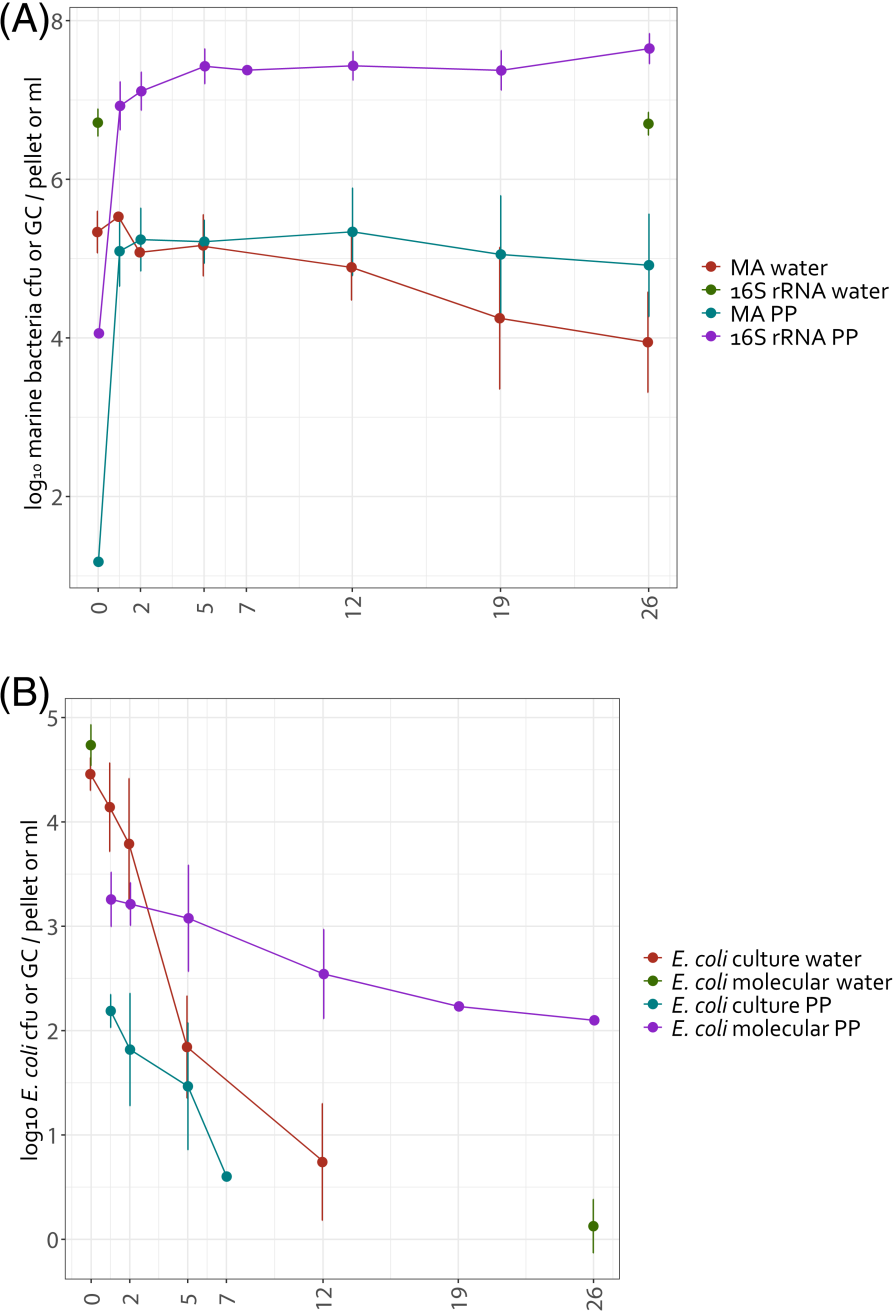
Abundance of marine bacteria detected by culture on marine agar and by qPCR (A) and *Escherichia coli* detected by culture on Chromocult and by qPCR (B) in water and plastic pellets over the course of the experiment (samples were collected after 1, 2, 5, 12, 19, and 26 days of incubation). Values represent the mean and standard deviation of the 4 microcosms. MA, Marine agar; PP, plastic pellets.

The initial bacterial population in seawater, as determined by the abundance of the 16S rRNA gene copies, was 6.3 × 10^6^ (±4.1 × 10^6^) gc mL^−1^, while the culturable bacteria on marine agar were 3.6 × 10^5^ (±3.7 × 10^5^) cfu mL^−1^ (Table S1), representing 6% of the 16S rRNA gene copies. The abundance of bacteria, as measured by qPCR, remained relatively stable throughout the experiment, with a final count of 5.9 × 10^6^ (±4.2 × 10^6^) gc of the 16S rRNA gene per ml at the end of the experiment. However, the abundance of culturable marine bacteria on marine agar showed a decrease ranging from 0.9 to 2.5 logs, reaching abundances of 5.0 × 10^4^ (±7.3 × 10^4^) cfu mL^−1^ depending on the microcosms (Figure 1A).

#### Characterisation of the plastisphere

The abundance of culturable marine bacteria in the plastic biofilm was measured by plating on marine agar after sonicating the biofilm. Within just 24 h, we detected 2.6 × 10^5^ (±2.7 × 10^5^) cfu per pellet, which corresponds to 3.3 × 10^3^ (±3.6 × 10^3^) cfu mm^−2^ corresponding to the average surface area of the plastic pellet (77 mm^2^). This concentration remained relatively stable throughout the experiment, with a slight decrease in 3 out of the 4 microcosms (Figure 1A). Quantification of the 16S rRNA gene by qPCR showed a substantial increase within 24 h, with 1.4 × 10^7^ (±1.1 × 10^7^) gc per pellet, corresponding to 1.8 × 10^5^ (±1.5 × 10^5^) gc mm^−2^. This concentration increased slightly further to 6.1 × 10^7^ (±6.2 × 10^7^) gc per pellet after 26 days (Figure 1A).

**FIGURE 2:**
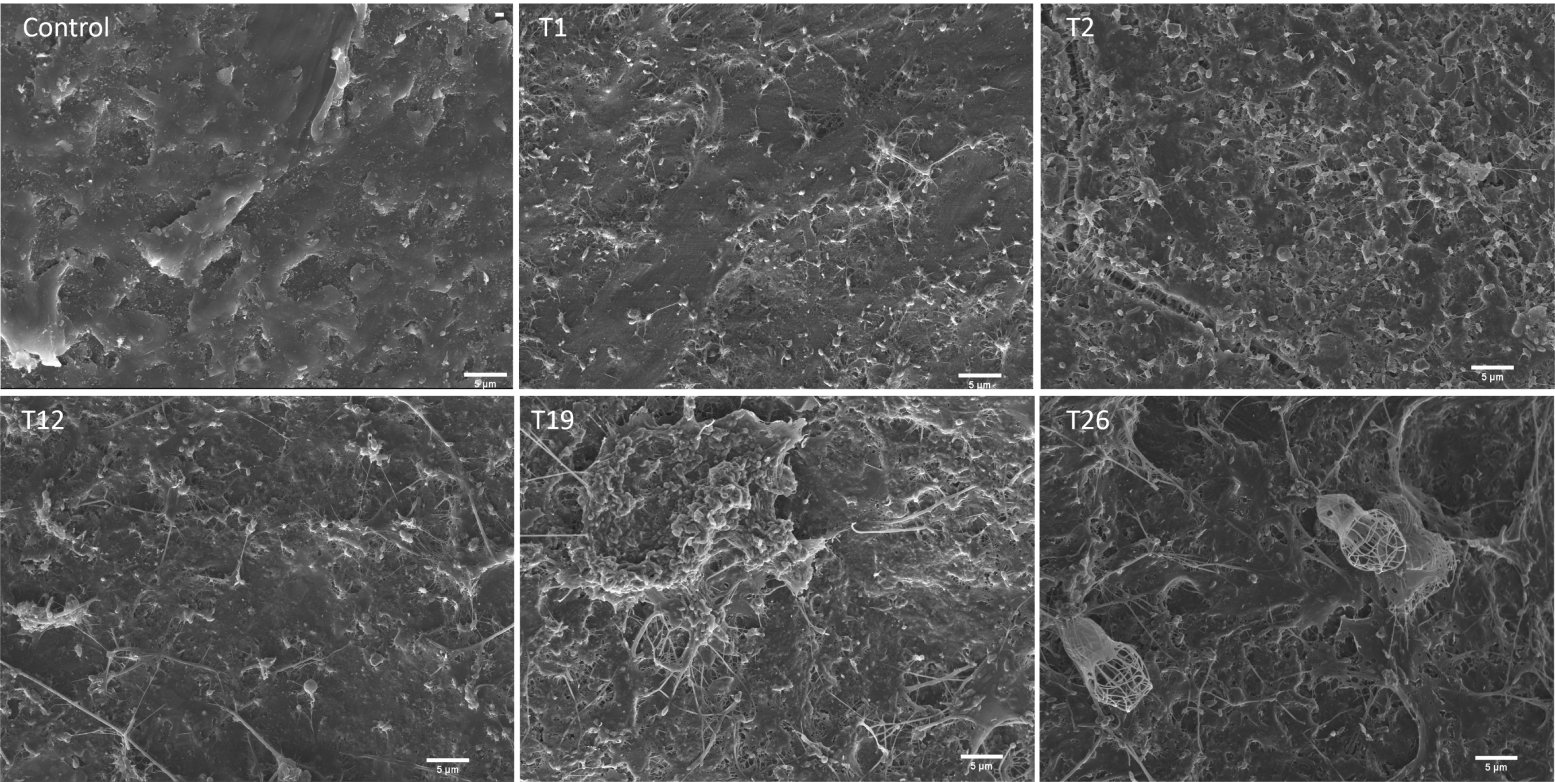
Scanning electron microscopy micrograph showing the colonisation process at different days (T1, T2, T12, T19, and T26) and control plastic pellet after disinfection and without incubation. Scale bars: 5 μm.

Scanning electronic microscopy images revealed the early colonisation observing single bacteria, predominantly coccobacillus, within the first 24 h (Figure 2). Some cells exhibited signs of division, indicating active growth, and the production of extracellular polymers for substrate attachment. On day 2, a higher density of bacteria with similar characteristics confirmed the colonisation of the plastic pellets. From day 5 to day 26, an increased presence of the exopolysaccharide matrix covering significant areas of the pellets was observed, along with the appearance of filamentous bacteria, clusters of cells characteristic of mature biofilms, and protists displaying morphological similarities to *Choanozoa* and *Ciliophora* (Figure 2). These protozoa are mainly bacterivores.

### *Colonisation of plastic pellets by* E. coli

We spiked the seawater with a mixture of three *E. coli* strains at an initial concentration of approximately 3.4 × 10^4^ (±2.2 × 10^4^) cfu mL^−1^, corresponding to 7.2 × 10^4^ (±6.0 × 10^4^) gc of the 16S rRNA gene of *E. coli* per ml. *E. coli* strains for MC1 and 2 were isolated from sewage, while strains for MC3 and 4 were isolated from plastic biofilms collected in coastal waters (Liang et al., 2023).

#### Characterisation of the water

In seawater, culturable *E. coli* was detected for 5 and 12 days, depending on the microcosms (Figure 1B). The inactivation of culturable *E. coli*, measured by the time required to reduce the initial population by 1 logarithm (T_90_), ranged from 1.2 to 3.6 days in seawater. However, the presence of *E. coli* DNA was detected until the last day of the experiment, with an average concentration of 2.1 (±2.0) gc of the 16S rRNA gene per ml, representing a reduction of approximately 4.0 to 5.3 logarithms over 26 days.

#### Characterisation of the plastisphere

In the plastic pellets, we detected culturable *E. coli* during 2–7 days from the microcosms (2 days in MC4, 5 days in MC1 and MC2, and 7 days in MC3) (Table 1). The highest abundance was typically observed after 2 days of incubation, with a mean of 4.3 × 10^2^ (±7.6 × 10^2^) cfu per pellet (corresponding to 5.6 (±10.0) cfu mm^−2^), followed by a gradual decrease (Table 1, Figure 1B). Moreover, the presence of *E. coli* DNA was detected for 5 days in MC4, 12 days in MC3, 19 days in MC2 and throughout the 26‐day experiment in MC1 (Table 1). The highest abundance was reached after 5 days, with average levels of 3.3 × 10^3^ (±2.9 × 10^3^) gc per pellet (4.2 × 10^1^ (±3.8 × 10^1^) gc mm^−2^), showing a slight decrease thereafter (Figure 1B, Table 1). In MC1, the abundance decreased to 1.3 × 10^2^ gc per pellet on the last day of the experiment.

**TABLE 1:** Maximum abundance of marine bacteria detected in plastic pellets of the 4 microcosms by culture in marine agar and by the quantification of the 16S rRNA gene.

	*E. coli* by culture		*E. coli* by qPCR		*HTS*
	Days detected	Max abundance Cfu per pellet Cfu per mm^−2^	Days detected	Max abundance	Days detected
MC1	5	3.2 × 10^2^ 4.2	26	5.6 × 10^3^ 72.8	NA
MC2	5	2.0 0.03	19	5.9 × 10^3^ 76.2	2
MC3	7	1.2 × 10^2^ 1.6	12	1.5 × 10^3^ 19.9	12
MC4	2	1.6 × 10^3^ 20.5	5	2.5 × 10^3^ 32.6	NA

*Note*: Days during how long *E. coli* could be detected by culture, qPCR, and high‐throughput sequencing together with the maximum densities observed. Data are shown by cfu and gc per pellet and cfu and gc per mm^−2^.

Abbreviation: NA, Not applicable.

### Taxonomic composition of the bacterial communities colonising the pellets

The microbial communities of MC2 and MC3 were analysed using high‐throughput sequencing of the 16S rRNA gene. A total of 2,475,520 reads were obtained after denoising and quality filtering the raw sequencing data. In MC2, the number of amplicon sequence variants (ASVs) varied between time points, with 415 ASVs on day 1, 751 ASVs on day 5 and a decrease to 373 ASVs at day 26. However, in MC3, the number of unique microbial taxa (ASVs) increased from 185 ASVs on day 1 to 424 on day 26. (Table S4). We focused our analysis on sequences affiliated with the domain Bacteria, as the detection of Archaea in the plastic biofilms was low (with 233 reads in MC2 and just 10 reads in MC3).

**FIGURE 3:**
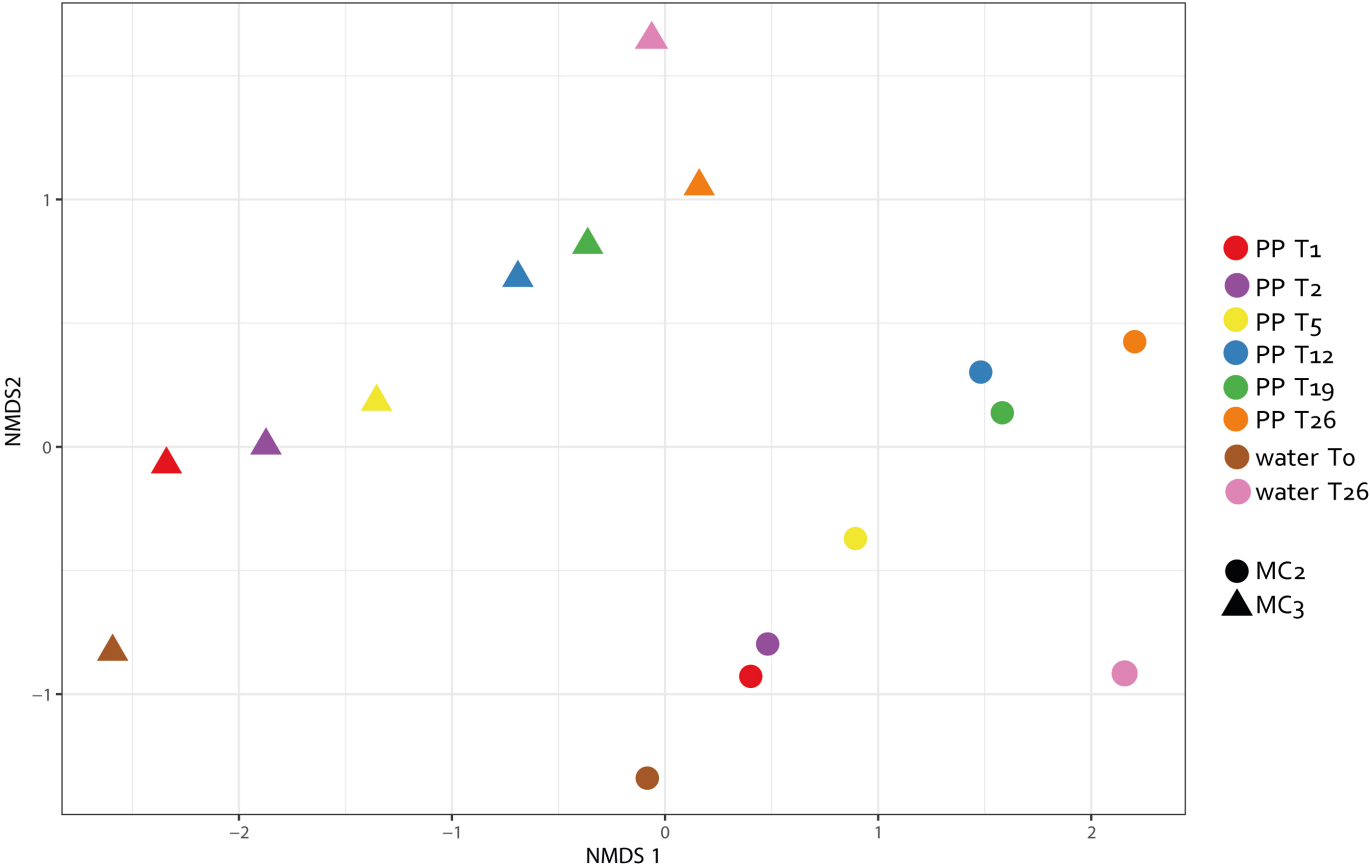
Non‐linear multidimensional scaling representation of the samples of plastic pellets (PP) at different days (T1, T2, T5, T12, T19, and T26) of the two different microcosms (MC2 and MC3) and water.

Distinct bacterial communities' structure was observed between MC2 and MC3 visualised by non‐metric multidimensional scaling (nMDS) of Beta‐diversity (Bray‐Curtis) coefficients (Figure 3) and by hierarchical clustering analysis (Figure S2). Besides, within each microcosm, two clusters were clearly defined (i) an initial biofilm cluster comprising the biofilm communities from days 1, 2 and 5 as well as the initial water sample, and (ii) a mature biofilm cluster consisting of the microbial communities from pellets collected on days 12, 19, and 26, along with the microbial community from water on day 26 (Figure S2). This clustering pattern was consistent in both microcosms.

#### Taxonomic composition of the water

The microbial community composition of the water of MC2 and MC3 exhibited notable differences (Figure S3). The taxonomic composition of both bacterial communities was predominantly composed of the phylum Proteobacteria, accounting for 85% and 86% of the total reads (Figure S3A). Within Proteobacteria, in MC2, 41% of the reads belonged to class Alphaproteobacteria, while 44% were classified as Gammaproteobacteria (Figure S3B). Whereas in MC3, within Proteobacteria, the class Gammaproteobacteria was the most abundant, accounting for 80% of the reads, while the class Alphaproteobacteria class represented only 6% of the reads. (Figure S3B). In MC2 at the end of the experiment, the bacterial community had shifted and Proteobacteria accounted for 84% of the identified affiliations, with the genus *Porticoccus* (Gammaproteobacteria class) representing 50% of the reads. Remarkably, this genus represented only 0.08% of the reads at the beginning of the experiment. In fact, just 20% of the ASVs were shared in water from T0 and T26 (Figure S3E, S4). Interestingly, the *Escherichia*‐*Shigella* group which accounted for 2% of the reads at day 0, was not detected after 26 days, although it could be detected using qPCR.

Within MC3, *Escherichia‐Shigella* group comprised 21% of the reads at the beginning of the experiment, followed by the genera *Marinobacterium* (12%) and *Thalassotalea* (11%) (Figure S3E). After 26 days, there was a notable shift in the microbial community of MC3. In fact, just about the 8% of the ASVs were shared between the microbial community of T0 and T26 (Figure S4). It is worth noting that neither the phylum Campylobacterota nor the *Escherichia*‐*Shigella* group was detected at the end of the experiment, indicating a significant change in their relative abundance over time.

#### Taxonomic composition of the plastisphere

In the plastic pellets from MC2, the Proteobacteria phylum accounted for 91 and 88% of the reads during the first 2 days (Figure S3A). During this period, the order Enterobacterales was outstanding representing 59% and 44% of the reads. However, its abundance gradually declined, becoming 12% of the reads at 5 day and ultimately representing only 0.3% of the ASVs at T26 (Figure S3C). The primary genus identified was *Alteromonas*, which comprised 10% of the reads until day 5 but decreased to <1% on day 26. The group *Shigella*‐*Escherichia* represented the 0.06% and 0.02% of the sequences on day T1 and day T2; meanwhile, it was not detected after. On day 5, there was an increase in diversity at the phylum level, with Proteobacteria representing 69% of the reads, Bacteroidota and Planctomycetota accounting for 10% of the reads each, and Bdellovibrionata comprising 4% of the reads. The latter phylum consists of obligate predators that feed on bacteria. By day 12, the alpha diversity had decreased compared to day 5 (Table S4). The main orders identified were Pseudomonadales (22%), Rhodobacterales (19%), Flavobacteriales (14%), and Caulobacterales (8%) (Figure S3C). The abundance of order Pseudomonadales continued to increase, becoming the dominant class by day 26 and representing 77% of the reads. Within this order, the genus *Halioxenophilus* became the most abundant (56%), followed by an unclassified genus of the Songiibacteraceae family (14%). In the water microbial community, Pseudomonadales order also dominated (61%), but the most relevant genus was *Porticoccus*.

On the pellets of MC3, the Proteobacteria phylum accounted for 89% and 88% of the reads during the first 2 days. The reads belonging to the Enterobacterales and Pseudomonadales orders dominated during the first 5 days. Specifically, Enterobacterales accounted for 47%, 35%, and 25% of the reads on days 1, 2, and 5, respectively. The group *Shigella*‐*Escherichia* was detected until day T12 representing the 0.01% of the sequences in days T1 and T2 and 0.04% in T5. Pseudomonadales represented 35%, 40%, 24% of the reads on the same respective days (Figure S3C). After day 5, the abundance of Enterobacterales decreased, representing only 3% of the reads on day 26. However, *Shigella*‐*Escherichia* was still detected until day T12 representing the 0.003% of the sequences. Pseudomonadales remained relatively constant, representing between 24% and 39% of the reads (Figure S3C). During the initial days (1 and 2), the most prevalent genera were *Thalassotalea* (Enterobacterales) (26%–16%) and *Aestuariicella* (Alteromonadales) (25%–27%). By day 5, although both genera were still the dominant, the global diversity increased, increasing the presence of the Flavobacteriales order from Bacteroidota phylum (Figure S3C). On day 12, *Thalassotalea* and *Aestuariicella* decreased to 0.6% and 3% of the reads, respectively. The genus *Methylophaga* (Nitrosoccales order) (16% of the reads), *Marinobacter* (Alteromonadales order) (12%) (known for its involvement in hydrocarbon degradation), and a genus from the Flavobacteriales order (16%) increased in abundance. Similar main genera were detected on day 19, with the addition of *Alcanivorax* (11%). By day 26, a higher diversity was observed, with *Marinobacter* being the most represented genus. Additionally, an increase in the presence of the Planctomycetota phylum was detected. For a more detailed taxonomy for all samples at different hierarchy levels, see the Krona diagrams in Figure S1.

We compared the shared ASVs between water and pellets separating between early and late biofilm as we could define a clear separation by hierarchical clustering (Figure S2). In MC2, we identified 244 ASVs that were shared between water and early biofilm pellets (T1, T2 and T5), while 336 ASVs were just detected in water and 37 ASVs were exclusively detected in plastic pellets at all three sampling times (Figure S4). Notably, pellets collected at T5 exhibited a higher number of shared ASVs with the water microbial community compared to the pellets collected in T1 and T2 (105, 32 and 12 ASVs, respectively). Within MC3, we found 104 shared ASVs between water and early biofilm pellets, with 325 ASVs exclusively detected in water and 43 ASVs exclusively detected in pellets at the three sampling times, without being detected in water (Figure S4). Regarding the late biofilm in MC2, we observed 181 shared ASVs between pellets collected at T12, T19 and T26, and the water sample from T26. Additionally, 414 ASVs were exclusively detected in water, while 33 ASVs were exclusively detected in pellets across the three sampling times (Figure S4). In MC3, we found 135 shared ASVs between the three sampling times and water, and 74 ASVs exclusively present in water and 72 ASVs exclusively detected in pellets across all three sampling times (Figure S4).

## DISCUSSION

The increase in plastic debris in coastal waters, resulting from human mismanagement, has led to the formation of persisting floating surfaces that are quickly colonised by microorganisms. One of the risks associated with the plastisphere is the potential for carrying microbial pathogens. Potential pathogens, such as members of *Campylobacteracea*, *Enterobacteriaceae, Mycobacterium sp, Pseudomonas*, or *Vibrio*, have already been identified in the plastisphere relying on high‐throughput sequencing (Jiang et al., 2018; Li et al., 2022; McCormick et al., 2014; Wu et al., 2019; Zettler et al., 2013). Other studies have used culture methods, specifically targeting *Vibrio* spp. and Enterobacteria, to detect ‘active’ bacteria and facilitate further strain identification (Kirstein et al., 2016; Liang et al., 2023; Silva et al., 2019). In this study, we investigated the colonisation of plastic pellets by marine bacteria and environmental strains of *E. coli*, to be used as a proxy for faecal pathogens, as commonly employed in water quality management. The experiments were conducted in seawater aquaria under stable conditions. The plastic pellets were collected from a polluted beach and consisted of a mixture of 80% PE and 20% PP, with an average surface of 77 mm^2^. The *E. coli* strains were obtained from raw sewage and from plastic samples from coastal waters.

We observed that seawater bacteria rapidly colonised the plastic pellets within 24 h, dividing and generating exopolysaccharide substances, while new bacteria attached to the pellets. Within 2 days, bacterial populations reached densities ranging from 4.5 × 10^4^ to 6.8 × 10^5^ gc of the 16S rRNA gene mm^−2^, remaining stable over the course of 26 days, reaching values of 2.5 × 10^5^–5.0 × 10^5^ gc of the 16S rRNA gene mm^−2^. The highest density observed was 2.0 × 10^6^ gc per mm^−2^, equivalent to 1.5 × 10^8^ gc per pellet. These densities were similar to those found in biofilms from coastal plastics ranging from 1.5 × 10^5^ to 8.7 × 10^6^ gc of the 16S rRNA mm^−2^ (Liang et al., 2023) and between 1.1 × 10^3^ and 1.9 × 10^5^ cells mm^−2^ (Dussud et al., 2018) (a cell count can be assumed to 8 gc according to the mean number of 16S rRNA copies per cell). Similar abundances were also observed in colonisation experiments which detected around 10^4^–10^5^ cells mm^−2^ (Odobel et al., 2021; Schlundt et al., 2020). The surrounding water aquaria exhibited a concentration of 6.0 × 10^6^ gc per ml^−1^, indicating that the surface of one plastic pellet contained 25 times the bacteria found in 1 mL of water.

*E. coli* was attached to plastic pellets within the first day of incubation, and its presence was confirmed by culture for a period of 2 to 7 days. The density of attached culturable *E. coli* varied among the microcosms, ranging from a maximum concentration of 2 cfu per pellet to 1.6·10^3^ cfu per pellet. *E. coli* attached to plastic pellets and remained ‘active’ for at least 7 days. The observed variations in the *E. coli* attachment and persistence could not be solely attributed to their source since differences were observed within the microcosms even when strains originated from the same source. This means, that the strains used did not show a different capability of forming biofilms on plastics under those conditions. Moreover, environmental factors cannot account for these variations, as they remained consistent across all four microcosms. *E. coli* could also be detected using qPCR methods during 5–26 days, depending on the microcosms. However, the qPCR results represent viable cells but also cells in viable but non‐culturable state or cells that are already dead.

The initial concentration of *E. coli* in the water aquaria ranged from 1.1 × 10^4^ to 6.4 × 10^4^ cfu ml^−1^, these values are similar to those found in poorly treated sewage effluent (Carrey et al., 2021). The inactivation of culturable *E. coli* in the water measured through the T_90_ was within 1.2 to 3.6 days, although slightly higher still consistent with observations from other experiments ranging from 0.1 to 2.9 days (Jeanneau et al., 2012; Sagarduy et al., 2019). Although *E. coli* could be detected in the water using culture methods for 5 to 12 days, the rate of inactivation was faster compared to the biofilm. Additionally, on the final day, when *E. coli* was detected on plastic pellets using qPCR, the concentration on the pellets was higher than in the water, indicating a slower decline in biofilms compared to the water. The decrease in *E. coli* abundance is expected in water since faecal bacteria are adapted to persist in the digestive tracts with stable conditions such as temperature, light, pH, and redox conditions, as well as a high concentration of nutrients. Therefore, when gut‐adapted bacteria encounter a harsh environment like seawater, they do not persist for long. These findings highlight that plastic biofilms can act as a protective environment for faecal bacteria like *E. coli* (Rodrigues et al., 2019). Although its dynamics may depend on the biofilm bacterial community or stochastic factors. In fact, for example, after performing an incubation experiment, researchers detected the attachment of *E. coli* in wood particles, but not in neither high‐density PE nor tyre wear particles (Song et al., 2020).

Despite the growing number of studies focusing on the plastisphere, there is a disparity in the findings among them. For instance, some studies have identified variations between colonised surfaces, while others have not (Pinto et al., 2019; Wright et al., 2021). And certain studies have reported a higher diversity of microbial communities than water, while others describe a less diverse community (Pinto et al., 2019; Wright et al., 2021). Explaining these differences may be challenging due to the involvement of multifactorial parameters that influence each colonisation process. These parameters encompass both deterministic and stochastic processes (Niederdorfer et al., 2021), further complicating the interpretation of the results. Thus, the variability observed in the plastisphere can be attributed to various factors from experimental design, to geography, temporal, substrate, and environmental differences (Amaral‐Zettler et al., 2020; Wright et al., 2021). These include dynamic environmental conditions such as salinity, temperature, light, and turbidity, as well as the diversity of the initial microbial community present in the water. Interactions between early colonisers, the presence of grazers, the experimental methodology, and characteristics of substrate such as polymer type, substrate weathering, and plastic additives have been reported to also contribute to the variability (Wright et al., 2021). In this experiment, the environmental conditions, substrate, and experimental methodology were the same. Therefore, the observed differences may be explained by the autochthonous water communities that colonise the biofilm or by stochastic factors what may also influence the attachment and evolution of *E. coli* in plastic biofilms. Besides, although a biofilm acts as protective environment, which can shelter bacteria for longer including faecal bacteria, it is also a full ecosystem with different communities interacting and trying to survive. Therefore, each biofilm may have different evolution considering adsorption of biomolecules during the biofilm conditioning (Bhagwat et al., 2021), water autochthonous bacterial community, environmental conditions, bacterial early interactions or even stochastically. *E. coli* becomes part of the plastisphere, but the concentration decreases over time; however, the persistence of *E. coli* DNA on plastic pellets for a more extended period compared to seawater implies that plastic surfaces might serve as a reservoir or provide a substrate for the retention of microbial genetic material.

When comparing the plastisphere of two microcosms, notable differences were present in the microbial communities, which can likely be attributed to variations in the microbial community found in the surrounding water which become the seed of the colonisation process. These bacterial communities were different to those observed in environmental plastics from Liang et al. (2023). However, the bacteria that attached to and generated the biofilm were less abundant in the water. Probably bacteria preferring a biofilm state than a planktonic state, become selected positively when they find a surface where they can attach and thrive. Furthermore, a clear distinction in the composition of the biofilm community was observed within the first 5 days of incubation and after 12 days. Other studies have also observed differences in microbial communities of early (<7 days incubation) or late (>7 days incubation) colonisation experiments (Wright et al., 2021). Normally, Proteobacteria dominate the earlier time points, whereas Bacteroidetes increased in late time points (Wright et al., 2021). Our results confirm that the microbial community of the plastisphere is primarily influenced by the community of the surrounding environment, although it evolves differently along the time detecting two clear different colonisation stages.

In general, the plastisphere is characterised by the dominance of Proteobacteria phylum, followed by Bacteroidetes and Planctomycetes (De Tender et al., 2017; Oberbeckmann et al., 2016; Wu et al., 2019). Additionally, bacteria known for their potential to degrade plastics or hydrocarbons, such as *Alcanivorax* sp., *Aestuariicella* sp., *Marinobacter* sp., and *Alteromonas* sp. have been commonly identified in plastics (Dussud et al., 2018; Wright et al., 2021). We also observed the presence of these bacteria primarily in MC3.

In our experiment, *E. coli* attachment and inactivation not only was measured by culture and by qPCR but also the group *Escherichia*‐*Shigella* could be detected by high throughput sequencing (HTS) during 2 or 12 days, similar to the detection observed by culture and shorter than the observed by qPCR. The difference in detection between qPCR and HTS can be explained because using specific primers for *E. coli* 16S rRNA gene qPCR we are selecting *E. coli* 16S rRNA gene among the others, whereas with HTS the amplification of 16S rRNA gene of the high abundant bacterial community of the plastisphere masks the low concentration of *E. coli* sequences. This finding can help in corroborating that if *E. coli* is found in plastics by HTS, it is probably in culturable state, so it may still be health concern.

Our study highlights the potential role of biofilms as reservoirs for *E. coli* and indicates that biofilms may contribute to the persistence and survival of faecal bacteria in aquatic systems. However, further research is necessary to fully understand the mechanisms and implications of the differences in *E. coli* dynamics (attachment and inactivation) in biofilm environments and of other potential pathogens, beyond the structure of the developing biofilm microbial communities.

## CONCLUSIONS

Plastic debris in coastal waters serves as a persistent floating surface that quickly becomes colonised by marine bacteria, forming a biofilm, which were already detected 24 h after the introduction of the plastic pellets in the seawater. Faecal bacteria, specifically *E. coli*, were found to attach to and persist within the plastic biofilms for 2 to 7 days in a cultivable state. This suggests that plastic biofilms may facilitate the survival and transportation of faecal bacteria in aquatic environments. *E. coli* was detected during a similar period by high‐throughput sequencing technology but was detected much longer using qPCR detecting DNA of probably dead or viable but not cultivable cells. Despite *E. coli* attached to pellets in all the microcosms, different concentration, and patterns have been observed between them showing that the colonisation and evolution of the biofilm may depend on the global bacterial community or stochastic factors. The composition of the microbial communities within the biofilms was primarily influenced by the surrounding environment. Temporal shifts were observed within the first 5 days of incubation and after 12 days, indicating changes in the community structure over time. Bacteria known for their plastic or hydrocarbon‐degrading potential, such as *Alcanivorax* sp., *Aestuariicella* sp., *Marinobacter* sp., and *Alteromonas* sp., were commonly found. Bacterivore species, such as those from the Bdellovibrionota phylum, were detected, and protozoa like *Choanozoa* and *Ciliphora* appeared mainly in the late stages of the biofilm formation limiting biofilm growth. Therefore, the observed differences in *E. coli* colonisation may be explained by the autochthonous communities that colonise the biofilm or by stochastic factors. Further research is needed to develop comprehensive models of *E. coli* colonisation and persistence on plastics. The results of this study have implications for environmental monitoring, risk assessment, and the development of mitigation strategies to address the growing problem of plastic pollution in our oceans.

## AUTHOR CONTRIBUTIONS

**Elisenda Ballesté:** Conceptualization (lead); formal analysis (lead); funding acquisition (lead); investigation (lead); methodology (lead); project administration (lead); supervision (lead); writing – original draft (lead). **Hongxia Liang:** Formal analysis (equal); investigation (equal); methodology (equal). **Laura Migliorato:** Data curation (equal); methodology (equal). **Laura Sala‐Comorera:** Data curation (equal); writing – review and editing (supporting). **Javier Méndez:** Software (equal); validation (equal); visualization (equal); writing – review and editing (equal). **Cristina Garcia‐Aljaro:** Investigation (equal); methodology (equal); resources (equal); writing – review and editing (equal).

## CONFLICT OF INTEREST STATEMENT

The authors declare no conflicts of interest.

## Supporting information


**Table S1.** Physicochemical and bacterial characteristics of the seawater of the different microcosms (MC1, MC2, MC3, MC4).
**Table S2.** Information of oligonucleotide primers and probes of molecular markers using real‐time quantitative PCR.
**Table S3.** Performance characteristics for all qPCR assays.
**Table S4.** Total ASVs and alpha‐diversity (Chao and Shannon index) of the microbial communities of the plastic pellets at different times (from T1 to T26) and water at T0 and T26 of microcosmos 2 and 3.
**Figure S1.** Representation of the experiment developed in this study to evaluate the colonization of plastic pellets. MC, Microcosms.
**Figure S2.** Hierarchical clustering analysis using Euclidean distance separating both microcosmos (MC2 and MC3) and early (T1, T2, T5) and late biofilm (T12, T19, T26).
**Figure S3.** Taxonomic affiliation of ASVs at considering Phylum (A), Class (B), Orders (C), Classes (D) and Genera (E) in pellets (MP) collected at different times (T1, T2, T5, T12, T19 and T26) on both microcosmos (MC2 and MC3) and water.
**Figure S4.** Venn diagrams showing the distribution and sharing of the different ASVs between water and pellets at different times considering an early biofilm (T1, T2, T5) and a late biofilm (T12, T19 and T26) in both microcosmos (MC2 and MC3).
**Figure S5.** Krona plots of the relative abundance reads of bacteria detected by 16S metabarcoding at all sampling times (T0, T1, T2, T5, T12, T19, T26) in water samples and plastic pellets (MP) from microcosmos 2 (MC2) and 3 (MC3). Taxonomic profiles are simultaneously displayed by hierarchy levels from kingdom to genus by selecting taxonomic depths: 1: Kingdom 2: Phylum 3: Class 4: Order 5: Family 6: Genus.

## Data Availability

The data that support the findings of this study are openly available in Mendeley Data public repository at doi:10.17632/zp6htysmy2.1.
