# Supplementary material for: Exploring plastic biofilm formation and Escherichia coli colonisation in marine environments
Source: Environ Microbiol Rep. 2024 Jun 24;16(3):e13308. doi: 10.1111/1758-2229.13308 (PMC11196126; doi:10.1111/1758-2229.13308)
Supplement: Supplementary file 1 — Table S1. Physicochemical and bacterial characteristics of the seawater of the different microcosms (MC1, MC2, MC3, MC4). Table S2. Information of oligonucleotide primers and probes of molecular markers using real‐time quantitative PCR. Table S3. Performance characteristics for all qPCR assays. Table S4. Total ASVs and alpha‐diversity (Chao and Shannon index) of the microbial communities of the plastic pellets at different times (from T1 to T26) and water at T0 and T26 of microcosmos 2 and 3. Figure S1. Representation of the experiment developed in this study to evaluate the colonization of plastic pellets. MC, Microcosms. Figure S2. Hierarchical clustering analysis using Euclidean distance separating both microcosmos (MC2 and MC3) and early (T1, T2, T5) and late biofilm (T12, T19, T26). Figure S3. Taxonomic affiliation of ASVs at considering Phylum (A), Class (B), Orders (C), Classes (D) and Genera (E) in pellets (MP) collected at different times (T1, T2, T5, T12, T19 and T26) on both microcosmos (MC2 and MC3) and water. Figure S4. Venn diagrams showing the distribution and sharing of the different ASVs between water and pellets at different times considering an early biofilm (T1, T2, T5) and a late biofilm (T12, T19 and T26) in both microcosmos (MC2 and MC3). Figure S5. Krona plots of the relative abundance reads of bacteria detected by 16S metabarcoding at all sampling times (T0, T1, T2, T5, T12, T19, T26) in water samples and plastic pellets (MP) from microcosmos 2 (MC2) and 3 (MC3). Taxonomic profiles are simultaneously displayed by hierarchy levels from kingdom to genus by selecting taxonomic depths: 1: Kingdom 2: Phylum 3: Class 4: Order 5: Family 6: Genus. [file EMI4-16-e13308-s001.zip › emi413308-sup-0001-Supinfo/emi413308-sup-0001-Supinfo.docx]

*Supplementary material*

|  | MC1 | MC2 | MC3 | MC4 |
| --- | --- | --- | --- | --- |
| Total Organic Carbon  (mg l^-1^) | 8.83 | 4.06 | 1.91 | 2.44 |
| Inorganic Carbon  (mg l^-1^) | 21.02 | 24.01 | 25.68 | 29.15 |
| Total Carbon  (mg l^-1^) | 29.85 | 28.07 | 27.59 | 31.59 |
| Total Nitrogen (mg l^-1^) | 0.01 | 0.64 | 0.02 | 0.04 |
| pH | 7.93±0.11 | 7.92±0.03 | 8.05±0.13 | 9.10±0.14 |
| Dissolved Oxygen (mg l^-1^) | 4.77±0.90 | 4.92±0.85 | 4.40±0.50 | 4.30±0.58 |
| PSU (mg l^-1^) | 38.56±0.25 | 38.25±0.32 | 41.22±0.86 | 37.36±0.04 |
| Marine bacteria in marine agar  (cfu ml^-1^) | 8.80·10^5^ | 1.14·10^6^ | 3.75E·10^5^ | 5.80·10^4^ |
| Marine bacteria 16S rRNA  (gc ml^-1^) | 6.52·10^6^ | 1.17·10^7^ | 1.77·10^6^ | 5.39·10^6^ |
| *E. coli* before spiking  (cfu 100 ml^-1^) | < 1 | < 1 | < 1 | < 1 |
| *E. coli* source | Sewage | Sewage | Coastal plastic | Coastal plastic |
| *E. coli* spiked  (cfu ml^-1^) | 2.94·10^4^ | 1.10·10^4^ | 6.40·10^4^ | 3.30·10^4^ |
| *E. coli* spiked  (gc ml^-1^) | 1.58·10^5^ | 1.72·10^4^ | 5.30·10^4^ | 6.07·10^4^ |

**Microcosmos**

Table S1. Physicochemical and bacterial characteristics of the seawater of the different microcosms (MC1, MC2, MC3, MC4).

Table S2. Information of oligonucleotide primers and probes of molecular markers using real-time quantitative PCR

| Assays | Primer/ probe | Sequence（5’-3’） | Concentration（nM） | Size of product（bp） | Reference |
| --- | --- | --- | --- | --- | --- |
| 16S rRNA gene sequencing | 27f | AGAGTTTGATCMTGGCTCAG | 400 |  | (Langendijk et al., 1995; Weisburg et al., 1991) |
|  | 1492r | TACGGYTACCTTGTTACGACTT | 400 |  |  |
| Bacterial 16S rRNA gene | 341F | CCTACGGGAGGCAGCAG | 1000 | 194 | (Muyzer et al., 1996; Muyzer et al., 1995) |
|  | 534R | ATTACCGCGGCTGCTGG | 1000 |  |  |
| *E. coli* | E.coli_F | CATGCCGCGTGTATGAAGAA | 300 | 96 | (Huijsdens et al., 2002) |
|  | E.coli_R | CGGGTAACGTCAATGAGCAAA | 300 |  |  |
|  | E.coli_P | FAM^a^-TATTAACTTTACTCCCTTCCTCCCCGCTGAA-TAMRA^b^ | 100 |  |  |

^a^ FAM: Carboxy-fluorescein, fluorescent reporter.

^b^ TAMRA: Carboxytetramethylrhodamine, quencher.

^c^ MGB: Minor Groove Binder, quencher.

^d^ NFQ: Non-fluorescent quencher.

Table S3**.** Performance characteristics for all qPCR assays

| Assay | R^2^ | Efficiency | Ct of no template controls | LOD^a^  (copies/reaction) | LOD^a^  (copies/pellet) |
| --- | --- | --- | --- | --- | --- |
| 16S rRNA gene | 0.996 - 0.998 | 94.0 – 107.0 % | 35.06 | 80 | 1.6 x 10^3^ |
| *E. coli* | 0.994 - 0.999 | 93.0 – 102% | Undetermined | 6 | 24 |

^a^ Limit of Detection

Table S4. Total ASVs and alpha-diversity (Chao and Shannon index) of the microbial communities of the plastic pellets at different times (from T1 to T26) and water at T0 and T26 of microcosmos 2 and 3.

| Sample |  | Total ASVs | Chao | Shannon |
| --- | --- | --- | --- | --- |
| Pellets MC2 | T1 | 415 | 415 | 3.2 |
|  | T2 | 363 | 363 | 3.7 |
|  | T5 | 751 | 751 | 4.9 |
|  | T12 | 348 | 348 | 4.3 |
|  | T19 | 675 | 675 | 3.9 |
|  | T26 | 373 | 373 | 2.5 |
| Water MC2 | 0 | 793 | 793 | 4.1 |
|  | 26 | 867 | 867 | 3.1 |
| Pellets MC3 | T1 | 185 | 185 | 3.3 |
|  | T2 | 229 | 229 | 3.6 |
|  | T5 | 302 | 302 | 3.8 |
|  | T12 | 347 | 347 | 3.7 |
|  | T19 | 334 | 334 | 3.9 |
|  | T26 | 424 | 424 | 4.5 |
| Water MC3 | 0 | 477 | 477 | 3.5 |
|  | 26 | 327 | 327 | 3.8 |


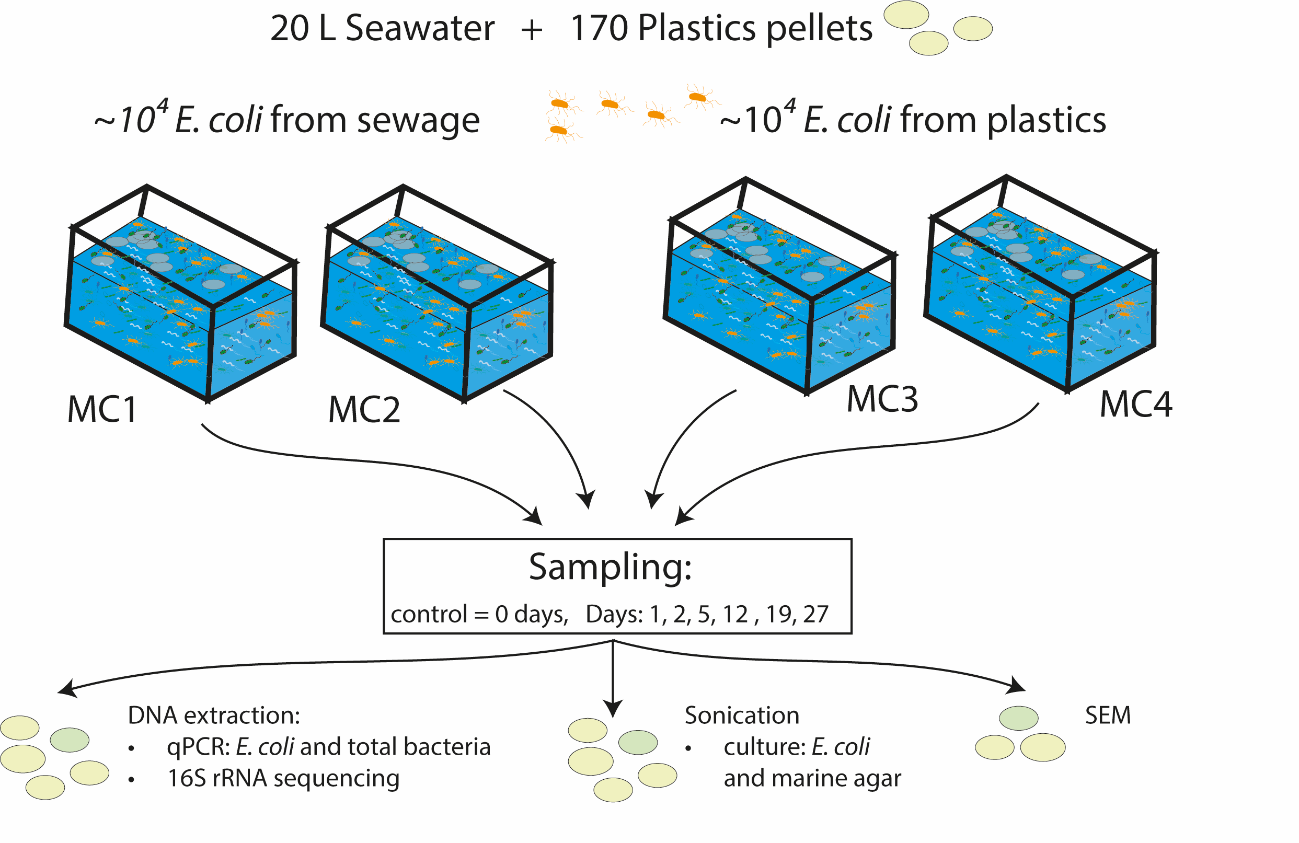


Fig S1. Representation of the experiment developed in this study to evaluate the colonization of plastic pellets. MC: Microcosms


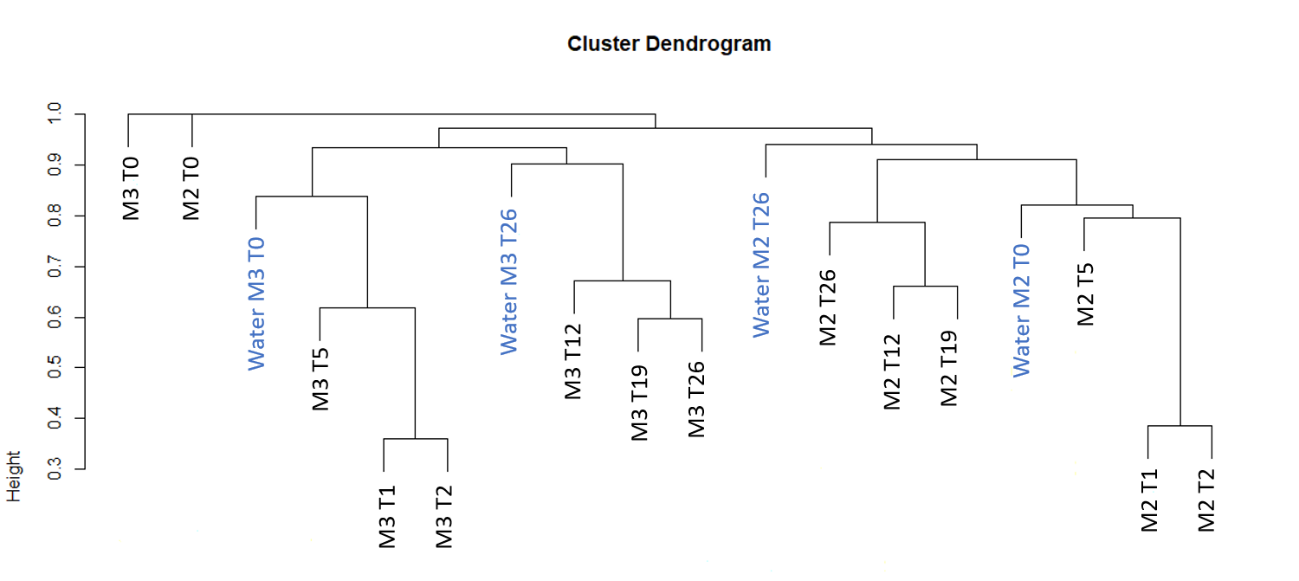


Fig S2. Hierarchical clustering analysis using Euclidean distance separating both microcosmos (MC2 and MC3) and early (T1, T2, T5) and late biofilm (T12, T19, T26).

| A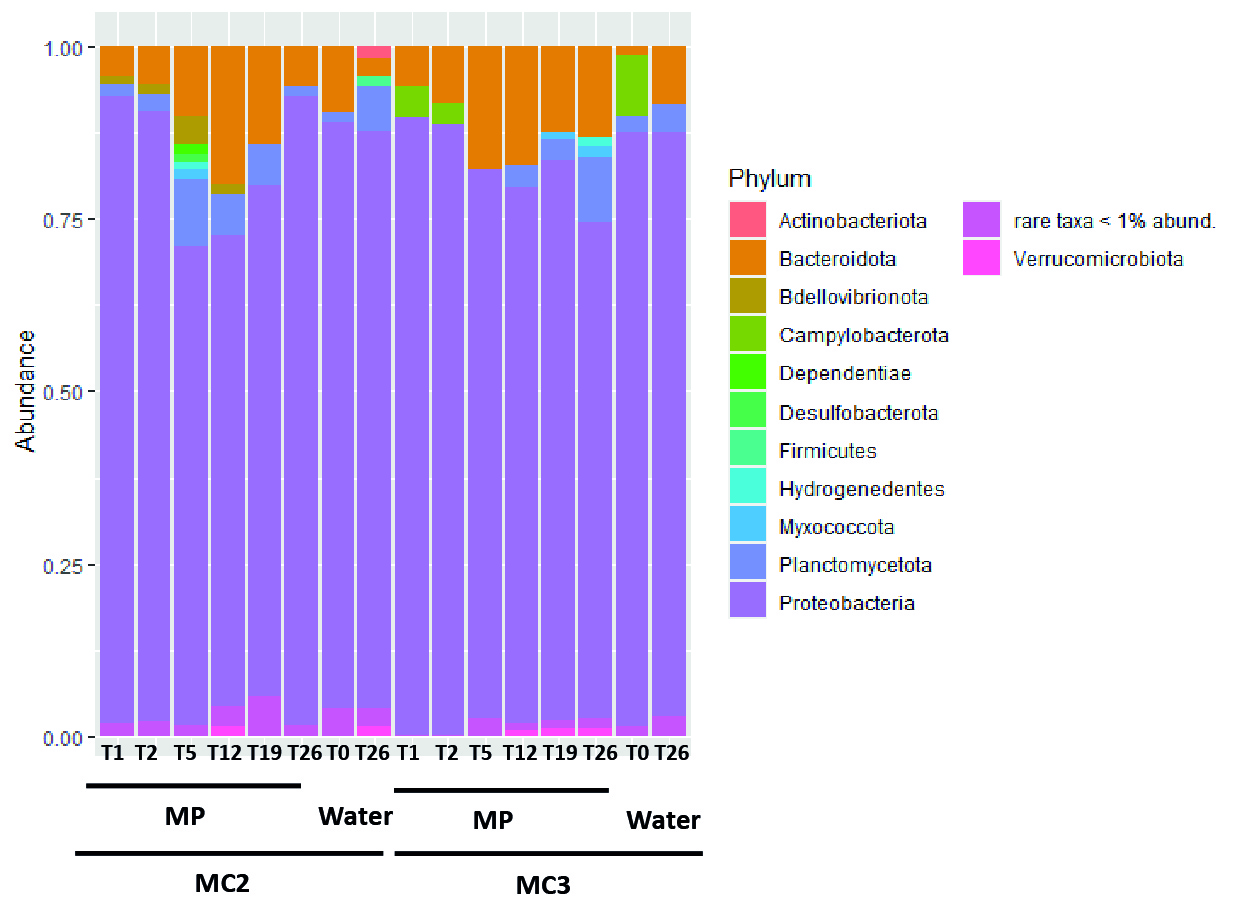 |
| --- |
| B  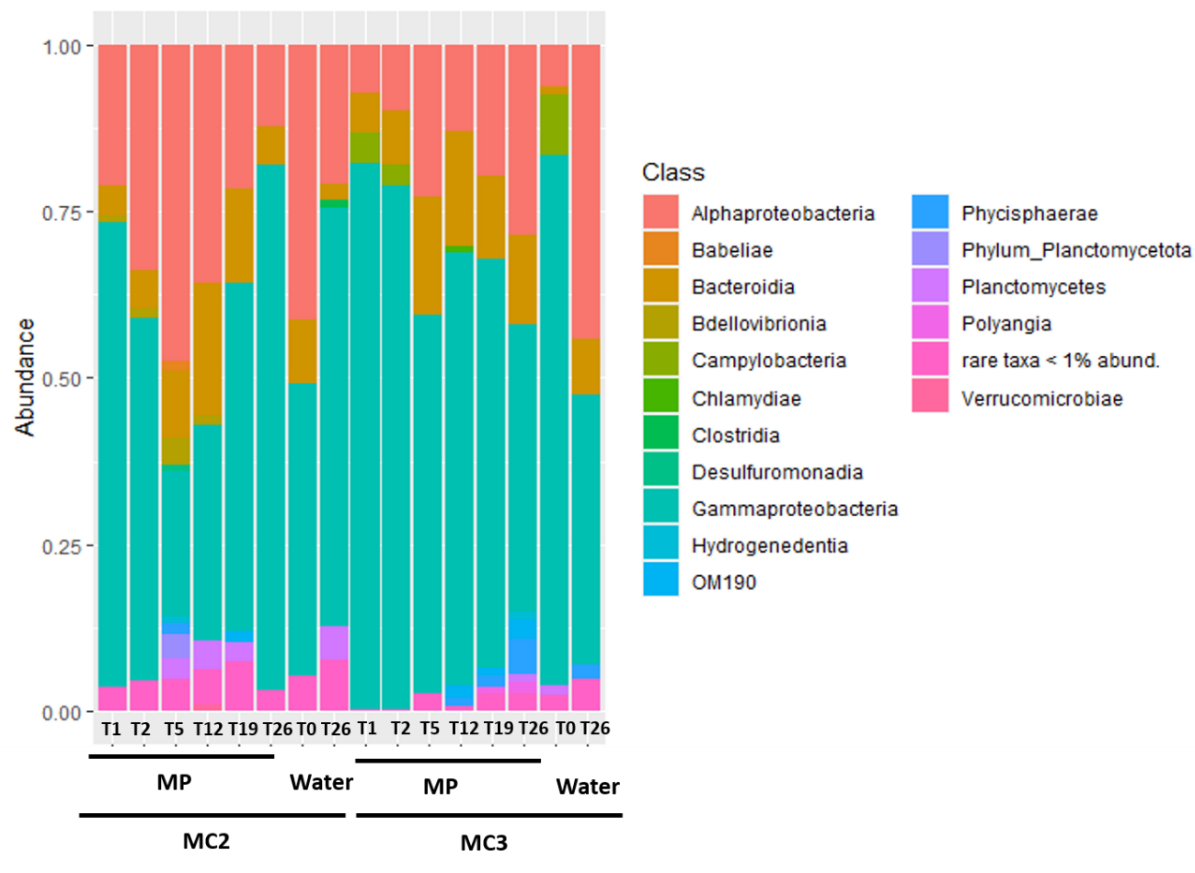 |
| C  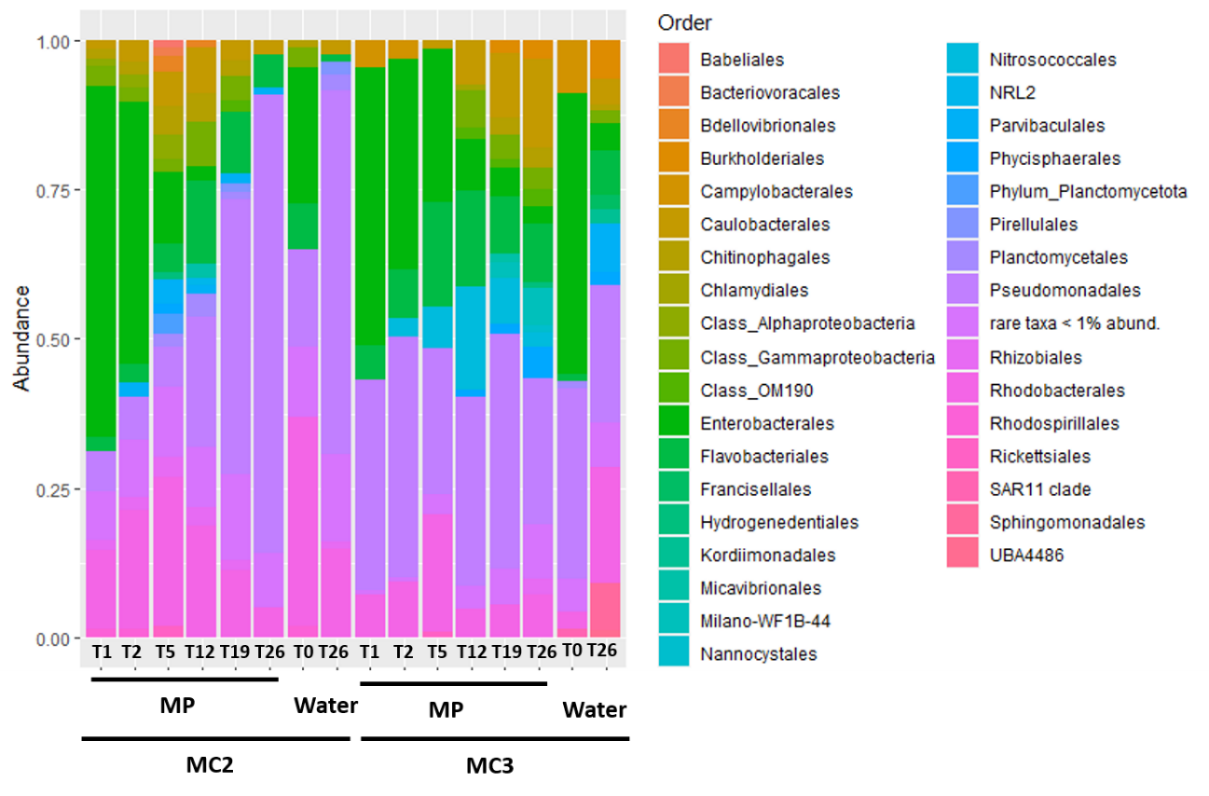 |
| D  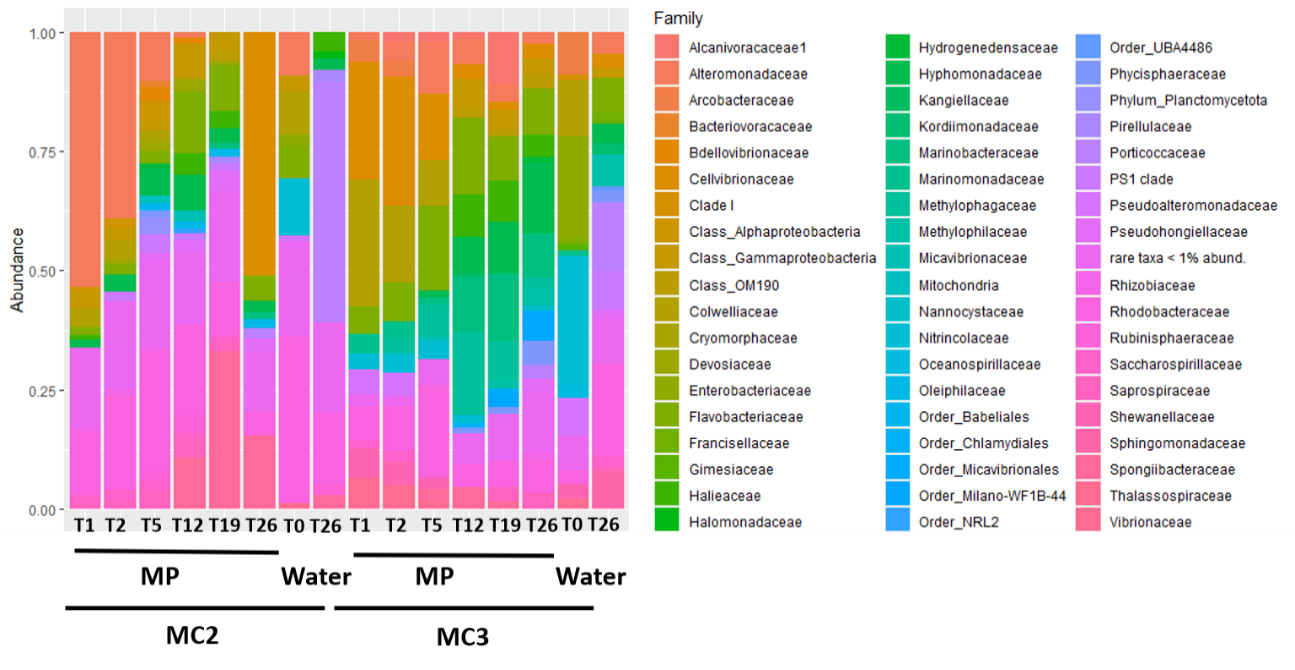 |


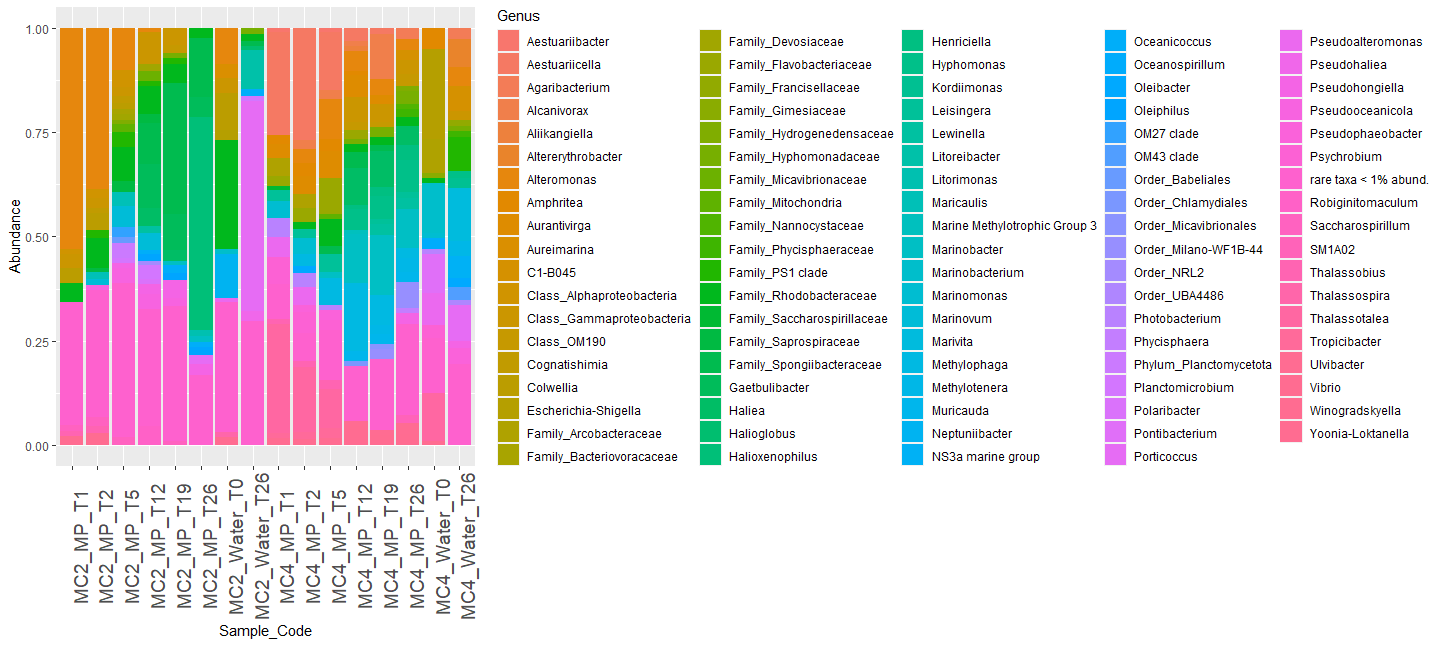


**T1 T2 T5 T12 T19 T26**

**T1 T2 T5 T12 T19 T26**

**MP**

**Water**

**T0 T26**

**T0 T26**

**MP**

**Water**

**MC2**

**MC3**

E

Fig S3. Taxonomic affiliation of ASVs at considering Phylum (A), Class (B), Orders (C), Classes (D) and Genera (E) in pellets (MP) collected at different times (T1, T2, T5, T12, T19 and T26) on both microcosmos (MC2 and MC3) and water.

|  | **MC2** | **MC3** |
| --- | --- | --- |
| **Water** | 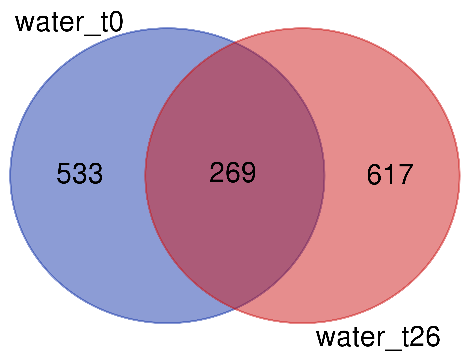 | 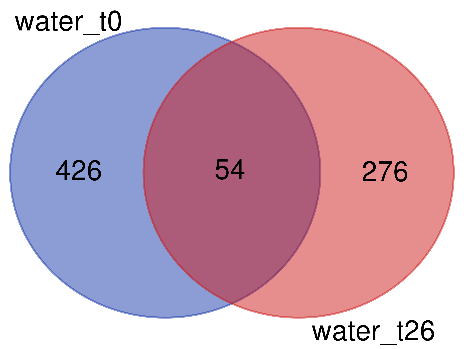 |
| **Early biofilm** | 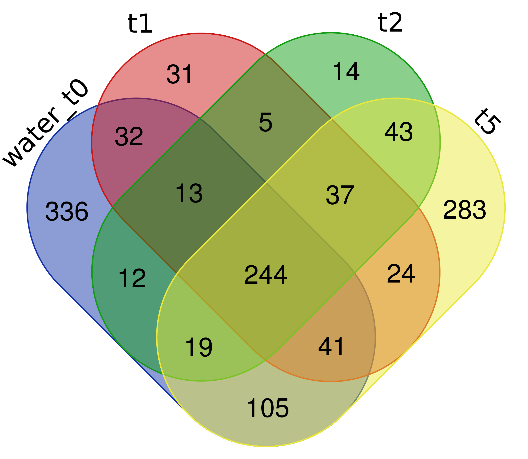 | 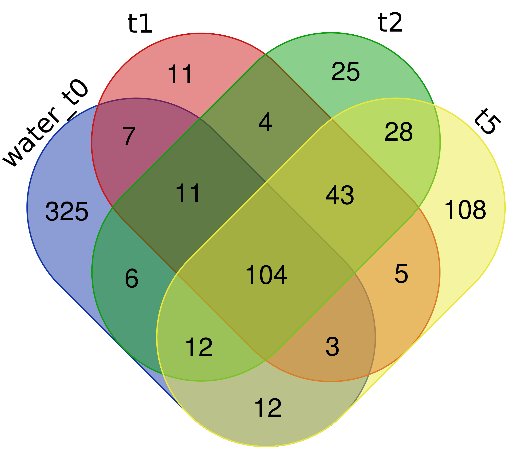 |
| **Late biofilm** | 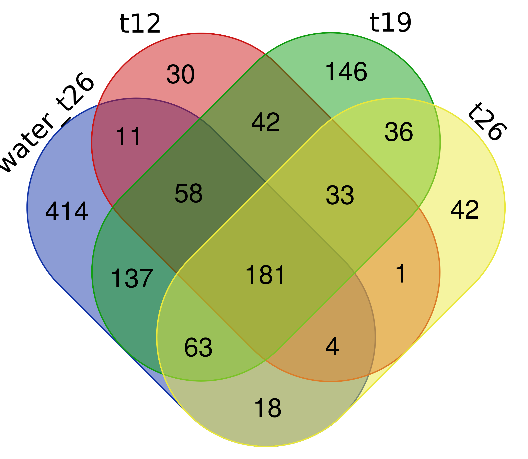 | 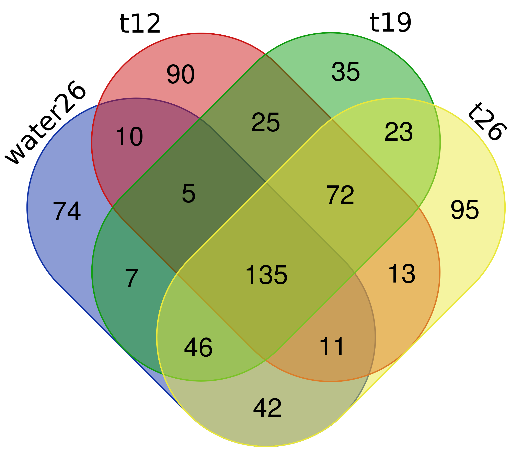 |

Fig. S4. Venn diagrams showing the distribution and sharing of the different ASVs between water and pellets at different times considering an early biofilm (T1, T2, T5) and a late biofilm (T12, T19 and T26) in both microcosmos (MC2 and MC3).

Fig. S5. Krona plots of the relative abundance reads of bacteria detected by 16S metabarcoding at all sampling times (T0, T1, T2, T5, T12, T19, T26) in water samples and plastic pellets (MP) from microcosmos 2 (MC2) and 3 (MC3). Taxonomic profiles are simultaneously displayed by hierarchy levels from kingdom to genus by selecting taxonomic depths: 1: Kingdom 2: Phylum 3: Class 4: Order 5: Family 6: Genus.

**REFERENCES**

Huijsdens, X.W., Linskens, R.K., Mak, M., Meuwissen, S.G.M., Vandenbroucke-Grauls, C.M.J.E., Savelkoul, P.H.M., 2002. Quantification of Bacteria Adherent to Gastrointestinal Mucosa by Real-Time PCR. J Clin Microbiol 40, 4423–4427. https://doi.org/10.1128/JCM.40.12.4423-4427.2002

Langendijk, P.S., Schut, F., Jansen, G.J., Raangs, G.C., Kamphuis, G.R., Wilkinson, M.H., Welling, G.W., 1995. Quantitative fluorescence in situ hybridization of Bifidobacterium spp. with genus-specific 16S rRNA-targeted probes and its application in fecal samples. Appl.Environ.Microbiol. 61, 3069–3075.

Muyzer, G., Hottentrager, S., Teske, A., Wawer, C., 1996. Denaturing gradient gel electrophoresis of PCR amplified 16s rDNA-A new molecular approach to analyze the genetic diversity of mixed microbial communities., in: Akkermans, A., van Elsas, J.D., de Bruijn, F. (Eds.), Molecular Microbial Ecology Manual. KluwerAcademic Publishing, Dordrecht, pp. 3.4.4.1-3.4.4.22.

Muyzer, G., Teske, A., Wirsen, C.O., Jannasch, H.W., 1995. Phylogenetic relationships ofThiomicrospira species and their identification in deep-sea hydrothermal vent samples by denaturing gradient gel electrophoresis of 16S rDNA fragments. Arch Microbiol 164, 165–172. https://doi.org/10.1007/BF02529967

Weisburg, W.G., Barns, S.M., Pelletier, D.A., Lane, D.J., 1991. 16S ribosomal DNA amplification for phylogenetic study. J.Bacteriol. 173, 697–703.
