# Supplementary material for: Exploring plastic biofilm formation and Escherichia coli colonisation in marine environments
Source: Environ Microbiol Rep. 2024 Jun 24;16(3):e13308. doi: 10.1111/1758-2229.13308 (PMC11196126; doi:10.1111/1758-2229.13308)
Supplement: Supplementary file 1 — Table S1. Physicochemical and bacterial characteristics of the seawater of the different microcosms (MC1, MC2, MC3, MC4). Table S2. Information of oligonucleotide primers and probes of molecular markers using real‐time quantitative PCR. Table S3. Performance characteristics for all qPCR assays. Table S4. Total ASVs and alpha‐diversity (Chao and Shannon index) of the microbial communities of the plastic pellets at different times (from T1 to T26) and water at T0 and T26 of microcosmos 2 and 3. Figure S1. Representation of the experiment developed in this study to evaluate the colonization of plastic pellets. MC, Microcosms. Figure S2. Hierarchical clustering analysis using Euclidean distance separating both microcosmos (MC2 and MC3) and early (T1, T2, T5) and late biofilm (T12, T19, T26). Figure S3. Taxonomic affiliation of ASVs at considering Phylum (A), Class (B), Orders (C), Classes (D) and Genera (E) in pellets (MP) collected at different times (T1, T2, T5, T12, T19 and T26) on both microcosmos (MC2 and MC3) and water. Figure S4. Venn diagrams showing the distribution and sharing of the different ASVs between water and pellets at different times considering an early biofilm (T1, T2, T5) and a late biofilm (T12, T19 and T26) in both microcosmos (MC2 and MC3). Figure S5. Krona plots of the relative abundance reads of bacteria detected by 16S metabarcoding at all sampling times (T0, T1, T2, T5, T12, T19, T26) in water samples and plastic pellets (MP) from microcosmos 2 (MC2) and 3 (MC3). Taxonomic profiles are simultaneously displayed by hierarchy levels from kingdom to genus by selecting taxonomic depths: 1: Kingdom 2: Phylum 3: Class 4: Order 5: Family 6: Genus. [file EMI4-16-e13308-s001.zip › emi413308-sup-0001-Supinfo/emi413308-sup-0002-FigureS5.html]

Javascript must be enabled to view this page.

magnitude

MC2\_MP\_T0
MC4\_MP\_T0
MC2\_MP\_T1
MC2\_MP\_T26
MC2\_Water\_T26
MC2\_MP\_T2
MC2\_MP\_T19
MC4\_MP\_T2
MC2\_Water\_T0
MC4\_MP\_T1
MC4\_Water\_T0
MC4\_MP\_T12
MC4\_MP\_T19
MC4\_MP\_T5
MC4\_Water\_T26
MC2\_MP\_T12
MC2\_MP\_T5
MC4\_MP\_T26

 1
 1
 1
 1
 1
 1
 1
 1
 .999999999999998
 1
 1
 1
 1
 1
 .999999999999997
 1
 .999999999999999
 1

 1
 1
 1
 1
 1
 1
 1
 1
 .999999999999998
 1
 1
 1
 1
 1
 .999999999999997
 1
 .999999999999999
 1

 .875
 1
 .908793323620929
 .910809936998104
 .837423615449289
 .88395882616201
 .740917880057422
 .884244358077712
 .850006666101058
 .894132791241564
 .859934059177119
 .778297291237797
 .808492875033002
 .796658158639832
 .845451992345421
 .681442307692308
 .692071995292303
 .718724708321588

 .875
 .375
 .696253529739744
 .787305248992931
 .627708393024053
 .544381220297082
 .523606187560426
 .786256249478244
 .436678100039996
 .820364127629474
 .796187729918264
 .648757719666551
 .612126358453272
 .568517511192937
 .404379829012939
 .323333333333333
 .216297017782378
 .433207234897742

 .6875
 0
 8.09367074947392E-04
 3.73118025882332E-03
 2.48939657966304E-04
 0
 5.9326751237805E-04
 1.15945793022846E-04
 1.87256838813677E-03
 1.97549116445645E-04
 3.47726525809153E-03
 9.68142999816562E-03
 2.25273117788513E-02
 1.85967799675486E-04
 6.55406861688579E-02
 0
 8.11671841074653E-05
 3.24233633726481E-02

 .6875
 0
 7.19437399953237E-04
 7.86431436285947E-05
 3.75757974288761E-05
 0
 1.24512934696628E-04
 0
 7.27211024519132E-05
 0
 4.45259575731233E-04
 0
 0
 0
 0
 0
 8.11671841074653E-05

 .6875
 0
 7.19437399953237E-04
 7.86431436285947E-05
 0
 0
 1.24512934696628E-04
 0
 7.27211024519132E-05
 0
 0
 0
 0
 0
 0
 0
 8.11671841074653E-05

 0
 0
 0
 0
 0
 0
 0
 0
 0
 0
 1.90825532456243E-04

 0
 0
 0
 0
 0
 0
 0
 0
 0
 0
 1.32517730872391E-04

 0
 0
 0
 0
 0
 0
 0
 0
 0
 0
 1.21916312402599E-04

 0
 0
 0
 0
 3.75757974288761E-05

 0
 0
 0
 3.57389397156613E-03
 8.92425188935806E-05
 0
 3.58890223537339E-04
 5.10161489300522E-05
 1.24231883355352E-03
 0
 4.66462412670815E-04
 9.64066608238387E-03
 2.24630907443218E-02
 1.3017745977284E-04
 6.55406861688579E-02
 0
 0
 3.23051460892813E-02

 0
 0
 0
 3.57389397156613E-03
 8.92425188935806E-05
 0
 3.58890223537339E-04
 5.10161489300522E-05
 4.72687165937436E-04
 0
 0
 9.64066608238387E-03
 2.24131410507988E-02
 1.3017745977284E-04
 3.42431308005303E-02
 0
 0
 3.23051460892813E-02

 0
 0
 0
 0
 0
 0
 0
 0
 7.69631667616081E-04
 0
 4.66462412670815E-04
 0
 4.99496935229519E-05
 0
 3.12975553683276E-02

 0
 0
 0
 0
 0
 0
 0
 6.49296440927937E-05
 0
 1.97549116445645E-04
 1.41528936571713E-03
 0
 0
 5.57903399026459E-05

 0
 0
 0
 0
 0
 0
 0
 6.49296440927937E-05
 0
 1.97549116445645E-04
 1.41528936571713E-03
 0
 0
 5.57903399026459E-05

 0
 0
 0
 0
 1.22121341643847E-04
 0
 5.85943222101778E-05
 0
 5.57528452131335E-04
 0
 1.11314893932808E-03

 0
 0
 0
 0
 0
 0
 0
 0
 1.39382113032834E-04
 0
 9.32924825341631E-04

 0
 0
 0
 0
 0
 0
 0
 0
 2.30283491097725E-04
 0
 1.27217021637495E-04

 0
 0
 0
 0
 1.22121341643847E-04
 0
 5.85943222101778E-05
 0
 1.87862848000776E-04

 0
 0
 0
 0
 0
 0
 0
 0
 0
 0
 5.30070923489563E-05

 0
 0
 0
 7.86431436285947E-05
 0
 0
 5.12700319339056E-05
 0
 0
 0
 3.71049646442694E-05
 4.076391578175E-05
 6.42210345295096E-05
 0
 0
 0
 0
 1.18217283366828E-04

 0
 0
 0
 7.86431436285947E-05
 0
 0
 5.12700319339056E-05
 0
 0
 0
 3.71049646442694E-05
 4.076391578175E-05
 6.42210345295096E-05
 0
 0
 0
 0
 1.18217283366828E-04

 0
 0
 8.99296749941546E-05

 0
 0
 8.99296749941546E-05

 0
 .375
 .585801902911923
 2.94474882253738E-03
 7.39303814413137E-03
 .43854825991518
 9.5728473910878E-03
 .351222996224805
 .22719890433539
 .465489443867374
 .469446711970062
 8.50865214112468E-02
 4.93931112236962E-02
 .254166841011479
 4.64107738829999E-02
 .023525641025641
 .122055153101601
 2.83676011894478E-02

 0
 0
 .536088778575154
 7.86431436285946E-04
 3.61667050252932E-03
 .391812117724783
 4.07962968388363E-03
 4.00012985928819E-02
 .091264983577151
 1.67343219285891E-02
 1.39938723801245E-03
 5.36616187350957E-02
 3.93104088025632E-02
 .107963606101603
 4.58817459228925E-02
 1.15705128205128E-02
 .103494923669028
 2.54349031982322E-02

 0
 0
 .529757729455566
 7.86431436285946E-04
 3.55091285702879E-03
 .385802271807003
 3.91117100752937E-03
 3.21030711721656E-02
 8.61987467730011E-02
 5.70343416835008E-03
 4.13455320321859E-04
 4.83500805087337E-02
 3.75122198357369E-02
 9.56943805180133E-02
 4.57707153633638E-02
 1.09134615384615E-02
 .100863754117544
 2.52894050033192E-02

 0
 0
 3.84899008974982E-03
 0
 0
 4.32623660038787E-03
 0
 7.1376230184864E-03
 2.33919546220321E-03
 .010342652128751
 5.56574469664041E-04
 4.62262804965045E-03
 1.46994812367544E-03
 1.13905277301235E-02
 8.49057219925414E-05
 0
 1.11604878147765E-03
 1.45498194913019E-04

 0
 0
 1.81657943488192E-03
 0
 0
 6.60656820748887E-04
 0
 0
 2.41191656465512E-03
 0
 1.48419858577078E-04
 0
 0
 0
 0
 0
 1.96154028259708E-04

 0
 0
 6.65479594956744E-04
 0
 0
 9.16394944909746E-04
 1.68458676354261E-04
 0
 1.21201837419855E-04
 0
 0
 0
 0
 0
 0
 6.57051282051282E-04
 1.25809135366571E-03

 0
 0
 0
 0
 0
 0
 0
 2.31891586045692E-04
 9.09013780648915E-05
 2.29411877162685E-04
 0
 5.9515317041355E-04
 2.06934444595086E-04
 8.08959928588365E-04
 2.61248375361666E-05
 0
 2.70557280358218E-05

 0
 0
 0
 0
 6.57576455005331E-05
 0
 0
 4.40594013486815E-04
 0
 4.14215889321514E-04
 2.22629787865616E-04

 0
 0
 0
 0
 0
 0
 0
 0
 0
 0
 0
 0
 1.2130639855574E-04

 0
 0
 0
 0
 0
 1.06557551733691E-04
 0
 0
 1.03021561806877E-04
 0
 0
 0
 0
 0
 0
 0
 3.38196600447772E-05

 0
 0
 0
 0
 0
 0
 0
 8.81188026973629E-05
 0
 4.46078650038554E-05
 5.83078015838519E-05
 9.3757006298025E-05
 0
 6.97379248783073E-05

 0
 .375
 0
 0
 0
 0
 0
 4.97685721971264E-02
 2.06043123613754E-04
 .063489737004773
 3.09455405133207E-02
 7.5168660701547E-03
 1.09175758700166E-03
 2.29623740649307E-02
 0
 0
 0
 4.00120036010803E-04

 0
 .375
 0
 0
 0
 0
 0
 4.96943668895918E-02
 2.06043123613754E-04
 6.33877761704785E-02
 3.09455405133207E-02
 7.5168660701547E-03
 1.09175758700166E-03
 2.29623740649307E-02
 0
 0
 0
 4.00120036010803E-04

 0
 0
 0
 0
 0
 0
 0
 7.42053075346214E-05
 0
 1.01960834294527E-04

 0
 0
 3.73567869925718E-02
 3.75739464003286E-04
 7.75000821970569E-04
 3.96394092449332E-02
 2.21193566343422E-03
 .160264912947899
 8.88954876555929E-02
 .265703561619392
 .119212950692803
 6.7994211523959E-03
 1.0132652114656E-03
 9.62987758669587E-02
 0
 9.50320512820513E-03
 1.41636736267527E-02
 1.9551319941437E-04

 0
 0
 4.67634309969604E-04
 0
 2.8181848071657E-05
 7.88525882829316E-04
 6.15240383206867E-04
 .156587112393214
 4.36326614711479E-04
 .260790323916825
 .114749753517021
 6.48146260929825E-03
 1.0132652114656E-03
 .093648734721583
 0
 0
 3.38196600447772E-05
 1.9551319941437E-04

 0
 0
 3.68891526826022E-02
 3.75739464003286E-04
 7.46818973898912E-04
 3.88508833621039E-02
 1.59669528022735E-03
 3.61287091059188E-03
 8.84591610408814E-02
 4.72843369040867E-03
 4.46319717578212E-03
 3.1795854309765E-04
 0
 2.52916207558661E-03
 0
 9.50320512820513E-03
 1.41298539667079E-02

 0
 0
 0
 0
 0
 0
 0
 6.49296440927937E-05
 0
 1.8480401215883E-04
 0
 0
 0
 1.20879069789066E-04

 0
 0
 6.47493659957913E-04
 0
 0
 2.13115103467383E-04
 0
 1.34497119906501E-04
 .023410134897645
 1.01960834294527E-04
 .214132750962079
 3.26111326254E-05
 0
 3.7658479434286E-04
 0
 0
 0
 1.27310920548892E-04

 0
 0
 6.47493659957913E-04
 0
 0
 2.13115103467383E-04
 0
 1.34497119906501E-04
 .023410134897645
 1.01960834294527E-04
 .214132750962079
 3.26111326254E-05
 0
 3.7658479434286E-04

 0
 0
 0
 0
 0
 0
 0
 0
 0
 0
 0
 0
 0
 0
 0
 0
 0
 1.27310920548892E-04

 0
 0
 8.27353009946222E-03
 6.11668894889069E-05
 7.09243176470036E-04
 3.79344884171941E-03
 8.64266252600123E-04
 4.78114072109007E-02
 1.20111020883077E-02
 5.23250256495224E-02
 7.94099250479714E-02
 1.57348714917554E-03
 3.09688099842302E-03
 5.98816314955066E-03
 0
 0
 3.11140872411951E-04
 4.09213673192867E-05

 0
 0
 7.69798017949963E-03
 6.11668894889069E-05
 7.09243176470036E-04
 3.32459561409117E-03
 8.64266252600123E-04
 4.40501256852396E-02
 1.14778140036603E-02
 .047367180081951
 7.67860739766981E-02
 1.51234127550292E-03
 3.09688099842302E-03
 5.47675170044307E-03
 0
 0
 2.43501552322396E-04
 4.09213673192867E-05

 0
 0
 5.75549919962589E-04
 0
 0
 4.68853227628242E-04
 0
 3.76128152566112E-03
 5.33288084647363E-04
 4.95784556757136E-03
 2.62385107127334E-03
 6.1145873672625E-05
 0
 5.11411449107587E-04
 0
 0
 6.76393200895545E-05

 0
 0
 1.42088886490764E-03
 0
 6.48182505648112E-04
 3.62295675894551E-04
 5.85943222101778E-05
 .049411459154616
 9.50222405371665E-03
 6.44711100348579E-02
 2.26075248868298E-02
 4.76530175488657E-03
 1.24160666757051E-03
 1.72206182499501E-02
 1.89405072137208E-04
 0
 2.84085144376128E-04
 9.04816899615339E-04

 0
 0
 0
 0
 4.22727721074856E-04
 0
 5.85943222101778E-05
 3.42643007541114E-02
 2.78764226065667E-04
 4.63794344997228E-02
 1.18735886861662E-02
 4.17014858447302E-03
 1.08462191649838E-03
 1.20274674440121E-02
 1.89405072137208E-04
 0
 1.42042572188064E-04
 8.68442350887084E-04

 0
 0
 1.42088886490764E-03
 0
 0
 3.62295675894551E-04
 0
 1.40572679460898E-02
 7.41755245009514E-03
 1.65686355728606E-02
 1.00660468370668E-02
 4.239447241302E-04
 1.56984751072134E-04
 4.17962629770655E-03
 0
 0
 1.42042572188064E-04
 3.63745487282548E-05

 0
 0
 0
 0
 0
 0
 0
 1.08989045441475E-03
 1.21201837419855E-03
 1.52303996227449E-03
 4.82364540375502E-04
 1.7120844628335E-04
 0
 1.0135245082314E-03

 0
 0
 0
 0
 2.25454784573256E-04
 0
 0
 0
 5.93889003357291E-04
 0
 1.85524823221347E-04

 0
 0
 1.90650910987608E-03
 1.66024414327033E-03
 4.7439444253956E-04
 2.62131577264881E-03
 1.28175079834764E-03
 2.92647181589663E-03
 5.09047717163392E-04
 1.5294125144179E-03
 3.3924539103332E-04
 1.06760695432403E-02
 3.6106492746591E-03
 3.08241627962118E-03
 2.67779584745707E-04
 1.45833333333333E-03
 3.44960532456728E-03
 1.26401556830686E-03

 0
 0
 1.90650910987608E-03
 1.66024414327033E-03
 4.7439444253956E-04
 2.62131577264881E-03
 1.28175079834764E-03
 2.60646142715358E-03
 5.09047717163392E-04
 1.31274574154203E-03
 3.3924539103332E-04
 1.03377290422518E-02
 3.46793586459352E-03
 2.88715008996192E-03
 2.67779584745707E-04
 1.45833333333333E-03
 3.44960532456728E-03
 1.26401556830686E-03

 0
 0
 0
 0
 0
 0
 0
 3.20010388743055E-04
 0
 2.16666772875869E-04
 0
 3.38340500988525E-04
 1.42713410065577E-04
 1.95266189659261E-04

 0
 0
 0
 0
 1.07091022672297E-03
 0
 1.02540063867811E-04
 1.29859288185587E-04
 9.75674791229835E-04
 3.31372711457211E-04
 1.04423971927444E-03
 6.1145873672625E-05
 2.85426820131154E-05
 2.32459749594358E-05
 7.18433032244581E-05

 0
 0
 0
 0
 1.07091022672297E-03
 0
 1.02540063867811E-04
 0
 9.75674791229835E-04
 0
 0
 0
 0
 0
 7.18433032244581E-05

 0
 0
 0
 0
 0
 0
 0
 1.29859288185587E-04
 0
 3.31372711457211E-04
 1.04423971927444E-03
 6.1145873672625E-05
 2.85426820131154E-05
 2.32459749594358E-05

 0
 0
 0
 6.11668894889069E-05
 9.86364682507997E-05
 1.06557551733691E-04
 9.74130606744206E-04
 7.74517897392611E-04
 6.06009187099276E-05
 8.02941570069397E-04
 3.55147518738007E-04
 0
 0
 2.51056529561906E-04
 0
 9.93589743589744E-04
 3.24668736429861E-04

 0
 0
 0
 6.11668894889069E-05
 9.86364682507997E-05
 1.06557551733691E-04
 9.74130606744206E-04
 7.74517897392611E-04
 6.06009187099276E-05
 8.02941570069397E-04
 3.55147518738007E-04
 0
 0
 2.51056529561906E-04
 0
 9.93589743589744E-04
 3.24668736429861E-04

 0
 0
 1.07915609992985E-04
 0
 0
 0
 0
 0
 3.63605512259566E-04
 0
 0
 0
 0
 0
 0
 0
 2.70557280358218E-05

 0
 0
 1.07915609992985E-04
 0
 0
 0
 0
 0
 3.63605512259566E-04
 0
 0
 0
 0
 0
 0
 0
 2.70557280358218E-05

 .1875
 0
 6.72494109606288E-02
 .76776679686476
 .606618976717097
 7.36525797583275E-02
 .459753024931884
 .402396831433369
 .164495133746227
 .351987917641136
 .318047854802972
 .315655381855981
 .391891024040074
 .24399440236923
 .229238918170478
 .21636217948718
 6.50554980621335E-02
 .243695836023534

 0
 0
 9.89226424935701E-04
 .511058099806887
 6.90924975223458E-03
 6.81968331095625E-04
 1.66993818299006E-03
 .270181524733557
 1.82408765316882E-03
 .246675120919177
 2.52843830504522E-03
 3.11843955730387E-02
 1.58625955287888E-02
 .139080668182304
 2.82213557484439E-02
 0
 1.21750776161198E-04
 2.93860885538389E-02

 0
 0
 2.69789024982464E-04
 .510743527232373
 4.95530828593303E-03
 0
 6.37213254035684E-04
 0
 0
 0
 0
 2.8534741047225E-05
 7.13567050327884E-05
 0
 5.22496750723331E-04
 0
 0
 5.00150045013504E-05

 0
 0
 7.19437399953237E-04
 3.14572574514379E-04
 1.51712282119087E-03
 6.81968331095625E-04
 1.03272492895438E-03
 .270074854603976
 1.69682572387797E-03
 .246407473729154
 2.41712341111241E-03
 3.09561176446609E-02
 1.47637022712839E-02
 .138727329362921
 1.10377438590304E-03
 0
 1.21750776161198E-04
 1.09123646184765E-03

 0
 0
 0
 0
 2.8181848071657E-05
 0
 0
 0
 0
 0
 0
 3.26111326254E-05
 1.02753655247215E-03
 0
 .026510178889825
 0
 0
 2.82448370874899E-02

 0
 0
 0
 0
 3.47576126217104E-04
 0
 0
 0
 0
 7.0098073577487E-05
 1.11314893932808E-04
 1.67132054705175E-04
 0
 5.57903399026459E-05

 0
 0
 0
 0
 0
 0
 0
 0
 6.06009187099276E-05
 4.46078650038554E-05
 0
 0
 0
 2.04564579643035E-04
 1.30624187680833E-05

 0
 0
 0
 0
 0
 0
 0
 1.06670129581018E-04
 0
 1.5294125144179E-04
 0
 0
 0
 9.29838998377431E-05

 0
 0
 0
 0
 6.10606708219236E-05
 0
 0
 0
 6.66610105809204E-05
 0
 0
 0
 0
 0
 7.18433032244581E-05

 0
 0
 3.90294789474631E-03
 2.10326718571142E-02
 .506625082784179
 2.04590499328687E-03
 5.39800193361263E-03
 0
 8.05992218842038E-04
 0
 1.96126241691138E-04
 2.26647371746529E-03
 8.24883510179034E-03
 0
 .144489945203153
 3.50961538461538E-03
 2.9761300839404E-03
 2.89996089736011E-02

 0
 0
 1.31297325491466E-03
 .020875385569857
 .50239780557343
 2.04590499328687E-03
 4.59233000322269E-03
 0
 7.51451392003103E-04
 0
 1.27217021637495E-04
 1.76507755334977E-03
 2.14783682148693E-03
 0
 .085003690133302
 3.17307692307692E-03
 2.45530731925083E-03
 5.81538097792974E-03

 0
 0
 2.58997463983165E-03
 1.57286287257189E-04
 8.17273594078054E-04
 0
 8.05671930389945E-04
 0
 5.45408268389349E-05
 0
 6.89092200536432E-05
 5.01396164115525E-04
 6.10099828030341E-03
 0
 5.94862550698513E-02
 3.36538461538462E-04
 5.20822764689569E-04
 2.31842279956714E-02

 0
 0
 0
 0
 3.4100036166705E-03

 .1875
 0
 2.67990431482581E-03
 .153957060843578
 2.86703334382324E-02
 1.87541291051297E-03
 .331988105352591
 4.01172443859047E-03
 3.46637255020786E-03
 1.54215761870472E-03
 9.64729080751005E-04
 4.47628559199397E-02
 1.58982738813052E-02
 2.40781808629835E-02
 2.94557543220278E-03
 .107708333333333
 5.8440372557375E-03
 6.09728373057371E-03

 .1875
 0
 1.33095918991349E-03
 .141216871575746
 1.11083451149115E-02
 7.24591351789101E-04
 .312315061670524
 2.91255832073389E-03
 1.27261929290848E-04
 9.24020060794148E-04
 1.85524823221347E-04
 3.90355257526038E-02
 1.44568684396429E-02
 1.99636432951634E-02
 1.14296164220729E-03
 9.66826923076923E-02
 1.65716334219408E-03
 1.20036010803241E-03

 0
 0
 9.17282684940377E-04
 1.24693073286672E-02
 1.70969878301386E-02
 8.52460413869531E-04
 1.87721559780857E-02
 0
 2.12103215484747E-04
 0
 0
 0
 0
 0
 0
 .011025641025641
 4.13276245747178E-03
 3.63745487282548E-05

 0
 0
 0
 0
 0
 0
 0
 6.12193787160627E-04
 1.21201837419855E-04
 4.2696099360833E-04
 1.32517730872391E-04
 5.44198275686362E-03
 1.19879264455085E-03
 3.70540840853406E-03
 7.57620288548831E-04
 0
 0
 4.36949266598161E-03

 0
 0
 4.31662439971942E-04
 1.39810033117502E-04
 3.38182176859885E-04
 2.98361144854336E-04
 4.46781706852606E-04
 3.61750874231279E-04
 2.79976244439866E-03
 1.91176564302237E-04
 2.9153900791926E-04
 2.64965452581375E-04
 2.42612797111481E-04
 5.57903399026459E-05
 9.20900523149872E-04
 0
 5.41114560716436E-05
 3.63745487282548E-04

 0
 0
 0
 1.31071906047658E-04
 1.26818316322457E-04
 0
 4.54105997128878E-04
 1.25221456464674E-04
 2.06043123613754E-04
 0
 2.22629787865616E-04
 2.0381957890875E-05
 0
 3.53338819383424E-04

 0
 0
 0
 0
 0
 0
 0
 0
 0
 0
 1.32517730872391E-04

 0
 0
 0
 0
 0
 0
 0
 0
 0
 0
 0
 0
 0
 0
 1.24092978296791E-04
 0
 0
 1.27310920548892E-04

 0
 0
 6.04327415960719E-03
 .013823717024493
 2.56454817452079E-03
 .008631161690429
 1.05176808367269E-02
 3.20474171915146E-03
 5.96313040105688E-03
 1.99460882088668E-03
 1.43119149342182E-04
 .115594235982308
 .1418928079577
 1.41010084103937E-02
 2.84107608205811E-03
 5.22435897435897E-03
 6.08077487605095E-03
 9.38690698118526E-02

 0
 0
 6.04327415960719E-03
 .013823717024493
 2.56454817452079E-03
 .008631161690429
 1.05176808367269E-02
 3.20474171915146E-03
 5.96313040105688E-03
 1.99460882088668E-03
 1.43119149342182E-04
 .115594235982308
 .1418928079577
 1.41010084103937E-02
 2.84107608205811E-03
 5.22435897435897E-03
 6.08077487605095E-03
 9.38690698118526E-02

 0
 0
 5.07203366967032E-03
 0
 1.59697139072723E-04
 7.22460200754427E-03
 1.02540063867811E-04
 4.11097403741802E-02
 .119638333717139
 .031728937122028
 .271290298641958
 1.70352404051933E-02
 6.23657601986571E-03
 3.69053098456003E-02
 1.1756176891275E-04
 0
 1.8059698463911E-03
 1.29584329844408E-03

 0
 0
 5.39578049964927E-05
 0
 0
 0
 0
 4.69812353328572E-03
 1.17868786890809E-02
 3.98284508962995E-03
 .120400309561419
 4.03562766239325E-04
 1.2130639855574E-04
 1.66906100208749E-03
 0
 0
 0
 2.09153655187465E-04

 0
 0
 4.7123149696937E-03
 0
 1.03333442929409E-04
 7.22460200754427E-03
 5.12700319339056E-05
 1.03423647376379E-03
 .105197134788563
 1.57402037942175E-03
 4.44729504807743E-03
 5.87408026415017E-03
 3.31095111352138E-03
 3.99365849803107E-03
 1.1756176891275E-04
 0
 1.8059698463911E-03
 8.59348713705021E-04

 0
 0
 1.61873414989478E-04
 0
 0
 0
 0
 2.70385589329277E-03
 4.72687165937436E-04
 3.30735456242871E-03
 9.28472229584318E-02
 4.03562766239325E-04
 1.07035057549183E-04
 1.92476672664128E-03

 0
 0
 0
 0
 5.63636961433141E-05
 0
 5.12700319339056E-05
 3.21169846673283E-02
 2.42403674839711E-04
 2.16921674961605E-02
 5.21165731974938E-02
 1.02276664696411E-02
 2.54743436967055E-03
 2.87878153897653E-02
 0
 0
 0
 2.27340929551593E-04

 0
 0
 0
 0
 0
 0
 0
 0
 1.63016471329705E-03
 1.14705938581342E-04
 3.44546100268216E-04

 0
 0
 1.43887479990647E-04
 0
 0
 0
 0
 5.56539806509661E-04
 2.54523858581696E-04
 1.00686323865845E-03
 1.13435177626766E-03
 9.78333978762E-05
 1.49849080568856E-04
 4.92814669140038E-04

 0
 0
 0
 0
 0
 0
 0
 0
 5.45408268389349E-05

 0
 0
 0
 0
 0
 0
 0
 0
 0
 5.09804171472633E-05
 0
 2.8534741047225E-05
 0
 3.71935599350972E-05

 0
 0
 2.08636845986439E-03
 8.01286252304681E-03
 1.09909207479462E-03
 1.53442874496516E-03
 3.14944481879706E-04
 2.14731608678311E-02
 3.95723999175827E-03
 8.35441586000777E-03
 1.96126241691138E-04
 1.41328496015327E-02
 .10781284563404
 2.17489341720481E-02
 8.11829326436376E-03
 2.43589743589744E-03
 1.75862232232842E-03
 5.50619731373958E-03

 0
 0
 2.08636845986439E-03
 8.01286252304681E-03
 1.09909207479462E-03
 1.53442874496516E-03
 3.14944481879706E-04
 2.14731608678311E-02
 3.95723999175827E-03
 8.35441586000777E-03
 1.96126241691138E-04
 1.41328496015327E-02
 .10781284563404
 2.17489341720481E-02
 8.11829326436376E-03
 2.43589743589744E-03
 1.75862232232842E-03
 5.50619731373958E-03

 0
 0
 1.15289843342506E-02
 6.59728593773211E-03
 4.04691338308995E-02
 8.43935809730836E-03
 3.44754343304134E-02
 6.02918123718799E-05
 6.18129370841262E-04
 5.73529692906712E-05
 1.96126241691138E-04
 8.77035648044351E-02
 8.65556832047723E-02
 7.9966153860459E-04
 2.20101756242203E-03
 4.60256410256411E-02
 7.85292506239727E-03
 4.62911600752954E-02

 0
 0
 5.48571017464343E-03
 3.28553577826129E-03
 5.74440003193943E-03
 7.65083221447904E-03
 2.60891219640817E-02
 6.02918123718799E-05
 4.54506890324457E-04
 0
 0
 8.76587244970752E-02
 8.63986984537002E-02
 7.29923613726283E-04
 2.20101756242203E-03
 4.37019230769231E-02
 6.52043045663305E-03
 4.52544854365401E-02

 0
 0
 5.21592114966097E-04
 2.36803243592768E-03
 2.36680554055133E-02
 0
 5.91070225295169E-03
 0
 0
 0
 0
 4.4840307359925E-05
 0
 0
 0
 9.93589743589744E-04

 0
 0
 1.61873414989478E-04
 6.99050165587508E-04
 1.02347078246901E-02
 2.55738124160859E-04
 1.68458676354261E-03
 0
 0
 5.73529692906712E-05
 0
 0
 1.07035057549183E-04
 6.97379248783073E-05
 0
 7.53205128205128E-04
 3.58488396474639E-04
 7.95693253430575E-04

 0
 0
 4.85620244968435E-03
 0
 1.9257596182299E-04

 0
 0
 5.03606179967266E-04
 1.31071906047658E-04
 2.44242683287694E-04
 5.32787758668457E-04
 5.85943222101778E-04
 0
 1.33322021161841E-04
 0
 0
 0
 0
 0
 0
 4.6474358974359E-04
 8.79311161164208E-04

 0
 0
 0
 1.1359565190797E-04
 3.8515192364598E-04
 0
 2.05080127735622E-04
 0
 0
 0
 0
 0
 4.99496935229519E-05
 0
 0
 1.12179487179487E-04
 9.46950481253762E-05
 2.40981385324688E-04

 0
 0
 0
 0
 0
 0
 0
 0
 3.03004593549638E-05
 0
 1.96126241691138E-04

 0
 0
 3.83100415475098E-03
 2.60833093034839E-02
 1.19303156836681E-03
 7.67214372482578E-03
 4.25321536343128E-02
 0
 4.07238173730714E-03
 0
 3.60448227972903E-04
 1.49603570919022E-03
 7.89918724712968E-03
 5.20709839091361E-04
 .017255455192638
 2.38141025641026E-02
 1.11672517467854E-02
 .024689224949303

 0
 0
 3.83100415475098E-03
 2.60833093034839E-02
 1.19303156836681E-03
 7.67214372482578E-03
 4.25321536343128E-02
 0
 4.07238173730714E-03
 0
 3.60448227972903E-04
 1.49603570919022E-03
 7.89918724712968E-03
 5.20709839091361E-04
 .017255455192638
 2.38141025641026E-02
 1.11672517467854E-02
 .024689224949303

 0
 0
 3.05760894980126E-04
 0
 0
 0
 0
 .036277119720988
 5.16925836595683E-03
 .042039726490062
 1.17887773384079E-02
 4.89166989381E-05
 7.13567050327884E-05
 1.00887531323951E-03

 0
 0
 3.05760894980126E-04
 0
 0
 0
 0
 .036277119720988
 5.16925836595683E-03
 .042039726490062
 1.17887773384079E-02
 4.89166989381E-05
 7.13567050327884E-05
 1.00887531323951E-03

 0
 0
 2.2122700048562E-03
 0
 0
 1.53442874496516E-03
 3.66214513813611E-05
 1.58613844855253E-03
 5.90252948234695E-03
 1.30637318939862E-03
 2.55335163844922E-02
 0
 0
 5.57903399026459E-04
 0
 0
 4.05835920537327E-05

 0
 0
 2.2122700048562E-03
 0
 0
 1.53442874496516E-03
 3.66214513813611E-05
 1.58613844855253E-03
 5.90252948234695E-03
 1.30637318939862E-03
 2.55335163844922E-02
 0
 0
 5.57903399026459E-04
 0
 0
 4.05835920537327E-05

 0
 0
 1.80938506088239E-02
 4.72732674478552E-03
 4.8378839189678E-04
 2.39754491400805E-02
 1.19385931503238E-02
 2.25908783125713E-02
 5.87828911486298E-03
 1.60078509842407E-02
 2.89948795148791E-03
 6.56299044086175E-04
 5.13768276236077E-04
 2.37108944586245E-03
 2.19122074834598E-02
 1.02083333333333E-02
 2.07043958794127E-02
 1.32312420999027E-03

 0
 0
 3.27344016978723E-03
 1.50295785601314E-03
 7.04546201791426E-05
 1.51311723461842E-03
 4.83403158233967E-04
 1.53233960058993E-02
 2.99368538427043E-03
 3.70882534746341E-03
 0
 4.8101420622465E-04
 1.99798774091808E-04
 1.16694794296368E-03
 2.17162712019385E-02
 1.21794871794872E-03
 1.05517339339705E-03
 1.30948375421717E-03

 0
 0
 1.42628464540729E-02
 3.09329698272472E-03
 3.75757974288761E-04
 .021332821857085
 1.09278410921982E-02
 0
 2.06649132800853E-03
 0
 0
 0
 0
 0
 0
 8.83012820512821E-03
 1.92095669054335E-02

 0
 0
 0
 0
 0
 0
 0
 3.93751913105585E-03
 0
 1.04701031716192E-02
 2.60794894356865E-03
 1.141389641889E-04
 3.13969502144269E-04
 6.41588908880427E-04
 7.83745126084997E-05
 0
 0
 1.36404557730956E-05

 0
 0
 0
 0
 0
 0
 0
 2.66211540780454E-03
 6.06009187099276E-05
 1.34460850225907E-03
 2.5443404327499E-04
 6.1145873672625E-05
 0
 4.55621109204941E-04

 0
 0
 4.13676504973111E-04
 1.31071906047658E-04
 0
 1.12951004837713E-03
 1.318372249729E-04
 0
 5.81768819615305E-04
 0
 0
 0
 0
 0
 0
 1.6025641025641E-04
 3.8554412451046E-04

 0
 0
 1.43887479990647E-04
 0
 0
 0
 0
 6.67847767811593E-04
 1.7574266425879E-04
 4.84313962899001E-04
 3.71049646442694E-05
 0
 0
 1.06931484813405E-04
 1.1756176891275E-04
 0
 5.41114560716436E-05

 0
 0
 0
 0
 3.75757974288761E-05
 0
 3.955116749187E-04

 0
 0
 1.24102951491933E-03
 1.91015457746787E-02
 1.2822740872604E-03
 3.26066108305096E-03
 1.65528960243752E-02
 1.72063556845903E-03
 2.42403674839711E-04
 2.02647158160372E-03
 4.71763121905711E-04
 5.01396164115525E-04
 2.42612797111481E-04
 2.5710048305136E-03
 1.37155397064874E-04
 1.58333333333333E-02
 4.24774930162402E-03
 7.18397337383033E-04

 0
 0
 1.24102951491933E-03
 1.91015457746787E-02
 1.2822740872604E-03
 3.26066108305096E-03
 1.65528960243752E-02
 1.72063556845903E-03
 2.42403674839711E-04
 2.02647158160372E-03
 4.71763121905711E-04
 5.01396164115525E-04
 2.42612797111481E-04
 2.5710048305136E-03
 1.37155397064874E-04
 1.58333333333333E-02
 4.24774930162402E-03
 7.18397337383033E-04

 0
 0
 7.84186765949028E-03
 1.16217090028923E-03
 1.52369858574092E-02
 5.54099269015195E-03
 1.0034277678493E-03
 0
 4.02996109421019E-03
 0
 2.17329078630721E-04
 0
 0
 0
 4.11466191194624E-04
 1.76282051282051E-04
 1.77891411835528E-03
 1.04576827593733E-04

 0
 0
 5.7015413946294E-03
 6.20407021958913E-04
 7.74531124502708E-03
 3.47377618651834E-03
 4.54105997128878E-04
 0
 1.55138351897415E-03
 0
 0
 0
 0
 0
 0
 0
 7.84616113038832E-04

 0
 0
 2.14032626486088E-03
 5.41763878330319E-04
 7.37425024541693E-03
 2.06721650363361E-03
 5.49321770720417E-04
 0
 2.47857757523604E-03
 0
 2.17329078630721E-04
 0
 0
 0
 3.72278934890374E-04
 1.76282051282051E-04
 9.94298005316451E-04
 1.04576827593733E-04

 0
 0
 0
 0
 1.17424366965238E-04

 0
 0
 0
 0
 0
 0
 0
 0
 0
 0
 0
 0
 0
 0
 3.91872563042499E-05

 0
 0
 3.59718699976618E-04
 1.10974213787017E-03
 8.21970568756664E-04
 2.98361144854336E-04
 1.15723786365101E-03
 1.43772783348329E-04
 2.36343582968718E-04
 2.10294220732461E-04
 3.02140426389051E-04
 6.52222652508E-05
 0
 1.02282289821517E-04
 0
 5.76923076923077E-04
 2.57029416340307E-04
 2.01424063582711E-03

 0
 0
 3.59718699976618E-04
 1.10974213787017E-03
 8.21970568756664E-04
 2.98361144854336E-04
 1.15723786365101E-03
 1.43772783348329E-04
 2.36343582968718E-04
 2.10294220732461E-04
 3.02140426389051E-04
 6.52222652508E-05
 0
 1.02282289821517E-04
 0
 5.76923076923077E-04
 2.57029416340307E-04
 2.01424063582711E-03

 0
 0
 8.09367074947391E-04
 2.53405685025472E-04
 6.95152252434207E-04
 7.4590286213584E-04
 5.78618931825506E-04
 0
 1.99983031742761E-04
 0
 0
 0
 0
 0
 2.93904422281874E-04
 4.32692307692308E-04
 2.02917960268663E-04
 1.96422563132576E-03

 0
 0
 8.09367074947391E-04
 2.53405685025472E-04
 6.95152252434207E-04
 7.4590286213584E-04
 5.78618931825506E-04
 0
 1.99983031742761E-04
 0
 0
 0
 0
 0
 2.93904422281874E-04
 4.32692307692308E-04
 2.02917960268663E-04
 1.96422563132576E-03

 0
 0
 0
 6.37883276098601E-04
 3.66364024931542E-04
 0
 1.0546977997832E-03
 0
 0
 0
 0
 1.67132054705175E-04
 2.92562490634432E-04
 0
 0
 1.28205128205128E-04
 4.05835920537327E-05
 3.9102639882874E-04

 0
 0
 0
 6.37883276098601E-04
 3.66364024931542E-04
 0
 1.0546977997832E-03
 0
 0
 0
 0
 1.67132054705175E-04
 2.92562490634432E-04
 0
 0
 1.28205128205128E-04
 4.05835920537327E-05
 3.9102639882874E-04

 0
 0
 0
 0
 0
 0
 0
 0
 8.84773413164943E-04
 4.46078650038554E-05
 2.49133334040095E-04
 0
 0
 7.43871198701945E-05
 3.26560469202082E-05
 0
 0
 5.45618230923823E-05

 0
 0
 0
 0
 0
 0
 0
 0
 7.63571575745088E-04
 4.46078650038554E-05
 2.49133334040095E-04
 0
 0
 7.43871198701945E-05
 3.26560469202082E-05
 0
 0
 5.45618230923823E-05

 0
 0
 0
 0
 0
 0
 0
 0
 1.21201837419855E-04

 0
 0
 7.19437399953237E-05
 0
 0
 0
 0
 3.71026537673107E-05
 9.69614699358842E-05
 0
 2.65035461744781E-05
 2.8534741047225E-05
 2.92562490634432E-04
 4.18427549269844E-05
 2.61248375361666E-04
 0
 0
 5.95633235425173E-04

 0
 0
 7.19437399953237E-05
 0
 0
 0
 0
 3.71026537673107E-05
 9.69614699358842E-05
 0
 2.65035461744781E-05
 2.8534741047225E-05
 2.92562490634432E-04
 4.18427549269844E-05
 2.61248375361666E-04
 0
 0
 5.95633235425173E-04

 0
 0
 1.79859349988309E-04
 2.62143812095315E-05
 4.22727721074855E-05
 1.91803593120644E-04
 0
 0
 8.7871332129395E-04
 0
 2.12028369395825E-05
 1.2229174734525E-05
 0
 0
 0
 0
 2.70557280358218E-05
 1.81872743641274E-05

 0
 0
 1.79859349988309E-04
 0
 1.8787898714438E-05
 1.91803593120644E-04
 0
 0
 5.02987625292399E-04
 0
 2.12028369395825E-05
 0
 0
 0
 0
 0
 2.70557280358218E-05

 0
 0
 0
 0
 0
 0
 0
 0
 3.27244961033609E-04

 0
 0
 0
 0
 2.34848733930475E-05
 0
 0
 0
 4.84807349679421E-05
 0
 0
 0
 0
 0
 0
 0
 0
 1.81872743641274E-05

 0
 0
 0
 2.62143812095315E-05
 0
 0
 0
 0
 0
 0
 0
 1.2229174734525E-05

 0
 0
 0
 0
 0
 0
 0
 0
 2.54523858581696E-04
 0
 4.77063831140607E-04
 0
 0
 0
 0
 0
 1.35278640179109E-05

 0
 0
 0
 0
 0
 0
 0
 0
 2.54523858581696E-04
 0
 4.77063831140607E-04
 0
 0
 0
 0
 0
 1.35278640179109E-05

 0
 0
 0
 1.83500668466721E-04
 0
 0
 4.32133126300062E-04
 0
 9.69614699358842E-05
 0
 1.21916312402599E-04
 0
 7.13567050327884E-05
 0
 0
 2.88461538461538E-04
 1.35278640179109E-04

 0
 0
 0
 1.83500668466721E-04
 0
 0
 4.32133126300062E-04
 0
 9.69614699358842E-05
 0
 1.21916312402599E-04
 0
 7.13567050327884E-05
 0
 0
 2.88461538461538E-04
 1.35278640179109E-04

 0
 0
 0
 0
 0
 0
 0
 0
 0
 0
 0
 0
 0
 3.25443649432101E-05
 0
 0
 0
 3.77385943055644E-04

 0
 0
 0
 0
 0
 0
 0
 0
 0
 0
 0
 0
 0
 3.25443649432101E-05
 0
 0
 0
 3.77385943055644E-04

 0
 0
 0
 0
 0
 0
 0
 0
 2.06043123613754E-04
 0
 6.36085108187475E-05

 0
 0
 0
 0
 0
 0
 0
 0
 2.06043123613754E-04
 0
 6.36085108187475E-05

 0
 0
 0
 0
 0
 0
 0
 0
 7.27211024519132E-05

 0
 0
 0
 0
 0
 0
 0
 0
 7.27211024519132E-05

 0
 0
 4.37058220471591E-03
 1.03983712131142E-03
 1.31515291001066E-04
 2.77049634507598E-03
 1.55274953856971E-03
 3.23535140850949E-02
 3.96936017550026E-03
 2.52990320093294E-03
 0
 .17321810733139
 7.78073511677525E-02
 6.90544932144999E-02
 1.90711314014016E-03
 1.28205128205128E-03
 1.67745513822095E-03
 2.44164158338411E-02

 0
 0
 4.06482130973579E-03
 1.1359565190797E-04
 6.10606708219236E-05
 2.40820066918143E-03
 3.36917352708523E-04
 3.23535140850949E-02
 3.87239870556438E-03
 2.52990320093294E-03
 0
 .17321810733139
 7.78073511677525E-02
 6.90544932144999E-02
 1.90711314014016E-03
 0
 9.26658685226896E-04
 2.44164158338411E-02

 0
 0
 3.4892713897732E-03
 1.1359565190797E-04
 6.10606708219236E-05
 2.02459348294014E-03
 3.36917352708523E-04
 2.99603929171034E-02
 2.27859454349328E-03
 1.87990288230533E-03
 0
 .162566496137619
 7.23485632327442E-02
 6.50515363264851E-02
 1.80261378999549E-03
 0
 6.35809608841812E-04
 .023693471677867

 0
 0
 5.75549919962589E-04
 0
 0
 3.83607186241289E-04
 0
 2.39312116799154E-03
 1.5938041620711E-03
 6.50000318627607E-04
 0
 1.06516111937713E-02
 5.45878793500831E-03
 4.00295688801484E-03
 1.04499350144666E-04
 0
 2.90849076385084E-04
 7.22944155974065E-04

 0
 0
 3.05760894980126E-04
 9.26241469403448E-04
 7.04546201791426E-05
 3.62295675894551E-04
 1.21583218586119E-03
 0
 9.69614699358842E-05
 0
 0
 0
 0
 0
 0
 1.28205128205128E-03
 7.50796452994054E-04

 0
 0
 3.05760894980126E-04
 9.26241469403448E-04
 7.04546201791426E-05
 3.62295675894551E-04
 1.21583218586119E-03
 0
 9.69614699358842E-05
 0
 0
 0
 0
 0
 0
 1.28205128205128E-03
 7.50796452994054E-04

 0
 0
 .033831543732801
 8.08276753960556E-03
 5.70212725983194E-03
 2.27606930503165E-02
 4.24222892801688E-02
 2.7826990325483E-05
 3.50455112899512E-02
 6.37255214340791E-05
 9.96533336160378E-04
 6.16676517946314E-02
 4.16223660456255E-02
 7.99661538604591E-04
 2.16836151550183E-02
 7.50641025641026E-02
 2.01632813186962E-02
 3.49423008720798E-02

 0
 0
 .033831543732801
 8.08276753960556E-03
 5.70212725983194E-03
 2.27606930503165E-02
 4.24222892801688E-02
 2.7826990325483E-05
 3.50455112899512E-02
 6.37255214340791E-05
 9.96533336160378E-04
 6.16676517946314E-02
 4.16223660456255E-02
 7.99661538604591E-04
 2.16836151550183E-02
 7.50641025641026E-02
 2.01632813186962E-02
 3.49423008720798E-02

 0
 0
 .033831543732801
 8.08276753960556E-03
 5.70212725983194E-03
 2.27606930503165E-02
 4.24222892801688E-02
 2.7826990325483E-05
 3.50455112899512E-02
 6.37255214340791E-05
 9.96533336160378E-04
 6.16676517946314E-02
 4.16223660456255E-02
 7.99661538604591E-04
 2.16836151550183E-02
 7.50641025641026E-02
 2.01632813186962E-02
 3.49423008720798E-02

 0
 0
 1.61873414989478E-04
 1.31071906047658E-04
 2.34848733930475E-04
 2.34426613814121E-04
 5.85943222101778E-04
 0
 6.06009187099276E-05
 0
 0
 1.8751401259605E-03
 2.48321333514104E-02
 1.85967799675486E-05
 1.39767880818491E-03
 4.32692307692308E-04
 4.87003104644792E-04
 6.22959615157274E-02

 0
 0
 1.61873414989478E-04
 1.31071906047658E-04
 2.34848733930475E-04
 2.34426613814121E-04
 5.85943222101778E-04
 0
 6.06009187099276E-05
 0
 0
 1.8751401259605E-03
 2.48321333514104E-02
 1.85967799675486E-05
 1.39767880818491E-03
 4.32692307692308E-04
 4.87003104644792E-04
 6.22959615157274E-02

 0
 0
 1.61873414989478E-04
 1.31071906047658E-04
 2.34848733930475E-04
 2.34426613814121E-04
 5.85943222101778E-04
 0
 6.06009187099276E-05
 0
 0
 1.8751401259605E-03
 2.48321333514104E-02
 1.85967799675486E-05
 1.39767880818491E-03
 4.32692307692308E-04
 4.87003104644792E-04
 6.22959615157274E-02

 0
 0
 7.19437399953237E-05
 4.19430099352505E-04
 1.70030483365664E-03
 5.11476248321718E-04
 3.14944481879706E-04
 0
 1.7574266425879E-04
 0
 0
 5.2993090516275E-05
 7.84923755360672E-04
 0
 2.41654747209541E-02
 3.68589743589744E-04
 3.58488396474639E-04
 1.23218783816963E-03

 0
 0
 7.19437399953237E-05
 4.19430099352505E-04
 1.70030483365664E-03
 5.11476248321718E-04
 3.14944481879706E-04
 0
 1.7574266425879E-04
 0
 0
 5.2993090516275E-05
 7.84923755360672E-04
 0
 2.41654747209541E-02
 3.68589743589744E-04
 3.58488396474639E-04
 1.23218783816963E-03

 0
 0
 7.19437399953237E-05
 5.24287624190631E-05
 6.10606708219236E-05
 1.06557551733691E-04
 2.12404418011895E-04
 0
 9.09013780648915E-05
 0
 0
 5.2993090516275E-05
 3.13969502144269E-04
 0
 1.41727243633704E-02
 3.68589743589744E-04
 1.35278640179109E-04
 5.77445961061046E-04

 0
 0
 0
 3.67001336933442E-04
 1.63924416283472E-03
 4.04918696588027E-04
 1.02540063867811E-04
 0
 8.48412861938987E-05
 0
 0
 0
 4.70954253216403E-04
 0
 9.99275035758371E-03
 0
 2.2320975629553E-04
 6.54741877108587E-04

 0
 0
 0
 2.18453176746096E-04
 0
 0
 1.46485805525445E-04
 0
 0
 0
 0
 0
 0
 0
 1.18737386601877E-02
 0
 0
 4.09213673192867E-05

 0
 0
 0
 2.18453176746096E-04
 0
 0
 1.46485805525445E-04
 0
 0
 0
 0
 0
 0
 0
 1.18737386601877E-02
 0
 0
 4.09213673192867E-05

 0
 0
 0
 2.18453176746096E-04
 0
 0
 1.46485805525445E-04
 0
 0
 0
 0
 0
 0
 0
 1.18737386601877E-02
 0
 0
 4.09213673192867E-05

 0
 0
 0
 1.44179096652424E-03
 4.18030746396246E-04
 0
 4.55570855184133E-03

 0
 0
 0
 1.44179096652424E-03
 4.18030746396246E-04
 0
 4.55570855184133E-03

 0
 0
 0
 1.44179096652424E-03
 4.18030746396246E-04
 0
 4.55570855184133E-03

 0
 0
 3.23746829978956E-04
 3.4078695572391E-04
 2.11363860537428E-04
 2.77049634507598E-04
 1.12794070254592E-03
 0
 1.51502296774819E-04
 0
 4.39958866496337E-04
 2.48659886268675E-04
 7.9919509636723E-04
 0
 2.67779584745707E-04
 1.02564102564103E-03
 6.62865336877634E-04
 4.18307310374931E-03

 0
 0
 3.23746829978956E-04
 3.4078695572391E-04
 2.11363860537428E-04
 2.77049634507598E-04
 1.12794070254592E-03
 0
 1.51502296774819E-04
 0
 4.39958866496337E-04
 2.48659886268675E-04
 7.9919509636723E-04
 0
 2.67779584745707E-04
 1.02564102564103E-03
 6.62865336877634E-04
 4.18307310374931E-03

 0
 0
 3.23746829978956E-04
 3.4078695572391E-04
 2.11363860537428E-04
 2.77049634507598E-04
 1.12794070254592E-03
 0
 1.51502296774819E-04
 0
 4.39958866496337E-04
 2.48659886268675E-04
 7.9919509636723E-04
 0
 2.67779584745707E-04
 1.02564102564103E-03
 6.62865336877634E-04
 4.18307310374931E-03

 0
 0
 0
 0
 1.64394113751333E-04
 0
 5.12700319339056E-05
 0
 1.81802756129783E-05
 0
 6.89092200536432E-05
 0
 0
 0
 0
 3.91025641025641E-03

 0
 0
 0
 0
 1.64394113751333E-04
 0
 5.12700319339056E-05
 0
 1.81802756129783E-05
 0
 6.89092200536432E-05
 0
 0
 0
 0
 3.91025641025641E-03

 0
 0
 0
 0
 1.64394113751333E-04
 0
 5.12700319339056E-05
 0
 1.81802756129783E-05
 0
 6.89092200536432E-05
 0
 0
 0
 0
 3.91025641025641E-03

 0
 0
 2.80580585981763E-03
 4.89335115911256E-04
 9.86364682507997E-04
 4.26230206934765E-03
 9.00887703981484E-04
 1.02032297860104E-04
 8.5447295380998E-04
 5.09804171472633E-05
 1.27217021637495E-04
 5.2993090516275E-05
 3.28240843150827E-04
 2.37108944586245E-04
 3.91872563042499E-05
 7.53205128205128E-04
 4.34920828175835E-03
 7.72959160475415E-05

 0
 0
 2.80580585981763E-03
 4.89335115911256E-04
 9.86364682507997E-04
 4.26230206934765E-03
 9.00887703981484E-04
 1.02032297860104E-04
 8.5447295380998E-04
 5.09804171472633E-05
 1.27217021637495E-04
 5.2993090516275E-05
 3.28240843150827E-04
 2.37108944586245E-04
 3.91872563042499E-05
 7.53205128205128E-04
 4.34920828175835E-03
 7.72959160475415E-05

 0
 0
 1.88852317487725E-03
 1.66024414327033E-04
 8.03182670042226E-04
 3.15410353131726E-03
 5.78618931825506E-04
 9.27566344182768E-06
 6.18129370841262E-04
 0
 0
 0
 0
 0
 0
 6.41025641025641E-04
 3.61193969278221E-03

 0
 0
 9.17282684940377E-04
 3.05834447444535E-04
 1.83182012465771E-04
 1.10819853803039E-03
 3.22268772155978E-04
 0
 1.7574266425879E-04
 0
 0
 0
 0
 6.0439534894533E-05
 0
 1.12179487179487E-04
 7.16976792949277E-04

 0
 0
 0
 1.74762541396877E-05
 0
 0
 0
 9.27566344182768E-05
 6.06009187099276E-05
 5.09804171472633E-05
 1.27217021637495E-04
 5.2993090516275E-05
 3.28240843150827E-04
 1.76669409691712E-04
 3.91872563042499E-05
 0
 2.02917960268663E-05
 7.72959160475415E-05

 0
 0
 0
 7.86431436285947E-05
 2.35788128866197E-03
 0
 3.00295901327161E-04
 0
 2.42403674839711E-05
 0
 0
 0
 0
 2.78951699513229E-05
 0
 0
 9.46950481253762E-05

 0
 0
 0
 7.86431436285947E-05
 2.35788128866197E-03
 0
 3.00295901327161E-04
 0
 2.42403674839711E-05
 0
 0
 0
 0
 2.78951699513229E-05
 0
 0
 9.46950481253762E-05

 0
 0
 0
 7.86431436285947E-05
 2.35788128866197E-03
 0
 3.00295901327161E-04
 0
 2.42403674839711E-05
 0
 0
 0
 0
 0
 0
 0
 9.46950481253762E-05

 0
 0
 0
 0
 0
 0
 0
 0
 0
 0
 0
 0
 0
 2.78951699513229E-05

 0
 0
 0
 0
 3.14697303466837E-04
 0
 0
 3.71026537673107E-05
 2.42403674839711E-05
 2.54902085736317E-05
 2.38531915570304E-04
 6.7668100197705E-04
 1.64120421575413E-03
 0
 4.17997400578665E-04
 0
 0
 7.04756881609937E-04

 0
 0
 0
 0
 3.14697303466837E-04
 0
 0
 0
 2.42403674839711E-05
 0
 3.18042554093738E-05
 6.359170861953E-04
 1.64120421575413E-03
 0
 4.17997400578665E-04
 0
 0
 6.77475970063746E-04

 0
 0
 0
 0
 2.8651545539518E-04
 0
 0
 0
 2.42403674839711E-05
 0
 3.18042554093738E-05
 6.359170861953E-04
 1.64120421575413E-03
 0
 4.17997400578665E-04
 0
 0
 6.77475970063746E-04

 0
 0
 0
 0
 2.8181848071657E-05

 0
 0
 0
 0
 0
 0
 0
 3.71026537673107E-05
 0
 2.54902085736317E-05
 2.0672766016093E-04
 4.076391578175E-05

 0
 0
 0
 0
 0
 0
 0
 3.71026537673107E-05
 0
 2.54902085736317E-05
 2.0672766016093E-04
 4.076391578175E-05

 0
 0
 0
 0
 0
 0
 0
 0
 0
 0
 0
 0
 0
 0
 0
 0
 0
 2.72809115461911E-05

 0
 0
 0
 0
 0
 0
 0
 0
 0
 0
 0
 0
 0
 0
 0
 0
 0
 2.72809115461911E-05

 0
 0
 4.67634309969604E-04
 0
 1.08030417608019E-04
 4.68853227628242E-04
 2.19728708288167E-05
 0
 1.15141745548863E-03
 0
 0
 0
 0
 3.25443649432101E-05
 0
 0
 2.2320975629553E-04

 0
 0
 4.67634309969604E-04
 0
 1.08030417608019E-04
 4.68853227628242E-04
 2.19728708288167E-05
 0
 1.15141745548863E-03
 0
 0
 0
 0
 3.25443649432101E-05
 0
 0
 2.2320975629553E-04

 0
 0
 4.67634309969604E-04
 0
 1.08030417608019E-04
 4.68853227628242E-04
 2.19728708288167E-05
 0
 1.15141745548863E-03
 0
 0
 0
 0
 3.25443649432101E-05
 0
 0
 2.2320975629553E-04

 0
 0
 1.61873414989478E-04
 2.97096320374691E-04
 3.24091252824056E-04
 3.62295675894551E-04
 1.14991357337474E-03
 0
 7.93872035100052E-04
 0
 8.90519151462466E-04
 0
 0
 0
 0
 3.84615384615385E-04
 3.65252328483594E-04

 0
 0
 1.61873414989478E-04
 2.97096320374691E-04
 3.24091252824056E-04
 3.62295675894551E-04
 1.14991357337474E-03
 0
 7.93872035100052E-04
 0
 8.90519151462466E-04
 0
 0
 0
 0
 3.84615384615385E-04
 3.65252328483594E-04

 0
 0
 1.61873414989478E-04
 2.97096320374691E-04
 3.24091252824056E-04
 3.62295675894551E-04
 1.14991357337474E-03
 0
 7.93872035100052E-04
 0
 8.90519151462466E-04
 0
 0
 0
 0
 3.84615384615385E-04
 3.65252328483594E-04

 0
 0
 0
 0
 0
 0
 0
 0
 0
 0
 6.51987235892162E-04

 0
 0
 0
 0
 0
 0
 0
 0
 0
 0
 6.51987235892162E-04

 0
 0
 0
 0
 0
 0
 0
 0
 0
 0
 6.51987235892162E-04

 0
 0
 0
 0
 0
 0
 0
 0
 0
 0
 0
 0
 2.14070115098365E-04
 0
 6.07402472715873E-04
 0
 0
 4.54681859103186E-05

 0
 0
 0
 0
 0
 0
 0
 0
 0
 0
 0
 0
 2.14070115098365E-04
 0
 6.07402472715873E-04
 0
 0
 4.54681859103186E-05

 0
 0
 0
 0
 0
 0
 0
 0
 0
 0
 0
 0
 2.14070115098365E-04
 0
 6.07402472715873E-04
 0
 0
 4.54681859103186E-05

 0
 0
 0
 0
 3.00606379431009E-04
 2.13115103467383E-04
 0
 0
 1.81802756129783E-04
 0
 9.01120569932257E-04
 1.2229174734525E-05
 2.85426820131154E-05
 0
 1.63280234601041E-04
 0
 3.11140872411951E-04
 1.81872743641274E-04

 0
 0
 0
 0
 3.00606379431009E-04
 2.13115103467383E-04
 0
 0
 1.81802756129783E-04
 0
 9.01120569932257E-04
 1.2229174734525E-05
 2.85426820131154E-05
 0
 1.63280234601041E-04
 0
 3.11140872411951E-04
 1.81872743641274E-04

 0
 0
 0
 0
 2.48939657966304E-04
 2.13115103467383E-04
 0
 0
 1.81802756129783E-04
 0
 5.98980143543206E-04
 1.2229174734525E-05
 2.85426820131154E-05
 0
 1.63280234601041E-04
 0
 3.11140872411951E-04
 1.81872743641274E-04

 0
 0
 0
 0
 5.16667214647046E-05
 0
 0
 0
 0
 0
 3.02140426389051E-04

 0
 0
 0
 0
 0
 0
 0
 0
 0
 0
 0
 1.7120844628335E-04
 2.14070115098365E-05
 0
 5.1596554133929E-04
 0
 0
 2.86449571235007E-04

 0
 0
 0
 0
 0
 0
 0
 0
 0
 0
 0
 1.7120844628335E-04
 2.14070115098365E-05
 0
 5.1596554133929E-04
 0
 0
 2.86449571235007E-04

 0
 0
 0
 0
 0
 0
 0
 0
 0
 0
 0
 1.7120844628335E-04
 2.14070115098365E-05
 0
 5.1596554133929E-04
 0
 0
 2.86449571235007E-04

 0
 0
 0
 0
 0
 0
 0
 0
 0
 0
 4.18756029556755E-04

 0
 0
 0
 0
 0
 0
 0
 0
 0
 0
 4.18756029556755E-04

 0
 0
 0
 0
 0
 0
 0
 0
 0
 0
 4.18756029556755E-04

 0
 0
 0
 3.23310701584222E-04
 0
 0
 3.2959306243225E-04
 0
 0
 0
 0
 0
 0
 0
 0
 0
 7.44032520985099E-05

 0
 0
 0
 3.23310701584222E-04
 0
 0
 3.2959306243225E-04
 0
 0
 0
 0
 0
 0
 0
 0
 0
 7.44032520985099E-05

 0
 0
 0
 3.23310701584222E-04
 0
 0
 3.2959306243225E-04
 0
 0
 0
 0
 0
 0
 0
 0
 0
 7.44032520985099E-05

 0
 0
 0
 0
 0
 0
 0
 0
 0
 0
 0
 .000326111326254
 0
 0
 0
 0
 0
 1.81872743641274E-05

 0
 0
 0
 0
 0
 0
 0
 0
 0
 0
 0
 .000326111326254
 0
 0
 0
 0
 0
 1.81872743641274E-05

 0
 0
 0
 0
 0
 0
 0
 0
 0
 0
 0
 .000326111326254
 0
 0
 0
 0
 0
 1.81872743641274E-05

 0
 0
 0
 0
 1.22121341643847E-04
 0
 0
 0
 2.8482431793666E-04
 1.91176564302237E-05
 4.2405673879165E-05

 0
 0
 0
 0
 1.22121341643847E-04
 0
 0
 0
 2.8482431793666E-04
 1.91176564302237E-05
 4.2405673879165E-05

 0
 0
 0
 0
 1.22121341643847E-04
 0
 0
 0
 2.60583950452689E-04
 1.91176564302237E-05
 4.2405673879165E-05

 0
 0
 0
 0
 0
 0
 0
 0
 2.42403674839711E-05

 0
 0
 1.25901544991816E-04
 0
 0
 2.13115103467383E-04
 8.05671930389945E-05
 0
 1.63622480516805E-04
 0
 7.95106385234344E-05
 3.26111326254E-05
 2.35477126608202E-04
 0
 1.11030559528708E-04
 2.24358974358974E-04
 2.02917960268663E-04
 2.54621841097784E-04

 0
 0
 1.25901544991816E-04
 0
 0
 2.13115103467383E-04
 8.05671930389945E-05
 0
 1.63622480516805E-04
 0
 7.95106385234344E-05
 3.26111326254E-05
 2.35477126608202E-04
 0
 1.11030559528708E-04
 2.24358974358974E-04
 2.02917960268663E-04
 2.54621841097784E-04

 0
 0
 1.25901544991816E-04
 0
 0
 2.13115103467383E-04
 8.05671930389945E-05
 0
 1.33322021161841E-04
 0
 2.12028369395825E-05
 3.26111326254E-05
 2.35477126608202E-04
 0
 1.11030559528708E-04
 2.24358974358974E-04
 2.02917960268663E-04
 2.54621841097784E-04

 0
 0
 0
 0
 0
 0
 0
 0
 3.03004593549638E-05
 0
 5.83078015838519E-05

 0
 0
 0
 0
 1.55000164394114E-04
 0
 4.39457416576334E-05
 0
 0
 0
 0
 0
 0
 0
 0
 0
 6.0875388080599E-05

 0
 0
 0
 0
 1.55000164394114E-04
 0
 4.39457416576334E-05
 0
 0
 0
 0
 0
 0
 0
 0
 0
 6.0875388080599E-05

 0
 0
 0
 0
 1.55000164394114E-04
 0
 4.39457416576334E-05
 0
 0
 0
 0
 0
 0
 0
 0
 0
 6.0875388080599E-05

 0
 0
 0
 0
 1.8787898714438E-05
 0
 0
 0
 8.48412861938987E-05
 0
 1.37818440107286E-04

 0
 0
 0
 0
 1.8787898714438E-05
 0
 0
 0
 8.48412861938987E-05
 0
 1.37818440107286E-04

 0
 0
 0
 0
 1.8787898714438E-05
 0
 0
 0
 8.48412861938987E-05
 0
 1.37818440107286E-04

 0
 0
 0
 0
 1.36212265679676E-04
 0
 7.32429027627223E-05

 0
 0
 0
 0
 1.36212265679676E-04
 0
 7.32429027627223E-05

 0
 0
 0
 0
 1.36212265679676E-04
 0
 7.32429027627223E-05

 0
 0
 0
 0
 0
 0
 0
 0
 1.03021561806877E-04
 0
 1.27217021637495E-04

 0
 0
 0
 0
 0
 0
 0
 0
 1.03021561806877E-04
 0
 1.27217021637495E-04

 0
 0
 0
 0
 0
 0
 0
 0
 1.03021561806877E-04
 0
 1.27217021637495E-04

 0
 0
 7.19437399953237E-05
 0
 6.10606708219236E-05
 1.06557551733691E-04
 0
 0
 0
 0
 0
 0
 0
 0
 3.91872563042499E-05
 0
 7.44032520985099E-05

 0
 0
 7.19437399953237E-05
 0
 6.10606708219236E-05
 1.06557551733691E-04
 0
 0
 0
 0
 0
 0
 0
 0
 3.91872563042499E-05
 0
 7.44032520985099E-05

 0
 0
 7.19437399953237E-05
 0
 6.10606708219236E-05
 1.06557551733691E-04
 0
 0
 0
 0
 0
 0
 0
 0
 3.91872563042499E-05
 0
 7.44032520985099E-05

 0
 0
 0
 0
 0
 0
 0
 0
 0
 0
 6.89092200536432E-05

 0
 0
 0
 0
 0
 0
 0
 0
 0
 0
 6.89092200536432E-05

 0
 0
 0
 0
 0
 0
 0
 0
 0
 0
 6.89092200536432E-05

 0
 0
 0
 0
 0
 0
 0
 0
 2.42403674839711E-05
 0
 0
 0
 0
 0
 0
 0
 0
 4.09213673192867E-05

 0
 0
 0
 0
 0
 0
 0
 0
 0
 0
 0
 0
 0
 0
 0
 0
 0
 4.09213673192867E-05

 0
 0
 0
 0
 0
 0
 0
 0
 0
 0
 0
 0
 0
 0
 0
 0
 0
 4.09213673192867E-05

 0
 0
 0
 0
 0
 0
 0
 0
 2.42403674839711E-05

 0
 0
 0
 0
 0
 0
 0
 0
 2.42403674839711E-05

 0
 0
 0
 0
 0
 0
 2.92971611050889E-05

 0
 0
 0
 0
 0
 0
 2.92971611050889E-05

 0
 0
 0
 0
 0
 0
 2.92971611050889E-05

 0
 0
 0
 0
 0
 0
 0
 0
 0
 0
 2.65035461744781E-05

 0
 0
 0
 0
 0
 0
 0
 0
 0
 0
 2.65035461744781E-05

 0
 0
 0
 0
 0
 0
 0
 0
 0
 0
 2.65035461744781E-05

 0
 .625
 .212539793881185
 .122447374629722
 .209682343602486
 .339577605864928
 .217289719626168
 9.79881085994675E-02
 .413328566061061
 .07376866361209
 6.37357278403851E-02
 .129539571571245
 .19636651657973
 .228140647446895
 .441072163332484
 .358108974358974
 .475774977509926
 .285517473423846

 0
 .625
 8.63324879943884E-04
 6.90312038517664E-04
 2.58333607323523E-04
 5.96722289708672E-04
 1.17921073447983E-03
 3.10734725301228E-04
 5.99949095228283E-04
 1.27451042868159E-04
 4.82894611298992E-03
 7.05215743024275E-04
 1.41999843015249E-03
 3.20794454440213E-03
 7.90798832219762E-02
 1.05769230769231E-03
 5.61406356743303E-04
 .00656560604545

 0
 .625
 8.63324879943884E-04
 6.90312038517664E-04
 2.58333607323523E-04
 5.96722289708672E-04
 1.17921073447983E-03
 3.10734725301228E-04
 5.99949095228283E-04
 1.27451042868159E-04
 4.82894611298992E-03
 7.05215743024275E-04
 1.41999843015249E-03
 3.20794454440213E-03
 7.90798832219762E-02
 1.05769230769231E-03
 5.61406356743303E-04
 .00656560604545

 0
 .625
 3.59718699976618E-04
 0
 0
 0
 0
 0
 0
 2.54902085736317E-05
 2.96839717154155E-04
 0
 0
 5.11411449107587E-05
 0
 0
 0
 3.63745487282548E-05

 0
 0
 0
 0
 0
 0
 0
 0
 0
 0
 0
 0
 0
 0
 6.79376400128012E-02

 0
 0
 2.33817154984802E-04
 3.49525082793754E-05
 4.22727721074856E-05
 3.19672655201074E-04
 0
 2.68994239813003E-04
 1.99983031742761E-04
 1.01960834294527E-04
 3.89602128764829E-03
 6.64451827242525E-04
 4.70954253216403E-04
 3.06846869464552E-03
 9.88171979805501E-03
 0
 1.01458980134332E-04
 4.59228677694217E-04

 0
 0
 0
 0
 6.10606708219236E-05
 0
 8.78914833152667E-05
 0
 0
 0
 0
 0
 1.14170728052461E-04
 0
 7.77213916700956E-04
 0
 0
 3.25097529258778E-03

 0
 0
 0
 0
 0
 0
 0
 4.17404854882245E-05
 0
 0
 0
 0
 6.42210345295096E-04
 4.64919499188715E-05
 3.00435631665916E-04
 0
 0
 1.68686969727282E-03

 0
 0
 2.69789024982464E-04
 6.55359530238289E-04
 1.22121341643847E-04
 2.77049634507598E-04
 1.09131925116456E-03
 0
 3.99966063485522E-04
 0
 0
 1.63055663127E-05
 8.56280460393461E-05
 0
 0
 1.05769230769231E-03
 4.32891648573149E-04
 3.09183664190166E-04

 0
 0
 0
 0
 3.28788227502666E-05
 0
 0
 0
 0
 0
 0
 0
 1.07035057549183E-04
 0
 1.82873862753166E-04
 0
 0
 7.72959160475415E-04

 0
 0
 0
 0
 0
 0
 0
 0
 0
 0
 2.33231206335408E-04

 0
 0
 0
 0
 0
 0
 0
 0
 0
 0
 1.80224113986451E-04

 0
 0
 0
 0
 0
 0
 0
 0
 0
 0
 1.37818440107286E-04

 0
 0
 0
 0
 0
 0
 0
 0
 0
 0
 0
 0
 0
 0
 0
 0
 0
 5.00150045013504E-05

 0
 0
 0
 0
 0
 0
 0
 0
 0
 0
 4.2405673879165E-05

 0
 0
 0
 0
 0
 0
 0
 0
 0
 0
 4.2405673879165E-05
 0
 0
 0
 0
 0
 2.70557280358218E-05

 0
 0
 0
 0
 0
 0
 0
 0
 0
 0
 0
 2.445834946905E-05
 0
 4.18427549269844E-05

 0
 0
 .132070720696415
 4.94752754694559E-02
 .149185309741995
 .199134752679922
 .11332874344476
 9.42082757469227E-02
 .349115832596022
 7.17740547912033E-02
 2.86291305776713E-02
 4.83623096834682E-02
 5.56368229140651E-02
 .195647423648595
 .193317266558248
 .188189102564103
 .249602618994474
 7.11622577682396E-02

 0
 0
 .132070720696415
 4.94752754694559E-02
 .149185309741995
 .199134752679922
 .11332874344476
 9.42082757469227E-02
 .349115832596022
 7.17740547912033E-02
 2.86291305776713E-02
 4.83623096834682E-02
 5.56368229140651E-02
 .195647423648595
 .193317266558248
 .188189102564103
 .249602618994474
 7.11622577682396E-02

 0
 0
 .046169895141999
 2.38113962653245E-02
 1.74116851336054E-02
 7.15001172133069E-02
 .04530805964902
 1.72620096652413E-02
 .261147538996691
 9.95392644800316E-03
 1.15661475505423E-02
 1.83070745775839E-02
 1.88310344581529E-02
 6.46796007271341E-02
 6.03483747085448E-03
 6.72115384615385E-02
 8.09439743511698E-02
 2.13473132848946E-02

 0
 0
 1.11512796992752E-03
 1.31071906047658E-04
 1.73788063108552E-03
 1.0016409862967E-03
 1.02540063867811E-04
 0
 8.58715018119675E-03
 0
 0
 5.706948209445E-05
 5.77989310765586E-04
 1.06466565314216E-03
 .128181515371201
 0
 1.44071751790751E-03
 3.21460074385952E-03

 0
 0
 5.75549919962589E-04
 1.86995919294658E-03
 9.26994922570372E-02
 7.03279841442363E-04
 1.10596783171711E-03
 0
 5.1934987334408E-03
 0
 0
 2.73118235737725E-04
 3.99597548183615E-04
 0
 1.45645969264129E-03
 0
 6.01989948797035E-04
 7.72959160475415E-04

 0
 0
 6.29507724959082E-03
 2.40298494420706E-03
 2.95909404752399E-04
 1.45557615668222E-02
 9.31649723141828E-03
 6.44658609207024E-04
 1.22413855794054E-03
 2.42156981449501E-04
 0
 6.1145873672625E-04
 3.0683383164099E-03
 2.12003291630054E-03
 6.46589729020123E-04
 3.94070512820513E-02
 5.12300210358285E-02
 5.06060909181845E-03

 0
 0
 1.52880447490063E-03
 0
 1.40909240358285E-04
 2.02459348294014E-03
 1.90431547183078E-04
 2.33004665658711E-02
 1.53320324336117E-03
 2.44897178871166E-02
 5.70886384598259E-03
 9.58359660028942E-03
 1.02825011952248E-02
 4.32049690596073E-02
 0
 0
 5.20822764689569E-04
 1.24082679349259E-02

 0
 0
 7.10444432453821E-03
 0
 2.20757809894647E-04
 4.68853227628242E-03
 2.78323030498345E-04
 .016362270311384
 7.8720593404196E-03
 6.74216016772557E-03
 1.9347588707369E-03
 9.66104804027475E-04
 8.27737778380345E-04
 2.49103867665314E-02
 3.76785469365362E-02
 0
 2.29973688304485E-03
 3.54651850100485E-04

 0
 0
 6.07924602960485E-03
 5.86328326386522E-03
 1.71909273237108E-03
 1.20410033459071E-02
 .018779480268362
 4.63783172091384E-05
 9.20527955203801E-03
 0
 0
 0
 0
 3.7658479434286E-04
 8.82366387784026E-03
 .03625
 3.61126329958131E-02

 0
 0
 2.26263062285293E-02
 2.79620066235003E-04
 4.18030746396246E-04
 2.83229972508152E-02
 1.20118360530865E-03
 0
 1.84832802065279E-02
 0
 0
 0
 0
 0
 0
 1.66666666666667E-03
 7.44708914185995E-03

 0
 0
 1.9784528498714E-03
 0
 0
 3.32459561409117E-03
 0
 1.76237605394726E-02
 4.48446798453465E-04
 1.29617710596917E-02
 4.88725391457377E-03
 2.0218902227748E-03
 5.20903946739355E-04
 2.32552733494195E-02
 1.95936281521249E-04
 0
 3.44960532456728E-04

 0
 0
 1.03239266893289E-02
 0
 1.64394113751333E-04
 1.84557679602753E-02
 0
 1.42520568783682E-02
 1.81802756129783E-03
 .012904418090401
 1.00183404539527E-03
 8.51965839838575E-04
 2.99698161137711E-04
 2.05122483042061E-02
 0
 0
 2.50265484331351E-03
 5.91086416834141E-05

 0
 0
 6.78069749455925E-03
 8.21383944565322E-04
 3.33015504713414E-03
 1.34475630287919E-02
 2.11671988984267E-03
 0
 2.35737573781619E-03
 0
 0
 0
 0
 0
 1.89405072137208E-04
 9.00641025641026E-03
 1.78364887076155E-02

 0
 0
 7.42819115451717E-03
 8.47598325774853E-04
 2.67727556680742E-04
 9.24919549048441E-03
 5.51519057803299E-03
 0
 4.49052807640564E-03
 0
 0
 0
 0
 0
 0
 8.79807692307692E-03
 7.71088249020921E-03

 0
 0
 1.20505764492167E-03
 0
 0
 1.06557551733691E-03
 0
 0
 8.45382816003491E-03
 0
 0
 5.3482257505656E-03
 9.2050149492297E-03
 1.91081914166562E-03
 2.71698310376132E-03
 0
 7.37268588976144E-04
 9.07090308910855E-03

 0
 0
 1.24102951491933E-03
 2.97096320374691E-04
 9.76970733150778E-04
 2.42951217952816E-03
 3.45706501040049E-03
 0
 4.24206430969493E-04
 0
 0
 0
 0
 0
 0
 5.28846153846154E-03
 8.54284612731073E-03

 0
 0
 0
 5.06811370050943E-04
 3.8515192364598E-04
 0
 4.48978993935488E-03
 0
 0
 0
 0
 0
 0
 0
 0
 8.49358974358974E-03
 8.45491501119431E-04

 0
 0
 8.27353009946222E-04
 3.13698761807394E-03
 5.11970239968436E-03
 2.81311936576945E-03
 8.35701520522661E-03
 1.36816035766958E-03
 1.60592434581308E-03
 1.91176564302237E-03
 1.14495319473746E-03
 0
 0
 3.50084382889103E-03
 0
 5.16025641025641E-03
 5.98607982792557E-03
 3.54651850100485E-04

 0
 0
 1.65470601989244E-03
 0
 2.06666885858818E-04
 1.87541291051297E-03
 7.76374769284856E-04
 4.63783172091384E-05
 3.5148532851758E-04
 0
 0
 7.47202576279477E-03
 5.46592360551159E-03
 6.55536493856089E-04
 3.79463265212819E-03
 2.72435897435897E-04
 8.34669209905102E-03
 3.40102030609183E-03

 0
 0
 3.86697602474865E-03
 6.64097657308133E-04
 7.90031140942119E-03
 5.20000852460414E-03
 1.91896405238332E-03
 1.06670129581018E-04
 1.0847564449077E-03
 0
 7.95106385234344E-05
 1.141389641889E-04
 1.12743593951806E-03
 1.85967799675486E-05
 0
 8.17307692307692E-04
 4.47095905791955E-03
 3.30099029708913E-03

 0
 0
 9.35268619939208E-04
 0
 2.16060835216037E-03
 8.09837393176054E-04
 4.10160255471245E-04
 2.34210501906149E-03
 3.27244961033609E-04
 1.87990288230533E-03
 5.61875178898937E-04
 1.78138311966247E-03
 2.49748467614759E-03
 6.66229642337429E-03
 2.15529909673374E-04
 0
 2.63793348349262E-04
 2.38253294170069E-03

 0
 0
 0
 6.29145149028757E-04
 6.20000657576455E-03
 6.39345310402148E-05
 1.46485805525445E-04
 0
 6.66610105809204E-05
 0
 0
 0
 0
 0
 0
 0
 1.89390096250752E-04

 0
 0
 1.79859349988309E-03
 1.98355484485455E-03
 1.16015274561655E-03
 2.68525030368902E-03
 4.7461400990244E-03
 1.06670129581018E-04
 1.13929727174664E-03
 0
 5.83078015838519E-05
 3.38340500988525E-04
 1.06321490498855E-03
 3.67286404359085E-04
 2.54717165977624E-04
 3.91025641025641E-03
 3.68634294488072E-03
 2.49620340647649E-03

 0
 0
 0
 4.41275417027114E-03
 3.8045494896737E-04
 0
 1.68458676354261E-04
 0
 1.03021561806877E-04
 0
 4.08154611086963E-04

 0
 0
 3.05760894980126E-04
 0
 0
 5.75410779361933E-04
 0
 2.22615922603864E-04
 3.86633861369338E-03
 1.65686355728606E-04
 0
 0
 0
 6.92730053791186E-04
 0
 0
 3.99071988528371E-04
 1.9551319941437E-04

 0
 0
 0
 6.90312038517664E-04
 2.25454784573256E-04
 0
 1.31104795945273E-03
 0
 0
 0
 0
 .000163055663127
 1.1131645985115E-03
 1.58072629724163E-04
 4.04934981810582E-04
 6.73076923076923E-04
 8.79311161164208E-04
 3.79204670492057E-03

 0
 0
 5.39578049964927E-04
 0
 1.04272837865131E-03
 0
 0
 0
 2.79370235252766E-03
 0
 0
 0
 0
 1.34826654764727E-04
 3.13498050433999E-04
 0
 1.11604878147765E-03
 8.36614620749861E-04

 0
 0
 0
 0
 0
 0
 0
 0
 2.20587344104137E-03

 0
 0
 2.51803089983633E-04
 3.32048828654066E-04
 2.19818414958925E-03
 2.55738124160859E-04
 9.81454897020479E-04
 6.49296440927937E-05
 1.21201837419855E-04
 1.08333386437935E-04
 8.32211349878614E-04
 1.141389641889E-04
 6.42210345295096E-05
 1.76669409691712E-04
 0
 2.24358974358974E-04
 8.38727569110475E-04
 7.27490974565097E-05

 0
 0
 2.15831219985971E-04
 0
 1.40909240358285E-04
 0
 0
 0
 1.92104912310471E-03
 0
 0
 0
 0
 0
 1.46952211140937E-03
 0
 4.73475240626881E-04

 0
 0
 0
 1.92238795536565E-04
 1.56878954265558E-03
 0
 2.34377288840711E-04
 0
 6.42369738325233E-04
 0
 1.27217021637495E-04
 3.30187717832175E-04
 2.92562490634432E-04
 6.0439534894533E-05
 0
 0
 1.4880650419702E-04
 6.91116425836842E-04

 0
 0
 8.45338944945053E-04
 1.31071906047658E-04
 2.34848733930475E-04
 1.42787119323146E-03
 1.09864354144083E-04
 4.26680518324073E-04
 7.09030748906153E-04
 4.14215889321514E-04
 0
 2.8534741047225E-05
 0
 8.60101073499124E-04
 3.13498050433999E-04
 1.92307692307692E-04
 1.35955033380004E-03

 0
 0
 0
 3.67001336933442E-04
 0
 0
 1.36231799138663E-03
 0
 0
 0
 0
 0
 0
 0
 0
 8.17307692307692E-04
 3.04376940402995E-04

 0
 0
 0
 0
 0
 0
 0
 0
 1.03021561806877E-04
 0
 0
 0
 0
 0
 0
 0
 7.10212860940322E-04

 0
 0
 0
 0
 0
 0
 0
 0
 0
 0
 0
 0
 0
 0
 0
 0
 0
 6.59288695699619E-04

 0
 0
 3.77704634975449E-04
 0
 0
 6.1803380005541E-04
 0
 0
 4.12086247227508E-04
 0
 0
 0
 0
 0
 0
 0
 3.17904804420906E-04

 0
 0
 0
 0
 0
 0
 0
 0
 0
 0
 0
 0
 0
 0
 5.02903122571207E-04

 0
 0
 0
 1.04857524838126E-04
 4.36818645110684E-04
 0
 3.36917352708523E-04
 0
 0
 0
 0
 0
 0
 0
 0
 0
 1.82626164241797E-04
 4.50135040512154E-04

 0
 0
 0
 0
 7.04546201791426E-05
 0
 3.58890223537339E-04
 0
 0
 0
 0
 0
 0
 0
 0
 0
 7.44032520985099E-05

 0
 0
 0
 0
 0
 0
 0
 0
 0
 0
 1.16615603167704E-04
 0
 0
 0
 0
 0
 2.84085144376129E-04

 0
 0
 0
 0
 2.58333607323523E-04

 0
 0
 0
 0
 0
 0
 2.49025869393256E-04
 0
 0
 0
 0
 0
 0
 0
 0
 0
 2.36737620313441E-04

 0
 0
 0
 0
 0
 0
 0
 0
 2.24223399226732E-04
 0
 2.01426950926034E-04

 0
 0
 0
 0
 0
 0
 0
 0
 0
 0
 0
 0
 0
 1.02282289821517E-04
 0
 0
 2.16445824286574E-04

 0
 0
 0
 0
 0
 0
 0
 0
 1.99983031742761E-04
 0
 0
 0
 0
 0
 0
 0
 0
 4.54681859103185E-06

 0
 0
 0
 0
 0
 0
 0
 3.24648220463969E-05
 0
 0
 0
 0
 0
 7.90363148620816E-05
 0
 0
 0
 1.54591832095083E-04

 0
 0
 0
 0
 0
 0
 0
 0
 0
 0
 0
 0
 0
 1.44125044748502E-04

 0
 0
 0
 0
 0
 0
 0
 0
 0
 0
 0
 0
 0
 0
 1.24092978296791E-04

 0
 0
 0
 0
 1.12727392286628E-04

 0
 0
 0
 0
 0
 0
 0
 0
 0
 0
 0
 0
 0
 0
 0
 0
 0
 8.18427346385734E-05

 0
 0
 8.93900969441896E-03
 1.00575842573903E-02
 5.68803633579611E-03
 2.25049549261556E-02
 1.65382474438227E-02
 0
 2.64826014762384E-03
 0
 7.42099292885388E-05
 1.7120844628335E-04
 1.19165697404757E-03
 7.43871198701945E-05
 8.19340217228024E-02
 1.53365384615385E-02
 4.19566702515507E-02
 4.54227177244082E-03

 0
 0
 7.64402237450314E-03
 9.40222472715198E-03
 4.48561081807208E-03
 2.02246233190546E-02
 1.51759294524361E-02
 0
 .001678645448265
 0
 0
 1.5490287997065E-04
 1.1131645985115E-03
 0
 8.18295223726577E-02
 1.35576923076923E-02
 3.80335896863565E-02
 4.46952267498431E-03

 0
 0
 7.64402237450314E-03
 9.40222472715198E-03
 4.48561081807208E-03
 2.02246233190546E-02
 1.51759294524361E-02
 0
 .001678645448265
 0
 0
 1.5490287997065E-04
 1.1131645985115E-03
 0
 8.18295223726577E-02
 1.35576923076923E-02
 3.80335896863565E-02
 4.46952267498431E-03

 0
 0
 9.17282684940377E-04
 2.62143812095315E-04
 1.55000164394114E-04
 1.15082155872387E-03
 4.54105997128878E-04
 0
 6.7873028955119E-04
 0
 0
 0
 0
 0
 0
 7.21153846153846E-04
 2.06976319474037E-03

 0
 0
 9.17282684940377E-04
 2.62143812095315E-04
 1.55000164394114E-04
 1.15082155872387E-03
 4.54105997128878E-04
 0
 6.7873028955119E-04
 0
 0
 0
 0
 0
 0
 7.21153846153846E-04
 2.06976319474037E-03

 0
 0
 3.77704634975449E-04
 3.93215718142973E-04
 1.04742535332992E-03
 1.12951004837713E-03
 9.08211994257756E-04
 0
 2.90884409807653E-04
 0
 0
 1.63055663127E-05
 7.84923755360672E-05
 7.43871198701945E-05
 1.04499350144666E-04
 1.05769230769231E-03
 1.85331737045379E-03
 7.27490974565097E-05

 0
 0
 3.77704634975449E-04
 3.93215718142973E-04
 1.04742535332992E-03
 9.8032947594996E-04
 9.08211994257756E-04
 0
 2.90884409807653E-04
 0
 0
 1.63055663127E-05
 7.84923755360672E-05
 4.18427549269844E-05
 1.04499350144666E-04
 1.05769230769231E-03
 1.8059698463911E-03
 7.27490974565097E-05

 0
 0
 0
 0
 0
 1.49180572427168E-04
 0
 0
 0
 0
 0
 0
 0
 3.25443649432101E-05
 0
 0
 4.73475240626881E-05

 0
 0
 0
 0
 0
 0
 0
 0
 0
 0
 7.42099292885388E-05

 0
 0
 0
 0
 0
 0
 0
 0
 0
 0
 7.42099292885388E-05

 0
 0
 1.55218619039911E-02
 2.58124273643187E-02
 2.52133600747759E-02
 3.56967798307866E-02
 3.36990595611285E-02
 1.86440835180736E-03
 9.43556304313574E-03
 1.18529469867387E-03
 2.19979433248168E-03
 7.54784664614882E-02
 .107134956936229
 1.61838477667592E-02
 4.24855170431908E-02
 7.52564102564103E-02
 5.78113268805422E-02
 .148967417497977

 0
 0
 1.52880447490063E-02
 2.52881397401281E-02
 2.49409355434165E-02
 3.53131726445453E-02
 .032878739050186
 1.66034375608715E-03
 9.34466166507085E-03
 8.28431778643028E-04
 7.1559574671091E-04
 7.47080284532131E-02
 .106585510307476
 1.55980491977814E-02
 4.24397985775025E-02
 7.48717948717949E-02
 5.75340056681751E-02
 .148821919303064

 0
 0
 4.33461033471825E-03
 2.04472173434346E-03
 6.85758303076988E-04
 7.58689768343883E-03
 3.15676910907333E-03
 8.39447541485405E-04
 2.53311840207498E-03
 4.84313962899001E-04
 1.16615603167704E-04
 4.96341438558588E-02
 5.55583305385291E-02
 7.59678461674361E-03
 .016158212016119
 4.26282051282051E-03
 5.35703415109271E-03
 7.65456909800213E-02

 0
 0
 1.79859349988309E-04
 1.92238795536565E-04
 3.19394278145447E-04
 1.08688702768365E-03
 7.52204611373158E-03
 0
 8.48412861938987E-05
 0
 0
 0
 0
 0
 0
 4.48717948717949E-02
 2.63793348349262E-03

 0
 0
 2.75184805482113E-03
 6.64097657308133E-04
 3.53212495831435E-03
 5.92459987639324E-03
 4.18216974775144E-03
 2.04064595720209E-04
 1.49078260026422E-03
 1.97549116445645E-04
 1.21916312402599E-04
 1.11937712736685E-02
 2.23631913572759E-02
 2.70583148527832E-03
 8.09869963621164E-03
 7.70833333333333E-03
 9.86181286905704E-03
 .03639728282121

 0
 0
 6.18716163959783E-03
 4.19430099352505E-04
 8.92425188935806E-05
 1.64737974980287E-02
 4.46781706852606E-04
 3.61750874231279E-04
 3.90269916491934E-03
 7.0098073577487E-05
 1.11314893932808E-04
 1.18215355767075E-04
 0
 2.44547656573264E-03
 3.26560469202082E-05
 1.28205128205128E-03
 3.19325230142787E-02

 0
 0
 1.24102951491933E-03
 7.15652607020211E-03
 1.47625914148697E-02
 3.19672655201074E-03
 1.30445609820408E-02
 9.27566344182768E-05
 4.66627074066443E-04
 0
 9.01120569932257E-05
 1.24003831808083E-02
 2.35048986378005E-02
 1.22273828286632E-03
 1.08744636244293E-02
 8.41346153846154E-03
 3.46989712059414E-03
 3.12684714505261E-02

 0
 0
 0
 1.38586695327723E-02
 5.17136912114907E-03
 0
 2.92971611050889E-04
 0
 0
 0
 0
 0
 0
 1.39475849756615E-05
 0
 3.20512820512821E-04

 0
 0
 0
 2.62143812095315E-04
 2.25454784573256E-04
 0
 2.98831043271907E-03
 1.39134951627415E-05
 0
 0
 0
 2.8534741047225E-05
 0
 6.50887298864202E-05
 1.95936281521249E-05
 7.11538461538462E-03
 3.24668736429861E-04
 4.09213673192867E-05

 0
 0
 1.25901544991816E-04
 0
 9.39394935721902E-05
 1.2786906208043E-04
 1.09864354144083E-04
 1.15945793022846E-04
 0
 0
 0
 1.42673705236125E-04
 3.63919195667221E-04
 1.3389681576635E-03
 7.06676855353306E-03
 5.12820512820513E-04
 8.18435773083609E-04
 2.54621841097784E-04

 0
 0
 0
 6.90312038517664E-04
 6.10606708219236E-05
 0
 1.1352649928222E-03
 3.24648220463969E-05
 0
 7.6470625720895E-05
 2.75636880214573E-04
 1.1903063408271E-03
 4.79517057820338E-03
 9.76330948296303E-05
 1.89405072137208E-04
 3.84615384615385E-04
 1.14986844152243E-04
 4.31493084288923E-03

 0
 0
 4.67634309969604E-04
 0
 0
 9.16394944909746E-04
 0
 0
 8.66593137551965E-04
 0
 0
 0
 0
 1.11580679805292E-04
 0
 0
 3.01671367599413E-03

 0
 0
 0
 0
 0
 0
 0
 2.04064595720209E-04
 0
 3.56862920030843E-04
 1.38878581954265E-03
 0
 4.2814023019673E-05
 5.85798568977782E-04

 0
 0
 0
 0
 0
 0
 0
 1.66961941952898E-04
 0
 3.56862920030843E-04
 1.15025390397235E-03
 0
 0
 2.97548479480778E-04

 0
 0
 0
 0
 0
 0
 0
 3.71026537673107E-05
 0
 0
 1.74923404751556E-04
 0
 0
 2.88250089497004E-04

 0
 0
 0
 0
 0
 0
 0
 0
 0
 0
 6.36085108187475E-05
 0
 4.2814023019673E-05

 0
 0
 2.33817154984802E-04
 5.24287624190631E-04
 2.53636632644913E-04
 3.83607186241289E-04
 8.2032051094249E-04
 0
 9.09013780648915E-05
 0
 9.54127662281213E-05
 7.70438008275075E-04
 5.06632605732798E-04
 0
 0
 3.84615384615385E-04
 2.77321212367173E-04
 1.45498194913019E-04

 0
 0
 2.33817154984802E-04
 5.24287624190631E-04
 2.53636632644913E-04
 3.83607186241289E-04
 8.2032051094249E-04
 0
 9.09013780648915E-05
 0
 0
 0
 0
 0
 0
 3.84615384615385E-04
 2.77321212367173E-04

 0
 0
 0
 0
 0
 0
 0
 0
 0
 0
 0
 7.70438008275075E-04
 5.06632605732798E-04
 0
 0
 0
 0
 1.45498194913019E-04

 0
 0
 0
 0
 0
 0
 0
 0
 0
 0
 9.54127662281213E-05

 0
 0
 0
 0
 1.8787898714438E-05
 0
 0
 0
 0
 0
 0
 0
 0
 0
 4.57184656882915E-05

 0
 0
 0
 0
 1.8787898714438E-05
 0
 0
 0
 0
 0
 0
 0
 0
 0
 4.57184656882915E-05

 0
 0
 1.12591953092682E-02
 4.6836361094363E-03
 1.28697106193901E-03
 2.29311851330904E-02
 6.45269973339583E-03
 3.71026537673107E-05
 3.97542026737125E-03
 1.91176564302237E-05
 5.77777306603624E-04
 1.42673705236125E-04
 3.19678038546892E-03
 1.95266189659261E-04
 6.92308194708414E-04
 6.81089743589744E-03
 4.21257685517745E-02
 9.02998172178926E-03

 0
 0
 1.12591953092682E-02
 4.6836361094363E-03
 1.28697106193901E-03
 2.29311851330904E-02
 6.45269973339583E-03
 3.71026537673107E-05
 3.97542026737125E-03
 1.91176564302237E-05
 5.77777306603624E-04
 1.42673705236125E-04
 3.19678038546892E-03
 1.95266189659261E-04
 6.92308194708414E-04
 6.81089743589744E-03
 4.21257685517745E-02
 9.02998172178926E-03

 0
 0
 1.12591953092682E-02
 4.6836361094363E-03
 1.28697106193901E-03
 2.29311851330904E-02
 6.45269973339583E-03
 3.71026537673107E-05
 3.97542026737125E-03
 1.91176564302237E-05
 5.77777306603624E-04
 1.42673705236125E-04
 3.19678038546892E-03
 1.95266189659261E-04
 6.92308194708414E-04
 6.81089743589744E-03
 4.21257685517745E-02
 9.02998172178926E-03

 0
 0
 5.75549919962589E-04
 0
 2.20757809894647E-04
 6.39345310402148E-04
 1.09864354144083E-04
 1.1409066033448E-03
 2.72704134194674E-04
 3.69608024317659E-04
 2.49133334040095E-04
 3.46493284144875E-04
 4.99496935229519E-05
 8.36855098539687E-04
 2.36103219233105E-02
 1.44230769230769E-04
 3.04376940402995E-04

 0
 0
 0
 0
 8.92425188935806E-05
 0
 0
 1.01568514688013E-03
 0
 3.25000159313804E-04
 2.49133334040095E-04
 4.4840307359925E-05
 0
 2.23161359610583E-04
 2.36103219233105E-02

 0
 0
 0
 0
 8.92425188935806E-05
 0
 0
 1.01568514688013E-03
 0
 3.25000159313804E-04
 2.49133334040095E-04
 4.4840307359925E-05
 0
 2.23161359610583E-04
 2.36103219233105E-02

 0
 0
 5.75549919962589E-04
 0
 1.31515291001066E-04
 6.39345310402148E-04
 1.09864354144083E-04
 1.25221456464674E-04
 2.72704134194674E-04
 4.46078650038554E-05
 0
 3.0165297678495E-04
 4.99496935229519E-05
 6.13693738929104E-04
 0
 1.44230769230769E-04
 3.04376940402995E-04

 0
 0
 5.75549919962589E-04
 0
 1.31515291001066E-04
 6.39345310402148E-04
 1.09864354144083E-04
 1.25221456464674E-04
 2.72704134194674E-04
 4.46078650038554E-05
 0
 3.0165297678495E-04
 4.99496935229519E-05
 6.13693738929104E-04
 0
 1.44230769230769E-04
 3.04376940402995E-04

 0
 0
 1.24102951491933E-03
 8.34491135170088E-03
 8.35591795324632E-03
 3.19672655201074E-03
 9.31649723141828E-03
 1.39134951627415E-05
 2.0119505011696E-03
 0
 5.83078015838519E-05
 7.86743574587775E-04
 1.49920437273888E-02
 6.18342933920991E-04
 5.57765281397156E-03
 2.39903846153846E-02
 5.34350628707481E-03
 4.99240681295298E-03

 0
 0
 1.20505764492167E-03
 7.24390734090055E-03
 6.14833985429985E-03
 2.85574238646293E-03
 7.88093633726892E-03
 0
 1.98771013368563E-03
 0
 3.71049646442694E-05
 5.2993090516275E-04
 2.68301210923284E-03
 4.18427549269844E-05
 3.77503902397607E-03
 2.23076923076923E-02
 4.18010998153447E-03
 3.71929760746406E-03

 0
 0
 1.20505764492167E-03
 7.24390734090055E-03
 6.14833985429985E-03
 2.85574238646293E-03
 7.88093633726892E-03
 0
 1.98771013368563E-03
 0
 3.71049646442694E-05
 5.2993090516275E-04
 2.68301210923284E-03
 4.18427549269844E-05
 3.77503902397607E-03
 2.23076923076923E-02
 4.18010998153447E-03
 3.71929760746406E-03

 0
 0
 3.59718699976618E-05
 1.10100401080033E-03
 2.20757809894647E-03
 3.40984165547812E-04
 1.43556089414936E-03
 1.39134951627415E-05
 2.42403674839711E-05
 0
 2.12028369395825E-05
 2.56812669425025E-04
 .012309031618156
 5.76500178994007E-04
 1.80261378999549E-03
 1.68269230769231E-03
 1.16339630554034E-03
 1.27310920548892E-03

 0
 0
 3.59718699976618E-05
 1.10100401080033E-03
 2.20757809894647E-03
 3.40984165547812E-04
 1.43556089414936E-03
 1.39134951627415E-05
 2.42403674839711E-05
 0
 2.12028369395825E-05
 2.56812669425025E-04
 .012309031618156
 5.76500178994007E-04
 1.80261378999549E-03
 1.68269230769231E-03
 1.16339630554034E-03
 1.27310920548892E-03

 0
 0
 1.71945538588823E-02
 6.70214346257024E-03
 1.18880429115607E-02
 2.22492168019948E-02
 1.51100108399496E-02
 7.42053075346215E-05
 8.90833505035936E-03
 3.82353128604475E-05
 1.27747092560985E-03
 1.2881397387033E-03
 6.39356077093785E-03
 3.4404042939965E-04
 8.29463591773288E-03
 2.96634615384615E-02
 3.28388899034787E-02
 2.83312266407195E-02

 0
 0
 5.44973830464576E-03
 5.36521002088413E-03
 9.20607037007464E-04
 1.04639515802485E-02
 1.04590865145167E-02
 1.85513268836554E-05
 1.1695977311016E-03
 3.82353128604475E-05
 5.83078015838519E-04
 .000326111326254
 1.52703348770168E-03
 1.11580679805292E-04
 1.24092978296791E-03
 2.57852564102564E-02
 1.83234918122603E-02
 8.07514981767258E-03

 0
 0
 4.01086350473929E-03
 3.87099029194083E-03
 3.61667050252932E-04
 8.16230846280076E-03
 4.44584419769724E-03
 0
 6.54489922067219E-04
 0
 5.83078015838519E-05
 2.0381957890875E-05
 6.42210345295096E-05
 3.71935599350972E-05
 8.49057219925414E-05
 1.83974358974359E-02
 1.41569096947437E-02
 3.41011394327389E-04

 0
 0
 1.43887479990647E-03
 1.48548160187345E-04
 3.8515192364598E-04
 2.30164311744773E-03
 3.66214513813611E-04
 1.85513268836554E-05
 5.15107809034385E-04
 0
 0
 3.05729368363125E-04
 1.3843200776361E-03
 7.43871198701945E-05
 1.15602406097537E-03
 5.28846153846154E-04
 1.71127479826573E-03
 7.73413842334519E-03

 0
 0
 0
 1.34567156875595E-03
 1.73788063108552E-04
 0
 5.64702780300589E-03
 0
 0
 0
 0
 0
 0
 0
 0
 6.85897435897436E-03
 2.36737620313441E-03

 0
 0
 0
 0
 0
 0
 0
 0
 0
 3.82353128604475E-05
 5.24770214254667E-04
 0
 7.84923755360672E-05
 0
 0
 0
 8.79311161164208E-05

 0
 0
 1.09894062842857E-02
 1.04857524838126E-03
 5.21364189325656E-03
 1.06770666837159E-02
 3.21536343128352E-03
 0
 6.21765425963857E-03
 0
 2.65035461744781E-05
 6.48146260929825E-04
 3.13969502144269E-03
 1.99915384651148E-04
 4.92453187556739E-03
 3.49358974358974E-03
 1.21209661600482E-02
 1.45952876772123E-02

 0
 0
 3.05760894980126E-04
 2.70881939165159E-04
 4.04409519828279E-03
 0
 7.17780447074678E-04
 0
 1.21201837419855E-04
 0
 2.65035461744781E-05
 6.48146260929825E-04
 2.5973840631935E-03
 1.11580679805292E-04
 4.15384916825048E-03
 3.36538461538462E-04
 7.30504656967188E-04
 9.42555493920904E-03

 0
 0
 6.97854277954639E-03
 2.97096320374691E-04
 7.6090989793474E-04
 6.69181424887582E-03
 1.06934638033575E-03
 0
 3.4300119989819E-03
 0
 0
 0
 0
 0
 0
 1.61858974358974E-03
 5.83050939171959E-03

 0
 0
 0
 2.97096320374691E-04
 1.12727392286628E-04
 0
 1.24512934696628E-04
 0
 0
 0
 0
 0
 3.85326207177057E-04
 0
 5.42090378875456E-04
 0
 0
 4.36494584739058E-03

 0
 0
 1.04318422993219E-03
 1.83500668466721E-04
 2.16060835216037E-04
 1.21475608976408E-03
 1.30372366917646E-03
 0
 8.60533045680972E-04
 0
 0
 0
 0
 0
 0
 1.53846153846154E-03
 3.82162158505983E-03

 0
 0
 2.5000449648375E-03
 0
 0
 2.77049634507598E-03
 0
 0
 1.80590737755584E-03
 0
 0
 0
 0
 8.83347048458559E-05
 0
 0
 1.25132742165676E-03

 0
 0
 0
 0
 3.28788227502666E-05
 0
 0
 0
 0
 0
 0
 0
 1.56984751072134E-04
 0
 2.28592328441457E-04
 0
 2.57029416340307E-04
 8.04786890612638E-04

 0
 0
 1.61873414989478E-04
 0
 4.69697467860951E-05
 0
 0
 0
 0
 0
 0
 0
 0
 0
 0
 0
 1.96154028259708E-04

 0
 0
 0
 0
 0
 0
 0
 0
 0
 0
 0
 0
 0
 0
 0
 0
 3.38196600447772E-05

 0
 0
 1.07915609992985E-04
 0
 7.51515948577521E-04
 0
 3.2959306243225E-04
 5.56539806509661E-05
 6.66610105809204E-05
 0
 3.44546100268215E-04
 3.13882151519475E-04
 1.30582770210003E-03
 3.25443649432101E-05
 1.22133615481579E-03
 0
 4.32891648573149E-04
 3.18731983231333E-03

 0
 0
 0
 0
 1.69091088429942E-04
 0
 0
 0
 0
 0
 1.21916312402599E-04
 2.56812669425025E-04
 1.30582770210003E-03
 3.25443649432101E-05
 1.18868010789558E-03
 0
 0
 3.18731983231333E-03

 0
 0
 1.07915609992985E-04
 0
 5.54243012075922E-04
 0
 2.56350159669528E-04
 0
 0
 0
 0
 0
 0
 0
 0
 0
 4.32891648573149E-04

 0
 0
 0
 0
 2.8181848071657E-05
 0
 7.32429027627223E-05
 5.56539806509661E-05
 6.66610105809204E-05
 0
 2.22629787865616E-04
 5.706948209445E-05
 0
 0
 3.26560469202082E-05

 0
 0
 8.99296749941546E-05
 6.99050165587508E-05
 .003259700426955
 1.70492082773906E-04
 4.68754577681422E-04
 0
 5.93889003357291E-04
 0
 0
 0
 1.92663103588529E-04
 0
 3.78810144274415E-04
 0
 7.5756038500301E-04
 1.05486191311939E-03

 0
 0
 0
 6.99050165587508E-05
 2.5692451491994E-03
 0
 2.636744499458E-04
 0
 7.27211024519132E-05
 0
 0
 0
 1.92663103588529E-04
 0
 3.78810144274415E-04
 0
 2.02917960268663E-04
 9.95753271435976E-04

 0
 0
 8.99296749941546E-05
 0
 6.90455277755598E-04
 1.70492082773906E-04
 2.05080127735622E-04
 0
 5.21167900905378E-04
 0
 0
 0
 0
 0
 0
 0
 5.54642424734347E-04
 5.91086416834141E-05

 0
 0
 0
 0
 1.19772854304543E-03
 0
 1.24512934696628E-04
 0
 9.69614699358842E-05
 0
 7.95106385234344E-05
 0
 9.98993870459038E-05
 0
 1.56749025216999E-04
 0
 4.05835920537327E-05
 7.13850518792001E-04

 0
 0
 0
 0
 1.17424366965238E-03
 0
 0
 0
 9.69614699358842E-05
 0
 7.95106385234344E-05
 0
 0
 0
 0
 0
 4.05835920537327E-05

 0
 0
 0
 0
 2.34848733930475E-05
 0
 1.24512934696628E-04
 0
 0
 0
 0
 0
 9.98993870459038E-05
 0
 1.56749025216999E-04
 0
 0
 7.13850518792001E-04

 0
 0
 5.57563984963758E-04
 2.18453176746096E-04
 2.25454784573256E-04
 9.37706455256484E-04
 5.12700319339056E-04
 0
 6.7873028955119E-04
 0
 3.71049646442694E-05
 0
 0
 0
 9.79681407606246E-05
 3.84615384615385E-04
 1.16339630554034E-03

 0
 0
 5.57563984963758E-04
 2.18453176746096E-04
 2.25454784573256E-04
 9.37706455256484E-04
 5.12700319339056E-04
 0
 6.7873028955119E-04
 0
 0
 0
 0
 0
 9.79681407606246E-05
 3.84615384615385E-04
 1.16339630554034E-03

 0
 0
 0
 0
 0
 0
 0
 0
 0
 0
 3.71049646442694E-05

 0
 0
 0
 0
 3.19394278145447E-04
 0
 0
 0
 0
 0
 0
 0
 0
 0
 2.74310794129749E-04
 0
 0
 7.04756881609938E-04

 0
 0
 0
 0
 3.19394278145447E-04
 0
 0
 0
 0
 0
 0
 0
 0
 0
 2.74310794129749E-04
 0
 0
 7.04756881609938E-04

 0
 0
 0
 0
 0
 0
 0
 0
 0
 0
 1.53720567811973E-04

 0
 0
 0
 0
 0
 0
 0
 0
 0
 0
 1.53720567811973E-04

 0
 0
 0
 0
 0
 0
 0
 0
 8.48412861938987E-05

 0
 0
 0
 0
 0
 0
 0
 0
 8.48412861938987E-05

 0
 0
 0
 0
 0
 0
 0
 0
 0
 0
 5.30070923489563E-05
 0
 1.28442069059019E-04

 0
 0
 0
 0
 0
 0
 0
 0
 0
 0
 0
 0
 7.84923755360672E-05

 0
 0
 0
 0
 0
 0
 0
 0
 0
 0
 5.30070923489563E-05

 0
 0
 0
 0
 0
 0
 0
 0
 0
 0
 0
 0
 4.99496935229519E-05

 0
 0
 2.57198870483282E-03
 3.16320199928347E-03
 1.85060802337215E-03
 7.13935596615732E-03
 6.60650982919755E-03
 0
 3.05428630298035E-03
 1.27451042868158E-05
 4.2405673879165E-05
 1.17807716609257E-03
 1.80532463732955E-03
 1.04281443668029E-02
 5.42090378875456E-04
 4.16666666666666E-03
 1.96221667579797E-02
 4.33766493584439E-03

 0
 0
 1.9784528498714E-03
 3.00591571202628E-03
 7.37425024541693E-04
 5.92459987639324E-03
 5.69097354466352E-03
 0
 3.99966063485522E-04
 0
 0
 1.05578541874732E-03
 1.49849080568856E-03
 .010218930592168
 1.43686606448916E-04
 3.50961538461538E-03
 1.72142069627916E-02
 3.6829230587358E-03

 0
 0
 1.9784528498714E-03
 3.00591571202628E-03
 7.37425024541693E-04
 5.92459987639324E-03
 5.69097354466352E-03
 0
 3.99966063485522E-04
 0
 0
 1.05578541874732E-03
 1.49849080568856E-03
 .010218930592168
 1.43686606448916E-04
 3.50961538461538E-03
 1.72142069627916E-02
 3.6829230587358E-03

 0
 0
 1.25901544991816E-04
 0
 1.69091088429942E-04
 0
 0
 0
 1.20595828232756E-03
 0
 0
 0
 0
 0
 0
 0
 5.68170288752257E-04

 0
 0
 1.25901544991816E-04
 0
 1.69091088429942E-04
 0
 0
 0
 1.20595828232756E-03
 0
 0
 0
 0
 0
 0
 0
 5.68170288752257E-04

 0
 0
 8.99296749941546E-05
 1.57286287257189E-04
 4.83788391896779E-04
 5.54099269015195E-04
 4.83403158233967E-04
 0
 6.60550013938212E-04
 0
 4.2405673879165E-05
 0
 3.56783525163942E-05
 1.48774239740389E-04
 1.04499350144666E-04
 0
 8.86075093173163E-04
 5.41071412332791E-04

 0
 0
 8.99296749941546E-05
 7.86431436285947E-05
 4.27424695753465E-04
 5.54099269015195E-04
 2.34377288840711E-04
 0
 6.24189462712255E-04
 0
 4.2405673879165E-05
 0
 3.56783525163942E-05
 1.48774239740389E-04
 1.04499350144666E-04
 0
 8.86075093173163E-04
 5.04696863604536E-04

 0
 0
 0
 7.86431436285947E-05
 5.63636961433141E-05
 0
 2.49025869393256E-04
 0
 3.63605512259566E-05

 0
 0
 0
 0
 0
 0
 0
 0
 0
 0
 0
 0
 0
 0
 0
 0
 0
 3.63745487282548E-05

 0
 0
 3.23746829978956E-04
 0
 1.36212265679676E-04
 2.77049634507598E-04
 3.14944481879706E-04
 0
 7.27211024519132E-04
 0
 0
 1.2229174734525E-04
 2.71155479124596E-04
 3.25443649432101E-05
 2.93904422281874E-04
 5.12820512820513E-04
 3.38196600447772E-04
 1.13670464775796E-04

 0
 0
 3.23746829978956E-04
 0
 1.36212265679676E-04
 2.77049634507598E-04
 3.14944481879706E-04
 0
 7.27211024519132E-04
 0
 0
 1.2229174734525E-04
 2.71155479124596E-04
 3.25443649432101E-05
 2.93904422281874E-04
 5.12820512820513E-04
 3.38196600447772E-04
 1.13670464775796E-04

 0
 0
 0
 0
 3.00606379431009E-04
 2.98361144854336E-04
 1.17188644420356E-04
 0
 2.42403674839711E-05
 1.27451042868158E-05
 0
 0
 0
 0
 0
 0
 4.46419512591059E-04

 0
 0
 0
 0
 3.00606379431009E-04
 2.98361144854336E-04
 1.17188644420356E-04
 0
 2.42403674839711E-05
 1.27451042868158E-05
 0
 0
 0
 0
 0
 0
 4.46419512591059E-04

 0
 0
 0
 0
 2.34848733930475E-05
 0
 0
 0
 0
 0
 0
 0
 0
 0
 0
 1.44230769230769E-04
 1.69098300223886E-04

 0
 0
 0
 0
 0
 0
 0
 0
 0
 0
 0
 0
 0
 0
 0
 1.44230769230769E-04
 4.05835920537327E-05

 0
 0
 0
 0
 2.34848733930475E-05
 0
 0
 0
 0
 0
 0
 0
 0
 0
 0
 0
 1.28514708170153E-04

 0
 0
 5.39578049964927E-05
 0
 0
 8.52460413869531E-05
 0
 0
 3.63605512259566E-05

 0
 0
 5.39578049964927E-05
 0
 0
 8.52460413869531E-05
 0
 0
 3.63605512259566E-05

 0
 0
 0
 0
 0
 0
 0
 0
 0
 0
 0
 0
 0
 2.78951699513229E-05

 0
 0
 0
 0
 0
 0
 0
 0
 0
 0
 0
 0
 0
 2.78951699513229E-05

 0
 0
 1.41369449090811E-02
 7.98664814183728E-03
 3.7575797428876E-04
 1.34262515184451E-02
 4.42387132686843E-03
 1.29859288185587E-04
 1.90711091180142E-02
 8.28431778643029E-05
 8.26910640643718E-03
 9.7018119560565E-04
 4.07446785737222E-03
 2.51056529561907E-04
 4.78737647850252E-03
 1.44230769230769E-03
 6.93303030917933E-03
 6.21550101394054E-03

 0
 0
 7.89582546448677E-03
 4.1855628664552E-03
 6.57576455005331E-05
 6.39345310402148E-03
 2.92971611050889E-04
 0
 1.27867938477947E-02
 0
 0
 0
 0
 0
 2.61248375361666E-05
 0
 1.46777324594333E-03

 0
 0
 7.89582546448677E-03
 4.1855628664552E-03
 6.57576455005331E-05
 6.39345310402148E-03
 2.92971611050889E-04
 0
 1.27867938477947E-02
 0
 0
 0
 0
 0
 2.61248375361666E-05
 0
 1.46777324594333E-03

 0
 0
 4.96411805967733E-03
 1.18838528149876E-03
 2.11363860537428E-04
 4.24099055900091E-03
 1.80909969823924E-03
 5.56539806509661E-05
 3.73301659253154E-03
 8.28431778643029E-05
 2.70336170979677E-04
 4.6470863991195E-04
 3.09688099842302E-03
 1.39475849756615E-04
 1.63933355539445E-03
 9.61538461538462E-04
 2.15093037884783E-03
 5.56985277401402E-03

 0
 0
 3.59718699976618E-04
 1.10974213787017E-03
 3.28788227502666E-05
 9.37706455256484E-04
 1.64064102188498E-03
 5.56539806509661E-05
 4.24206430969493E-04
 8.28431778643029E-05
 2.27930497100512E-04
 4.6470863991195E-04
 3.09688099842302E-03
 1.39475849756615E-04
 1.63933355539445E-03
 9.61538461538462E-04
 1.47453717795229E-03
 5.56985277401402E-03

 0
 0
 4.60439935970071E-03
 7.86431436285947E-05
 1.78485037787161E-04
 3.30328410374443E-03
 1.68458676354261E-04
 0
 3.30881016156205E-03
 0
 4.2405673879165E-05
 0
 0
 0
 0
 0
 6.76393200895545E-04

 0
 0
 .001277001384917
 2.61269999388331E-03
 9.86364682507997E-05
 2.79180785542272E-03
 2.26320569536812E-03
 7.42053075346214E-05
 2.0058904092986E-03
 0
 7.1559574671091E-03
 5.054725556937E-04
 9.77586858949201E-04
 1.11580679805292E-04
 3.12191808557191E-03
 4.80769230769231E-04
 3.2940348883613E-03
 6.45648239926523E-04

 0
 0
 1.16908577492401E-03
 2.49910434197534E-03
 9.86364682507997E-05
 2.74918483472924E-03
 2.26320569536812E-03
 0
 1.59986425394209E-03
 0
 5.01977164544616E-03
 6.9298656828975E-05
 9.27637165426249E-05
 3.71935599350972E-05
 5.87808844563748E-05
 4.80769230769231E-04
 3.14522838416428E-03

 0
 0
 1.07915609992985E-04
 1.1359565190797E-04
 0
 4.26230206934765E-05
 0
 7.42053075346214E-05
 4.06026155356515E-04
 0
 2.13618582166294E-03
 4.36173898864725E-04
 8.84823142406576E-04
 7.43871198701945E-05
 3.06313720111553E-03
 0
 1.4880650419702E-04
 6.45648239926523E-04

 0
 0
 0
 0
 0
 0
 0
 0
 4.96927533421407E-04
 0
 7.36798583650492E-04

 0
 0
 0
 0
 0
 0
 0
 0
 4.96927533421407E-04
 0
 7.36798583650492E-04

 0
 0
 0
 0
 0
 0
 5.85943222101778E-05
 0
 0
 0
 1.06014184697913E-04

 0
 0
 0
 0
 0
 0
 5.85943222101778E-05
 0
 0
 0
 1.06014184697913E-04

 0
 0
 0
 0
 0
 0
 0
 0
 4.84807349679421E-05
 0
 0
 0
 0
 0
 0
 0
 2.02917960268663E-05

 0
 0
 0
 0
 0
 0
 0
 0
 4.84807349679421E-05
 0
 0
 0
 0
 0
 0
 0
 2.02917960268663E-05

 0
 0
 1.43887479990647E-03
 4.13313410403614E-03
 2.62091187066411E-03
 2.70656181403576E-03
 7.00202150411625E-03
 0
 1.89074866374974E-03
 0
 5.4597305119425E-04
 0
 0
 0
 0
 .010400641025641
 9.34775403637643E-03

 0
 0
 1.43887479990647E-03
 4.13313410403614E-03
 2.62091187066411E-03
 2.70656181403576E-03
 7.00202150411625E-03
 0
 1.89074866374974E-03
 0
 5.4597305119425E-04
 0
 0
 0
 0
 .010400641025641
 9.34775403637643E-03

 0
 0
 1.43887479990647E-03
 4.13313410403614E-03
 2.62091187066411E-03
 2.70656181403576E-03
 7.00202150411625E-03
 0
 1.89074866374974E-03
 0
 5.4597305119425E-04
 0
 0
 0
 0
 .010400641025641
 9.34775403637643E-03

 0
 0
 1.61873414989478E-04
 0
 0
 6.39345310402148E-05
 0
 0
 8.39322724132498E-03
 0
 1.54356652920161E-02

 0
 0
 1.61873414989478E-04
 0
 0
 0
 0
 0
 7.16908868338444E-03
 0
 1.11261886840459E-02

 0
 0
 1.61873414989478E-04
 0
 0
 0
 0
 0
 6.39339692389737E-03
 0
 8.77797449298716E-03

 0
 0
 0
 0
 0
 0
 0
 0
 7.75691759487074E-04
 0
 2.29520709870981E-03

 0
 0
 0
 0
 0
 0
 0
 0
 0
 0
 5.30070923489563E-05

 0
 0
 0
 0
 0
 6.39345310402148E-05
 0
 0
 1.22413855794054E-03
 0
 4.27237164332588E-03

 0
 0
 0
 0
 0
 6.39345310402148E-05
 0
 0
 1.22413855794054E-03
 0
 4.27237164332588E-03

 0
 0
 0
 0
 0
 0
 0
 0
 0
 0
 3.71049646442694E-05

 0
 0
 0
 0
 0
 0
 0
 0
 0
 0
 3.71049646442694E-05

 0
 0
 3.27344016978723E-03
 3.49525082793754E-04
 1.45606215036895E-04
 5.47705815911174E-03
 9.37509155362845E-04
 3.24648220463969E-05
 1.55744361084514E-03
 0
 0
 3.6687524203575E-05
 1.49849080568856E-04
 9.29838998377431E-05
 0
 3.0448717948718E-04
 3.87573304113147E-03
 7.72959160475415E-05

 0
 0
 3.27344016978723E-03
 3.49525082793754E-04
 1.45606215036895E-04
 5.47705815911174E-03
 9.37509155362845E-04
 3.24648220463969E-05
 1.55744361084514E-03
 0
 0
 3.6687524203575E-05
 1.49849080568856E-04
 9.29838998377431E-05
 0
 3.0448717948718E-04
 3.87573304113147E-03
 7.72959160475415E-05

 0
 0
 3.27344016978723E-03
 3.49525082793754E-04
 1.45606215036895E-04
 5.47705815911174E-03
 9.37509155362845E-04
 3.24648220463969E-05
 1.55744361084514E-03
 0
 0
 3.6687524203575E-05
 1.49849080568856E-04
 9.29838998377431E-05
 0
 3.0448717948718E-04
 3.87573304113147E-03
 7.72959160475415E-05

 0
 0
 2.84177772981528E-03
 8.56336452844697E-04
 1.98212331437321E-03
 3.38853014513139E-03
 2.23390853426303E-03
 0
 8.90833505035936E-04
 0
 6.36085108187475E-05
 3.26111326254E-05
 2.06934444595086E-04
 0
 1.56749025216999E-04
 1.20192307692308E-03
 4.53183444600015E-03
 .001141251466349

 0
 0
 2.84177772981528E-03
 8.56336452844697E-04
 1.98212331437321E-03
 3.38853014513139E-03
 2.23390853426303E-03
 0
 8.60533045680972E-04
 0
 0
 3.26111326254E-05
 2.06934444595086E-04
 0
 1.56749025216999E-04
 1.20192307692308E-03
 4.53183444600015E-03
 .001141251466349

 0
 0
 2.84177772981528E-03
 8.56336452844697E-04
 1.98212331437321E-03
 3.38853014513139E-03
 2.23390853426303E-03
 0
 8.60533045680972E-04
 0
 0
 3.26111326254E-05
 2.06934444595086E-04
 0
 1.56749025216999E-04
 1.20192307692308E-03
 4.53183444600015E-03
 .001141251466349

 0
 0
 0
 0
 0
 0
 0
 0
 3.03004593549638E-05
 0
 6.36085108187475E-05

 0
 0
 0
 0
 0
 0
 0
 0
 0
 0
 3.71049646442694E-05

 0
 0
 0
 0
 0
 0
 0
 0
 3.03004593549638E-05
 0
 2.65035461744781E-05

 0
 0
 0
 6.11668894889069E-05
 0
 0
 0
 1.39134951627415E-05
 4.18146339098501E-04
 0
 8.48113477583301E-04
 4.076391578175E-05
 0
 0
 0
 0
 4.05835920537327E-05

 0
 0
 0
 6.11668894889069E-05
 0
 0
 0
 1.39134951627415E-05
 4.18146339098501E-04
 0
 8.48113477583301E-04
 4.076391578175E-05
 0
 0
 0
 0
 4.05835920537327E-05

 0
 0
 0
 6.11668894889069E-05
 0
 0
 0
 1.39134951627415E-05
 4.18146339098501E-04
 0
 8.48113477583301E-04
 4.076391578175E-05
 0
 0
 0
 0
 4.05835920537327E-05

 0
 0
 8.99296749941546E-05
 1.31071906047658E-04
 4.22727721074855E-05
 1.70492082773906E-04
 1.68458676354262E-04
 2.31891586045692E-05
 4.06026155356516E-04
 0
 1.16615603167704E-04
 0
 4.99496935229519E-05
 0
 3.91872563042499E-04
 0
 2.9761300839404E-04

 0
 0
 8.99296749941546E-05
 1.31071906047658E-04
 2.34848733930475E-05
 1.70492082773906E-04
 5.12700319339056E-05
 2.31891586045692E-05
 2.60583950452689E-04
 0
 5.30070923489563E-05
 0
 4.99496935229519E-05
 0
 3.91872563042499E-04
 0
 2.50265484331352E-04

 0
 0
 8.99296749941546E-05
 1.31071906047658E-04
 2.34848733930475E-05
 1.70492082773906E-04
 5.12700319339056E-05
 2.31891586045692E-05
 2.60583950452689E-04
 0
 5.30070923489563E-05
 0
 4.99496935229519E-05
 0
 3.91872563042499E-04
 0
 2.50265484331352E-04

 0
 0
 0
 0
 0
 0
 1.17188644420356E-04
 0
 3.03004593549638E-05
 0
 6.36085108187475E-05
 0
 0
 0
 0
 0
 4.73475240626881E-05

 0
 0
 0
 0
 0
 0
 1.17188644420356E-04
 0
 0
 0
 0
 0
 0
 0
 0
 0
 4.73475240626881E-05

 0
 0
 0
 0
 0
 0
 0
 0
 0
 0
 6.36085108187475E-05

 0
 0
 0
 0
 0
 0
 0
 0
 3.03004593549638E-05

 0
 0
 0
 0
 1.8787898714438E-05
 0
 0
 0
 1.15141745548863E-04

 0
 0
 0
 0
 1.8787898714438E-05
 0
 0
 0
 1.15141745548863E-04

 0
 0
 1.07915609992985E-04
 0
 2.8651545539518E-04
 0
 9.5215773591539E-05
 0
 4.84807349679421E-05
 0
 0
 0
 0
 0
 0
 1.44230769230769E-04
 1.01458980134332E-04

 0
 0
 1.07915609992985E-04
 0
 2.8651545539518E-04
 0
 9.5215773591539E-05
 0
 4.84807349679421E-05
 0
 0
 0
 0
 0
 0
 1.44230769230769E-04
 1.01458980134332E-04

 0
 0
 1.07915609992985E-04
 0
 2.8651545539518E-04
 0
 9.5215773591539E-05
 0
 4.84807349679421E-05
 0
 0
 0
 0
 0
 0
 1.44230769230769E-04
 1.01458980134332E-04

 0
 0
 2.51803089983633E-04
 0
 1.83182012465771E-04
 2.55738124160859E-04
 6.59186124864501E-05
 1.2058362474376E-04
 1.03021561806877E-04
 1.59313803585198E-04
 1.90825532456243E-04
 0
 6.42210345295096E-05
 2.60354919545681E-04
 1.95936281521249E-05
 0
 1.89390096250752E-04
 1.54591832095083E-04

 0
 0
 2.51803089983633E-04
 0
 1.50303189715504E-04
 2.55738124160859E-04
 6.59186124864501E-05
 1.2058362474376E-04
 1.03021561806877E-04
 1.59313803585198E-04
 1.90825532456243E-04
 0
 6.42210345295096E-05
 2.60354919545681E-04
 1.95936281521249E-05
 0
 1.89390096250752E-04
 1.54591832095083E-04

 0
 0
 2.51803089983633E-04
 0
 0
 2.55738124160859E-04
 0
 1.2058362474376E-04
 0
 1.59313803585198E-04
 1.90825532456243E-04
 0
 6.42210345295096E-05
 2.60354919545681E-04

 0
 0
 0
 0
 1.50303189715504E-04
 0
 6.59186124864501E-05
 0
 1.03021561806877E-04
 0
 0
 0
 0
 0
 1.95936281521249E-05
 0
 1.89390096250752E-04

 0
 0
 0
 0
 0
 0
 0
 0
 0
 0
 0
 0
 0
 0
 0
 0
 0
 1.54591832095083E-04

 0
 0
 0
 0
 3.28788227502666E-05

 0
 0
 0
 0
 3.28788227502666E-05

 0
 0
 0
 0
 7.04546201791426E-05
 0
 2.19728708288167E-05
 0
 2.24223399226732E-04

 0
 0
 0
 0
 7.04546201791426E-05
 0
 2.19728708288167E-05
 0
 2.24223399226732E-04

 0
 0
 0
 0
 1.40909240358285E-05
 0
 2.19728708288167E-05
 0
 2.24223399226732E-04

 0
 0
 0
 0
 5.63636961433141E-05

 0
 0
 0
 0
 0
 0
 0
 0
 1.0908165367787E-04
 0
 0
 0
 0
 0
 0
 0
 2.02917960268663E-04

 0
 0
 0
 0
 0
 0
 0
 0
 1.0908165367787E-04
 0
 0
 0
 0
 0
 0
 0
 2.02917960268663E-04

 0
 0
 0
 0
 0
 0
 0
 0
 0
 0
 0
 0
 0
 0
 0
 0
 2.02917960268663E-04

 0
 0
 0
 0
 0
 0
 0
 0
 1.0908165367787E-04

 0
 0
 0
 0
 2.8181848071657E-05
 0
 0
 0
 0
 0
 0
 0
 0
 0
 1.82873862753166E-04

 0
 0
 0
 0
 2.8181848071657E-05
 0
 0
 0
 0
 0
 0
 0
 0
 0
 1.82873862753166E-04

 0
 0
 0
 0
 2.8181848071657E-05
 0
 0
 0
 0
 0
 0
 0
 0
 0
 1.82873862753166E-04

 0
 0
 0
 0
 0
 0
 0
 0
 1.0908165367787E-04
 0
 1.64321986281764E-04

 0
 0
 0
 0
 0
 0
 0
 0
 1.0908165367787E-04
 0
 1.64321986281764E-04

 0
 0
 0
 0
 0
 0
 0
 0
 1.0908165367787E-04
 0
 1.64321986281764E-04

 0
 0
 0
 0
 0
 0
 0
 0
 0
 0
 1.37818440107286E-04

 0
 0
 0
 0
 0
 0
 0
 0
 0
 0
 1.37818440107286E-04

 0
 0
 0
 0
 0
 0
 0
 0
 0
 0
 1.37818440107286E-04

 0
 0
 0
 0
 0
 0
 0
 0
 8.48412861938987E-05
 0
 0
 0
 0
 0
 0
 0
 8.79311161164208E-05

 0
 0
 0
 0
 0
 0
 0
 0
 8.48412861938987E-05
 0
 0
 0
 0
 0
 0
 0
 8.79311161164208E-05

 0
 0
 0
 0
 0
 0
 0
 0
 8.48412861938987E-05
 0
 0
 0
 0
 0
 0
 0
 8.79311161164208E-05

 0
 0
 0
 0
 0
 0
 0
 0
 0
 0
 2.65035461744781E-05

 0
 0
 0
 0
 0
 0
 0
 0
 0
 0
 2.65035461744781E-05

 0
 0
 0
 0
 0
 0
 0
 0
 0
 0
 2.65035461744781E-05

 0
 0
 0
 0
 0
 0
 0
 1.85513268836554E-05

 0
 0
 0
 0
 0
 0
 0
 1.85513268836554E-05

 0
 0
 0
 0
 0
 0
 0
 1.85513268836554E-05

 0
 0
 0
 1.05731337545111E-03
 3.28788227502666E-05
 0
 2.19728708288167E-05
 0
 0
 0
 1.06014184697913E-05

 0
 0
 0
 1.05731337545111E-03
 3.28788227502666E-05
 0
 2.19728708288167E-05
 0
 0
 0
 1.06014184697913E-05

 0
 0
 0
 1.05731337545111E-03
 3.28788227502666E-05
 0
 2.19728708288167E-05
 0
 0
 0
 1.06014184697913E-05

 0
 0
 0
 1.05731337545111E-03
 3.28788227502666E-05
 0
 2.19728708288167E-05
 0
 0
 0
 1.06014184697913E-05

 .125
 0
 4.13676504973111E-04
 1.39810033117502E-04
 1.48095611616558E-02
 2.34426613814121E-04
 5.85943222101779E-04
 1.66961941952898E-04
 3.47243264207886E-03
 3.12255055026988E-04
 1.52130355041505E-03
 1.793612294397E-04
 1.8552743308525E-04
 1.16229874797179E-04
 1.63280234601041E-04
 2.56410256410256E-04
 1.06193732540601E-03
 2.72809115461911E-05

 .125
 0
 1.43887479990647E-04
 9.61193977682823E-05
 2.8651545539518E-03
 2.34426613814121E-04
 1.318372249729E-04
 1.66961941952898E-04
 2.24223399226733E-04
 3.12255055026988E-04
 1.52130355041505E-03
 1.793612294397E-04
 1.8552743308525E-04
 1.16229874797179E-04
 1.63280234601041E-04
 0
 3.38196600447772E-05
 2.72809115461911E-05

 .125

 .125

 .125

 0
 0
 1.43887479990647E-04
 9.61193977682823E-05
 2.83697270588014E-03
 0
 1.318372249729E-04
 1.66961941952898E-04
 1.87862848000776E-04
 3.12255055026988E-04
 1.52130355041505E-03
 1.793612294397E-04
 1.8552743308525E-04
 1.16229874797179E-04
 1.63280234601041E-04
 0
 3.38196600447772E-05
 2.72809115461911E-05

 0
 0
 0
 9.61193977682823E-05
 1.62515323879889E-03
 0
 1.318372249729E-04
 1.66961941952898E-04
 1.87862848000776E-04
 3.12255055026988E-04
 1.39938723801245E-03
 1.141389641889E-04
 7.84923755360672E-05
 1.16229874797179E-04
 7.83745126084997E-05
 0
 0
 2.72809115461911E-05

 0
 0
 0
 9.61193977682823E-05
 9.20607037007464E-04
 0
 1.318372249729E-04
 1.66961941952898E-04
 1.87862848000776E-04
 3.12255055026988E-04
 1.24036596096558E-03
 1.141389641889E-04
 7.84923755360672E-05
 1.16229874797179E-04
 0
 0
 0
 2.72809115461911E-05

 0
 0
 0
 0
 7.04546201791426E-04
 0
 0
 0
 0
 0
 1.59021277046869E-04
 0
 0
 0
 7.83745126084997E-05

 0
 0
 1.43887479990647E-04
 0
 1.21181946708125E-03
 0
 0
 0
 0
 0
 1.21916312402599E-04
 6.52222652508E-05
 1.07035057549183E-04
 0
 8.49057219925414E-05
 0
 3.38196600447772E-05

 0
 0
 0
 0
 5.58939986754531E-04
 0
 0
 0
 0
 0
 1.21916312402599E-04
 6.52222652508E-05
 1.07035057549183E-04
 0
 8.49057219925414E-05
 0
 3.38196600447772E-05

 0
 0
 1.43887479990647E-04
 0
 4.69697467860951E-04

 0
 0
 0
 0
 1.83182012465771E-04

 0
 0
 0
 0
 0
 2.34426613814121E-04
 0
 0
 3.63605512259566E-05

 0
 0
 0
 0
 0
 2.34426613814121E-04
 0
 0
 3.63605512259566E-05

 0
 0
 0
 0
 0
 2.34426613814121E-04
 0
 0
 3.63605512259566E-05

 0
 0
 0
 0
 2.8181848071657E-05

 0
 0
 0
 0
 2.8181848071657E-05

 0
 0
 0
 0
 2.8181848071657E-05

 0
 0
 2.69789024982464E-04
 4.36906353492192E-05
 1.19350126583468E-02
 0
 4.54105997128879E-04
 0
 3.24820924285212E-03
 0
 0
 0
 0
 0
 0
 2.56410256410256E-04
 1.02811766536123E-03

 0
 0
 2.15831219985971E-04
 0
 5.90409717101215E-03
 0
 2.12404418011895E-04
 0
 1.98771013368563E-03
 0
 0
 0
 0
 0
 0
 1.92307692307692E-04
 6.76393200895545E-04

 0
 0
 2.15831219985971E-04
 0
 5.90409717101215E-03
 0
 2.12404418011895E-04
 0
 1.98771013368563E-03
 0
 0
 0
 0
 0
 0
 1.92307692307692E-04
 6.76393200895545E-04

 0
 0
 2.15831219985971E-04
 0
 5.77258188001108E-03
 0
 2.12404418011895E-04
 0
 1.98771013368563E-03
 0
 0
 0
 0
 0
 0
 1.92307692307692E-04
 6.76393200895545E-04

 0
 0
 0
 0
 7.51515948577521E-05

 0
 0
 0
 0
 5.63636961433141E-05

 0
 0
 0
 0
 2.91682127541651E-03
 0
 1.24512934696628E-04
 0
 3.21184869162616E-04
 0
 0
 0
 0
 0
 0
 0
 1.4880650419702E-04

 0
 0
 0
 0
 1.45136517569034E-03
 0
 1.24512934696628E-04
 0
 2.06043123613754E-04
 0
 0
 0
 0
 0
 0
 0
 3.38196600447772E-05

 0
 0
 0
 0
 1.45136517569034E-03
 0
 1.24512934696628E-04
 0
 2.06043123613754E-04
 0
 0
 0
 0
 0
 0
 0
 3.38196600447772E-05

 0
 0
 0
 0
 1.46545609972617E-03
 0
 0
 0
 1.15141745548863E-04
 0
 0
 0
 0
 0
 0
 0
 1.14986844152243E-04

 0
 0
 0
 0
 8.45455442149711E-04
 0
 0
 0
 6.06009187099276E-05
 0
 0
 0
 0
 0
 0
 0
 1.14986844152243E-04

 0
 0
 0
 0
 6.20000657576455E-04
 0
 0
 0
 5.45408268389349E-05

 0
 0
 0
 2.62143812095315E-05
 1.16954669497377E-03
 0
 0
 0
 2.42403674839711E-04
 0
 0
 0
 0
 0
 0
 6.41025641025641E-05
 6.76393200895545E-05

 0
 0
 0
 2.62143812095315E-05
 1.16954669497377E-03
 0
 0
 0
 2.42403674839711E-04
 0
 0
 0
 0
 0
 0
 6.41025641025641E-05
 6.76393200895545E-05

 0
 0
 0
 2.62143812095315E-05
 1.16954669497377E-03
 0
 0
 0
 2.42403674839711E-04
 0
 0
 0
 0
 0
 0
 6.41025641025641E-05
 6.76393200895545E-05

 0
 0
 0
 0
 1.85060802337215E-03
 0
 1.17188644420356E-04
 0
 6.96910565164167E-04
 0
 0
 0
 0
 0
 0
 0
 1.08222912143287E-04

 0
 0
 0
 0
 8.92425188935806E-04
 0
 1.17188644420356E-04
 0
 1.69682572387797E-04
 0
 0
 0
 0
 0
 0
 0
 1.08222912143287E-04

 0
 0
 0
 0
 8.92425188935806E-04
 0
 1.17188644420356E-04
 0
 1.69682572387797E-04
 0
 0
 0
 0
 0
 0
 0
 1.08222912143287E-04

 0
 0
 0
 0
 5.82424860147579E-04

 0
 0
 0
 0
 5.82424860147579E-04

 0
 0
 0
 0
 0
 0
 0
 0
 5.02987625292399E-04

 0
 0
 0
 0
 0
 0
 0
 0
 5.02987625292399E-04

 0
 0
 0
 0
 2.01969911180209E-04
 0
 0
 0
 2.42403674839711E-05

 0
 0
 0
 0
 1.69091088429942E-04
 0
 0
 0
 2.42403674839711E-05

 0
 0
 0
 0
 3.28788227502666E-05

 0
 0
 0
 0
 1.12727392286628E-04

 0
 0
 0
 0
 1.12727392286628E-04

 0
 0
 0
 0
 6.10606708219236E-05

 0
 0
 0
 0
 6.10606708219236E-05

 0
 0
 5.39578049964927E-05
 1.74762541396877E-05
 6.10606708219236E-05
 0
 0
 0
 0
 0
 0
 0
 0
 0
 0
 0
 2.70557280358218E-05

 0
 0
 0
 1.74762541396877E-05
 6.10606708219236E-05
 0
 0
 0
 0
 0
 0
 0
 0
 0
 0
 0
 2.70557280358218E-05

 0
 0
 0
 1.74762541396877E-05
 6.10606708219236E-05
 0
 0
 0
 0
 0
 0
 0
 0
 0
 0
 0
 2.70557280358218E-05

 0
 0
 5.39578049964927E-05

 0
 0
 5.39578049964927E-05

 0
 0
 0
 0
 3.28788227502666E-05

 0
 0
 0
 0
 3.28788227502666E-05

 0
 0
 0
 0
 3.28788227502666E-05

 0
 0
 0
 0
 9.39394935721902E-06

 0
 0
 0
 0
 9.39394935721902E-06

 0
 0
 0
 0
 9.39394935721902E-06

 0
 0
 0
 0
 9.39394935721902E-06

 0
 0
 4.46590766020972E-02
 5.87202139093506E-02
 2.48751778979159E-02
 5.62410758050422E-02
 .141410072363988
 .0816072869612
 .097197813518853
 5.82260089343181E-02
 1.29867376254943E-02
 .172908301571449
 .125466494459152
 .177789865684757
 .084056664772616
 .198605769230769
 .101716009550672
 .133594623841698

 0
 0
 4.41734563571287E-02
 5.84930226055347E-02
 2.41424498480529E-02
 5.60279607015749E-02
 .139688864149064
 8.15887356343163E-02
 9.61797180845262E-02
 5.82260089343181E-02
 .011783476629173
 .172908301571449
 .125466494459152
 .177789865684757
 8.39521654224713E-02
 .197692307692308
 .100728475477364
 .133540062018606

 0
 0
 2.52882246083563E-02
 5.49191286339686E-02
 1.11834967097692E-02
 3.24148072373889E-02
 .103323762927372
 8.13846710385961E-02
 7.51511992921813E-02
 5.81431657564538E-02
 1.09618666977642E-02
 .161050078470538
 9.52183871957528E-02
 .176655462106736
 7.25290802097825E-02
 .140352564102564
 .047523386294921
 9.81021579201034E-02

 0
 0
 1.59355384089642E-02
 5.17471885076152E-02
 5.67864238643889E-03
 2.06508535259894E-02
 9.66147130343069E-02
 8.13846710385961E-02
 6.82426945592496E-02
 5.81431657564538E-02
 1.06385234344355E-02
 .160915557548458
 9.48830106820987E-02
 .176055715952783
 7.15298051740241E-02
 .128669871794872
 2.47627550847859E-02
 9.76520228795912E-02

 0
 0
 1.13311390492635E-03
 4.89684640994049E-02
 2.16060835216037E-04
 3.92131790379984E-03
 8.70565142237717E-02
 0
 3.69665604130559E-04
 0
 0
 2.73118235737725E-04
 1.71256092078692E-04
 6.50887298864202E-05
 0
 .105320512820513
 7.02772535730471E-03
 6.00180054016205E-04

 0
 0
 1.27700138491699E-03
 4.10691972282661E-04
 3.61667050252932E-04
 .002280331607101
 5.34673190167873E-04
 3.35083341836025E-02
 3.17548814040021E-03
 2.31897172498614E-02
 3.85361561376912E-03
 2.05613191203147E-02
 5.50873762853126E-03
 8.63262526093607E-02
 2.35123537825499E-04
 2.56410256410256E-03
 1.87360916648066E-03
 2.0415215473733E-03

 0
 0
 8.27353009946222E-04
 0
 7.51515948577521E-05
 1.55574025531189E-03
 6.51861834588228E-04
 4.37904071088685E-02
 6.04797168725078E-03
 .034010310789368
 3.53027235044049E-03
 5.10771864745327E-02
 2.30910297486103E-02
 6.44285441975722E-02
 9.05225620628172E-03
 0
 2.77321212367173E-03
 5.13335818927496E-03

 0
 0
 0
 0
 0
 0
 0
 0
 7.87811943229059E-05
 0
 0
 0
 0
 0
 5.42155690969297E-02
 0
 5.41114560716436E-05
 3.63745487282548E-05

 0
 0
 7.9138113994856E-04
 1.92238795536565E-04
 0
 1.12951004837713E-03
 3.22268772155978E-04
 2.13340259162037E-03
 3.9390597161453E-04
 4.01470785034699E-04
 9.01120569932257E-05
 4.49952102398956E-02
 2.34620846147808E-02
 1.75321143144065E-02
 .001097243176519
 5.76923076923077E-04
 3.92308056519416E-04
 2.68125892313149E-02

 0
 0
 0
 0
 0
 0
 0
 0
 .035209133770468
 0
 3.23343263328633E-04
 0
 0
 0
 2.44267230963157E-03

 0
 0
 1.25901544991816E-04
 6.72835784377976E-04
 1.05681930268714E-03
 3.62295675894551E-04
 2.2412328245393E-03
 6.02918123718799E-05
 3.09064685420631E-04
 0
 0
 1.27305708986405E-02
 1.34792815806937E-02
 1.1204559930448E-03
 .001097243176519
 2.51602564102564E-03
 4.66711308617926E-04
 2.65579673902171E-02

 0
 0
 1.07915609992985E-04
 5.24287624190631E-05
 1.73788063108552E-04
 1.49180572427168E-04
 2.78323030498345E-04
 3.89577864556762E-04
 1.27261929290848E-04
 9.55882821511187E-05
 3.71049646442694E-05
 2.59421560035057E-02
 2.33336425457218E-02
 2.85460572501871E-03
 2.12264304981353E-03
 0
 1.75862232232842E-04
 2.42390899087908E-02

 0
 0
 1.25901544991816E-04
 1.48548160187345E-04
 0
 3.19672655201074E-04
 5.12700319339056E-04
 0
 4.66627074066443E-04
 0
 0
 0
 0
 0
 0
 1.19391025641026E-02
 1.40689785786273E-03

 0
 0
 1.11512796992752E-03
 6.99050165587508E-05
 7.04546201791426E-05
 5.11476248321719E-04
 0
 0
 7.09636758093253E-03
 0
 0
 5.2993090516275E-05
 5.06632605732798E-04
 0
 1.37155397064874E-04
 0
 2.29973688304485E-04
 7.18397337383033E-04

 0
 0
 2.93170740480944E-03
 0
 9.39394935721902E-05
 1.06557551733691E-03
 0
 0
 5.04805652853697E-03
 0
 0
 0
 0
 0
 0
 0
 1.35278640179109E-04

 0
 0
 1.70866382488894E-03
 0
 5.63636961433141E-05
 3.21803806235748E-03
 2.92971611050889E-05
 0
 3.99966063485522E-03
 0
 1.59021277046869E-05
 0
 0
 0
 0
 0
 3.11817265612846E-03

 0
 0
 1.61873414989478E-04
 1.66024414327033E-04
 7.51515948577521E-05
 2.98361144854336E-04
 4.02835965194973E-04
 1.39134951627415E-05
 4.24206430969493E-05
 0
 0
 1.956667957524E-04
 1.60552586323774E-03
 4.64919499188715E-05
 4.31059819346748E-04
 3.0448717948718E-04
 1.28514708170153E-04
 3.72384442605509E-03

 0
 0
 3.4353135847767E-03
 8.21383944565322E-04
 1.19772854304542E-03
 2.81311936576945E-03
 2.21925995371049E-03
 0
 1.66046517265202E-03
 0
 0
 3.26111326254E-05
 0
 0
 0
 1.98717948717949E-03
 2.89496289983293E-03
 3.63745487282548E-05

 0
 0
 0
 0
 7.04546201791426E-05
 0
 8.05671930389945E-05
 0
 3.63605512259566E-05
 0
 0
 7.05215743024275E-04
 8.6341613089674E-04
 0
 2.28592328441457E-04
 0
 0
 2.80993388925769E-03

 0
 0
 0
 0
 0
 0
 0
 9.41479839345509E-04
 0
 2.61274637879724E-04
 4.77063831140607E-05
 2.48659886268675E-04
 0
 2.42223059077321E-03
 0
 0
 2.02917960268663E-05

 0
 0
 0
 0
 0
 0
 0
 0
 2.42403674839711E-05
 0
 0
 2.3643071153415E-04
 6.49346015798375E-04
 0
 5.87808844563748E-05
 0
 0
 1.83236789218584E-03

 0
 0
 0
 0
 0
 0
 0
 1.43772783348329E-04
 1.80590737755584E-03
 0
 1.07074326544892E-03
 2.0381957890875E-05
 0
 0
 0
 0
 1.41366178987169E-03

 0
 0
 9.71240489936869E-04
 1.66024414327033E-04
 6.95152252434207E-04
 1.12951004837713E-03
 6.15240383206867E-04
 0
 3.87845879743537E-04
 0
 0
 0
 0
 0
 0
 1.37820512820513E-03
 7.5756038500301E-04

 0
 0
 5.39578049964927E-05
 0
 0
 0
 0
 0
 2.66644042323682E-04
 0
 1.18205815938173E-03

 0
 0
 0
 0
 8.92425188935806E-05
 3.40984165547812E-04
 6.44537544311956E-04
 0
 0
 0
 0
 1.03540346085645E-03
 3.56783525163942E-04
 2.09213774634922E-04
 0
 9.93589743589744E-04
 9.46950481253762E-04
 3.36464575736357E-04

 0
 0
 4.67634309969604E-04
 0
 1.69091088429942E-04
 8.95083434563008E-04
 1.83107256906806E-04
 1.48410615069243E-04
 5.57528452131334E-04
 8.92157300077108E-05
 0
 2.771946273159E-04
 0
 2.92899284488891E-04
 0
 5.12820512820513E-04
 3.7201626049255E-04

 0
 0
 2.33817154984802E-04
 0
 2.34848733930475E-05
 5.32787758668457E-04
 0
 6.49296440927937E-05
 1.27261929290848E-04
 0
 0
 7.500560503842E-04
 2.49748467614759E-04
 2.04564579643035E-04
 0
 0
 2.77321212367173E-04
 6.82022788654778E-05

 0
 0
 0
 0
 0
 0
 0
 0
 0
 0
 0
 .000163055663127
 2.78291149627875E-04
 0
 5.22496750723331E-05
 0
 0
 6.91116425836842E-04

 0
 0
 0
 0
 0
 0
 0
 0
 0
 0
 0
 5.01396164115525E-04
 4.78089923719682E-04
 0
 3.5921651612229E-04
 0
 0
 6.138205097893E-04

 0
 0
 0
 0
 5.58939986754531E-04

 0
 0
 4.67634309969604E-04
 0
 0
 1.2786906208043E-04
 0
 0
 2.8482431793666E-04

 0
 0
 0
 0
 1.12727392286628E-04
 0
 4.46781706852606E-04
 0
 0
 0
 0
 0
 0
 0
 0
 4.16666666666667E-04
 2.29973688304485E-04

 0
 0
 0
 0
 3.75757974288761E-05
 0
 0
 0
 0
 0
 0
 1.5490287997065E-04
 2.28341456104923E-04
 0
 0
 0
 0
 3.63745487282548E-04

 0
 0
 0
 0
 0
 0
 0
 0
 0
 0
 0
 1.2229174734525E-04
 8.56280460393461E-05
 0
 0
 0
 0
 3.36464575736357E-04

 0
 0
 0
 0
 0
 0
 0
 1.06670129581018E-04
 0
 7.6470625720895E-05
 0
 3.30187717832175E-04
 7.84923755360672E-05
 2.32459749594358E-04

 0
 0
 0
 0
 0
 0
 0
 0
 2.24223399226732E-04
 0
 3.23343263328633E-04

 0
 0
 0
 0
 0
 0
 0
 0
 2.66644042323682E-04
 0
 1.53720567811973E-04

 0
 0
 0
 0
 0
 0
 0
 3.71026537673107E-05
 0
 1.91176564302237E-05
 0
 2.56812669425025E-04
 9.27637165426249E-05
 1.53423434732276E-04
 0
 0
 0
 3.1827730137223E-05

 0
 0
 0
 0
 9.39394935721902E-05
 0
 4.39457416576334E-05
 0
 7.27211024519132E-05
 0
 0
 5.2993090516275E-05
 1.2130639855574E-04
 0
 0
 0
 6.76393200895545E-05
 2.22794110960561E-04

 0
 0
 0
 0
 0
 0
 0
 0
 0
 0
 0
 0
 0
 0
 0
 0
 0
 1.9551319941437E-04

 0
 0
 0
 7.86431436285947E-05
 5.16667214647046E-05
 0
 1.46485805525445E-04
 0
 0
 0
 0
 1.67132054705175E-04
 1.49849080568856E-04
 0
 0
 1.6025641025641E-04
 0
 1.68232287868179E-04

 0
 0
 0
 0
 1.36212265679676E-04

 0
 0
 0
 0
 0
 0
 6.59186124864501E-05
 4.63783172091384E-05
 4.84807349679421E-05
 0
 0
 0
 0
 1.11580679805292E-04

 0
 0
 0
 0
 9.39394935721902E-05

 0
 0
 0
 0
 0
 0
 0
 0
 0
 0
 0
 1.63055663127E-05
 9.27637165426249E-05

 0
 0
 0
 0
 3.28788227502666E-05
 0
 8.05671930389945E-05
 0
 5.45408268389349E-05
 0
 0
 0
 0
 5.57903399026459E-05

 0
 0
 0
 0
 7.04546201791426E-05

 0
 0
 0
 0
 6.57576455005331E-05
 0
 5.85943222101778E-05
 0
 3.63605512259566E-05
 0
 0
 1.63055663127E-05
 0
 0
 0
 0
 0
 4.54681859103186E-05

 0
 0
 0
 0
 0
 0
 0
 0
 0
 0
 1.06014184697913E-05
 0
 0
 0
 0
 0
 0
 3.63745487282548E-05

 0
 0
 0
 0
 0
 0
 0
 0
 2.42403674839711E-05

 0
 0
 3.52524325977086E-03
 6.72835784377976E-04
 2.06666885858819E-03
 4.36885962108135E-03
 1.55274953856971E-03
 0
 3.91481934866133E-03
 0
 2.65035461744781E-04
 1.34520922079775E-04
 3.35376513654105E-04
 5.76500178994007E-04
 9.99275035758371E-04
 6.90705128205128E-03
 1.22494808682183E-02
 4.50135040512154E-04

 0
 0
 1.38491699490998E-03
 2.79620066235003E-04
 3.33485202181275E-04
 2.57869275195533E-03
 1.09131925116456E-03
 0
 1.06657616929473E-03
 0
 2.65035461744781E-04
 1.34520922079775E-04
 4.2814023019673E-05
 5.3930661905891E-04
 1.82873862753166E-04
 4.6474358974359E-03
 9.92945218914659E-03
 6.82022788654778E-05

 0
 0
 2.10435439486322E-03
 3.93215718142973E-04
 1.65803206154916E-03
 1.79016686912602E-03
 4.24808836023789E-04
 0
 2.56341886142994E-03
 0
 0
 0
 2.92562490634432E-04
 0
 7.83745126084997E-04
 2.25961538461538E-03
 2.2320975629553E-03
 3.81932761646676E-04

 0
 0
 3.59718699976618E-05
 0
 0
 0
 0
 0
 1.87862848000776E-04
 0
 0
 0
 0
 0
 3.26560469202082E-05

 0
 0
 0
 0
 4.22727721074856E-05
 0
 0
 0
 9.69614699358842E-05

 0
 0
 0
 0
 0
 0
 3.66214513813611E-05
 0
 0
 0
 0
 0
 0
 3.71935599350972E-05
 0
 0
 8.79311161164208E-05

 0
 0
 0
 0
 3.28788227502666E-05

 0
 0
 4.40655407471357E-03
 4.10691972282661E-04
 3.36773084456301E-03
 5.24263154529761E-03
 2.16066563150031E-03
 0
 1.14535736361763E-03
 0
 1.06014184697913E-05
 0
 0
 0
 0
 3.34935897435897E-03
 6.65570909681216E-03

 0
 0
 3.59718699976618E-03
 0
 1.50303189715504E-04
 4.02787545553353E-03
 7.32429027627223E-05
 0
 5.81768819615305E-04
 0
 0
 0
 0
 0
 0
 2.40384615384615E-04
 3.21963163626279E-03

 0
 0
 8.09367074947391E-04
 4.10691972282661E-04
 2.44712380755555E-03
 1.21475608976408E-03
 2.08742272873759E-03
 0
 3.99966063485522E-04
 0
 1.06014184697913E-05
 0
 0
 0
 0
 3.10897435897436E-03
 3.36167420845086E-03

 0
 0
 0
 0
 7.23334100505864E-04
 0
 0
 0
 7.27211024519132E-05

 0
 0
 0
 0
 4.69697467860951E-05
 0
 0
 0
 0
 0
 0
 0
 0
 0
 0
 0
 7.44032520985099E-05

 0
 0
 0
 0
 0
 0
 0
 0
 6.66610105809204E-05

 0
 0
 0
 0
 0
 0
 0
 0
 2.42403674839711E-05

 0
 0
 1.43887479990647E-04
 1.79131604931799E-03
 0
 2.34426613814121E-04
 1.56007382884598E-03
 0
 2.48463766710703E-04
 0
 0
 0
 0
 0
 0
 1.44230769230769E-04
 9.46950481253762E-05

 0
 0
 1.43887479990647E-04
 1.79131604931799E-03
 0
 2.34426613814121E-04
 1.56007382884598E-03
 0
 2.48463766710703E-04
 0
 0
 0
 0
 0
 0
 1.44230769230769E-04
 9.46950481253762E-05

 0
 0
 5.21592114966097E-04
 0
 4.69697467860951E-05
 1.08688702768365E-03
 2.49025869393256E-04
 0
 2.96944501678645E-04
 0
 0
 0
 0
 0
 0
 0
 1.62334368214931E-03

 0
 0
 5.21592114966097E-04
 0
 4.69697467860951E-05
 1.08688702768365E-03
 2.49025869393256E-04
 0
 2.96944501678645E-04
 0
 0
 0
 0
 0
 0
 0
 1.62334368214931E-03

 0
 0
 3.9569056997428E-04
 2.97096320374691E-04
 0
 3.40984165547812E-04
 9.81454897020479E-04
 0
 1.19383809858557E-03
 0
 4.77063831140607E-05
 0
 0
 2.32459749594358E-05
 0
 5.92948717948718E-04
 9.60478345271673E-04

 0
 0
 3.9569056997428E-04
 2.97096320374691E-04
 0
 3.40984165547812E-04
 9.81454897020479E-04
 0
 1.19383809858557E-03
 0
 4.77063831140607E-05
 0
 0
 2.32459749594358E-05
 0
 5.92948717948718E-04
 9.60478345271673E-04

 0
 0
 3.59718699976618E-04
 0
 2.34848733930475E-05
 4.9016473797498E-04
 2.05080127735622E-04
 0
 1.0908165367787E-04
 0
 0
 0
 0
 0
 0
 6.89102564102564E-04
 1.17692416955825E-03

 0
 0
 3.59718699976618E-04
 0
 2.34848733930475E-05
 4.9016473797498E-04
 2.05080127735622E-04
 0
 1.0908165367787E-04
 0
 0
 0
 0
 0
 0
 6.89102564102564E-04
 1.17692416955825E-03

 0
 0
 1.69967085738952E-02
 1.43305283945439E-03
 7.63258385274044E-03
 2.18229865950599E-02
 2.62942020918173E-02
 0
 1.23322869574703E-02
 1.27451042868158E-05
 5.88378725073415E-04
 9.88524957707437E-03
 2.89066012087826E-02
 1.06931484813405E-04
 1.02474675235613E-02
 4.81410256410256E-02
 4.62450031452283E-02
 3.33008993607173E-02

 0
 0
 1.10973218942787E-02
 6.46621403168445E-04
 6.1295519555854E-03
 1.61328133324808E-02
 1.74318108575279E-02
 0
 8.75683275358455E-03
 1.27451042868158E-05
 5.88378725073415E-04
 9.88524957707437E-03
 2.89066012087826E-02
 1.06931484813405E-04
 1.02278738954092E-02
 4.16185897435897E-02
 3.87708582753326E-02
 3.33008993607173E-02

 0
 0
 2.28421374485153E-03
 1.22333778977814E-04
 1.86939592208658E-03
 3.47377618651834E-03
 6.72369847361791E-03
 0
 1.41806149781231E-03
 0
 5.30070923489563E-05
 9.67735360658745E-03
 .027714944234735
 6.50887298864202E-05
 9.7706892385263E-03
 .017900641025641
 9.35451796838538E-03
 2.78219829585239E-02

 0
 0
 7.32027554452418E-03
 4.10691972282661E-04
 2.65379069341437E-03
 1.09967393389169E-02
 7.83699059561128E-03
 0
 6.17523361654163E-03
 1.27451042868158E-05
 5.03567377315085E-04
 1.91590404174225E-04
 3.49647854660663E-04
 4.18427549269844E-05
 3.91872563042499E-04
 2.13461538461538E-02
 2.63928626989441E-02
 3.63745487282548E-03

 0
 0
 1.24102951491933E-03
 1.1359565190797E-04
 9.34697961043292E-04
 1.10819853803039E-03
 2.59279875780037E-03
 0
 9.75674791229835E-04
 0
 0
 0
 7.9919509636723E-04
 0
 6.53120938404164E-05
 2.37179487179487E-03
 2.66498921152845E-03
 1.74597833895623E-03

 0
 0
 1.9784528498714E-04
 0
 4.69697467860951E-05
 4.68853227628242E-04
 6.59186124864501E-05
 0
 5.45408268389349E-05
 0
 0
 0
 0
 0
 0
 0
 2.36737620313441E-04

 0
 0
 0
 0
 3.47576126217104E-04
 0
 5.85943222101778E-05
 0
 0
 0
 0
 1.63055663127E-05
 4.2814023019673E-05
 0
 0
 0
 0
 9.5483190411669E-05

 0
 0
 5.39578049964927E-05
 0
 2.77121506037961E-04
 8.52460413869531E-05
 1.53810095801717E-04
 0
 1.0908165367787E-04
 0
 0
 0
 0
 0
 0
 0
 1.21750776161198E-04

 0
 0
 0
 0
 0
 0
 0
 0
 0
 0
 3.18042554093738E-05

 0
 0
 0
 0
 0
 0
 0
 0
 2.42403674839711E-05

 0
 0
 5.89938667961654E-03
 7.86431436285946E-04
 1.4842439984406E-03
 5.60492722119217E-03
 8.8623912342894E-03
 0
 3.5148532851758E-03
 0
 0
 0
 0
 0
 1.95936281521249E-05
 6.5224358974359E-03
 7.25769904560919E-03

 0
 0
 5.89938667961654E-03
 7.86431436285946E-04
 1.4842439984406E-03
 5.60492722119217E-03
 8.8623912342894E-03
 0
 3.5148532851758E-03
 0
 0
 0
 0
 0
 1.95936281521249E-05
 6.5224358974359E-03
 7.25769904560919E-03

 0
 0
 0
 0
 0
 8.52460413869531E-05
 0
 0
 0
 0
 0
 0
 0
 0
 0
 0
 2.16445824286574E-04

 0
 0
 0
 0
 0
 8.52460413869531E-05
 0
 0
 0
 0
 0
 0
 0
 0
 0
 0
 2.16445824286574E-04

 0
 0
 0
 0
 1.8787898714438E-05
 0
 0
 0
 6.06009187099276E-05

 0
 0
 0
 0
 1.8787898714438E-05
 0
 0
 0
 6.06009187099276E-05

 0
 0
 5.21592114966097E-04
 2.62143812095315E-05
 1.08030417608019E-03
 6.39345310402148E-04
 5.85943222101778E-05
 0
 7.22362951022338E-03
 0
 2.12028369395825E-05
 0
 0
 1.39475849756615E-05
 2.61248375361666E-05
 1.44230769230769E-04
 3.05729726804786E-03

 0
 0
 5.21592114966097E-04
 2.62143812095315E-05
 1.08030417608019E-03
 6.39345310402148E-04
 5.85943222101778E-05
 0
 7.22362951022338E-03
 0
 2.12028369395825E-05
 0
 0
 1.39475849756615E-05
 2.61248375361666E-05
 1.44230769230769E-04
 3.05729726804786E-03

 0
 0
 5.21592114966097E-04
 2.62143812095315E-05
 1.08030417608019E-03
 6.39345310402148E-04
 5.85943222101778E-05
 0
 7.22362951022338E-03
 0
 2.12028369395825E-05
 0
 0
 1.39475849756615E-05
 2.61248375361666E-05
 1.44230769230769E-04
 3.05729726804786E-03

 0
 0
 1.33095918991349E-03
 2.11462675090222E-03
 4.06288309699722E-03
 1.15082155872387E-03
 6.70172560278909E-03
 2.04064595720209E-04
 1.07869635303671E-03
 7.00980735774871E-05
 2.12028369395825E-04
 1.97297352383669E-03
 1.34150605461642E-03
 1.0135245082314E-03
 1.14949285159133E-03
 8.2051282051282E-03
 3.74045440095236E-03
 2.13700473778497E-03

 0
 0
 1.16908577492401E-03
 2.07967424262284E-03
 3.90788293260311E-03
 1.15082155872387E-03
 6.30621392787039E-03
 2.04064595720209E-04
 9.99915158713805E-04
 7.00980735774871E-05
 1.96126241691138E-04
 1.83029981860057E-03
 1.22733532656396E-03
 8.18258318572138E-04
 1.11683680467112E-03
 8.15705128205128E-03
 3.62546755680012E-03
 1.93694471977957E-03

 0
 0
 6.29507724959082E-04
 1.73888728689893E-03
 4.13333771717637E-04
 7.4590286213584E-04
 5.12700319339056E-03
 9.73944661391906E-05
 1.99983031742761E-04
 3.82353128604475E-05
 3.71049646442694E-05
 1.27591056396877E-03
 6.27939004288538E-04
 1.25528264780953E-04
 0
 6.90705128205128E-03
 2.24562542697321E-03
 1.25946874971582E-03

 0
 0
 3.05760894980126E-04
 4.36906353492193E-05
 2.74773018698656E-03
 1.06557551733691E-04
 3.51565933261067E-04
 5.10161489300522E-05
 5.15107809034385E-04
 3.18627607170396E-05
 7.42099292885388E-05
 3.913335915048E-04
 5.77989310765586E-04
 4.27725939253618E-04
 1.06458712959879E-03
 5.92948717948718E-04
 6.62865336877634E-04
 5.95633235425173E-04

 0
 0
 2.33817154984802E-04
 2.35929430885784E-04
 1.64394113751333E-04
 2.55738124160859E-04
 4.6143028740515E-04
 2.31891586045692E-05
 2.48463766710703E-04
 0
 0
 2.8534741047225E-05
 0
 1.85967799675486E-05
 3.91872563042499E-05
 5.76923076923077E-04
 5.07294900671658E-04
 9.09363718206371E-06

 0
 0
 0
 0
 2.39545708609085E-04

 0
 0
 0
 6.11668894889069E-05
 2.11363860537428E-04
 0
 2.34377288840711E-04
 0
 0
 0
 0
 0
 0
 2.78951699513229E-05
 0
 8.01282051282051E-05
 1.28514708170153E-04
 4.09213673192867E-05

 0
 0
 0
 0
 0
 4.26230206934765E-05
 0
 0
 3.63605512259566E-05
 0
 0
 1.2229174734525E-04
 0
 2.18512164618696E-04

 0
 0
 0
 0
 1.03333442929409E-04
 0
 6.59186124864501E-05
 0
 0
 0
 4.77063831140607E-05
 1.2229174734525E-05
 0
 0
 1.30624187680833E-05
 0
 0
 3.1827730137223E-05

 0
 0
 0
 0
 0
 0
 0
 3.24648220463969E-05
 0
 0
 3.71049646442694E-05
 0
 0
 0
 0
 0
 8.11671841074653E-05

 0
 0
 0
 0
 0
 0
 6.59186124864501E-05

 0
 0
 0
 0
 2.8181848071657E-05

 0
 0
 0
 0
 0
 0
 0
 0
 0
 0
 0
 0
 2.14070115098365E-05

 0
 0
 0
 3.49525082793754E-05
 1.17424366965238E-04
 0
 3.955116749187E-04
 0
 0
 0
 0
 1.42673705236125E-04
 1.14170728052461E-04
 7.43871198701945E-05
 3.26560469202082E-05
 4.80769230769231E-05
 0
 2.00060018005402E-04

 0
 0
 0
 3.49525082793754E-05
 1.17424366965238E-04
 0
 3.955116749187E-04
 0
 0
 0
 0
 1.42673705236125E-04
 1.14170728052461E-04
 7.43871198701945E-05
 3.26560469202082E-05
 4.80769230769231E-05
 0
 2.00060018005402E-04

 0
 0
 0
 0
 2.34848733930475E-05
 0
 0
 0
 0
 0
 1.59021277046869E-05
 0
 0
 1.20879069789066E-04
 0
 0
 8.79311161164208E-05

 0
 0
 0
 0
 2.34848733930475E-05
 0
 0
 0
 0
 0
 1.59021277046869E-05
 0
 0
 1.20879069789066E-04
 0
 0
 8.79311161164208E-05

 0
 0
 1.61873414989478E-04
 0
 0
 0
 0
 0
 6.66610105809204E-05

 0
 0
 8.99296749941546E-05
 0
 0
 0
 0
 0
 6.66610105809204E-05

 0
 0
 7.19437399953237E-05

 0
 0
 0
 0
 1.40909240358285E-05
 0
 0
 0
 1.21201837419855E-05
 0
 0
 0
 0
 0
 0
 0
 2.70557280358218E-05

 0
 0
 0
 0
 1.40909240358285E-05
 0
 0
 0
 1.21201837419855E-05
 0
 0
 0
 0
 0
 0
 0
 2.70557280358218E-05

 0
 0
 0
 0
 1.83182012465771E-04
 0
 3.31057920487505E-03
 0
 4.24206430969493E-05
 0
 0
 0
 0
 0
 0
 8.49358974358974E-04
 1.62334368214931E-04

 0
 0
 0
 0
 1.83182012465771E-04
 0
 3.31057920487505E-03
 0
 4.24206430969493E-05
 0
 0
 0
 0
 0
 0
 8.49358974358974E-04
 1.62334368214931E-04

 0
 0
 0
 0
 1.83182012465771E-04
 0
 3.31057920487505E-03
 0
 4.24206430969493E-05
 0
 0
 0
 0
 0
 0
 8.49358974358974E-04
 1.62334368214931E-04

 0
 0
 3.59718699976618E-05
 0
 0
 0
 0
 0
 3.5148532851758E-04

 0
 0
 0
 0
 0
 0
 0
 0
 2.78764226065667E-04

 0
 0
 0
 0
 0
 0
 0
 0
 1.7574266425879E-04

 0
 0
 0
 0
 0
 0
 0
 0
 1.03021561806877E-04

 0
 0
 0
 0
 0
 0
 0
 0
 7.27211024519132E-05

 0
 0
 0
 0
 0
 0
 0
 0
 7.27211024519132E-05

 0
 0
 3.59718699976618E-05

 0
 0
 3.59718699976618E-05

 0
 0
 3.05760894980125E-04
 1.1359565190797E-04
 6.34091581612283E-04
 8.52460413869531E-05
 1.12794070254592E-03
 1.85513268836554E-05
 6.3630964645424E-04
 0
 1.20326099632131E-03
 0
 0
 0
 1.04499350144666E-04
 6.73076923076923E-04
 5.27586696698525E-04
 3.1827730137223E-05

 0
 0
 1.9784528498714E-04
 7.86431436285947E-05
 3.00606379431008E-04
 8.52460413869531E-05
 6.81158995693317E-04
 0
 1.69682572387797E-04
 0
 1.19265957785152E-03
 0
 0
 0
 0
 8.01282051282051E-05
 1.62334368214931E-04

 0
 0
 1.9784528498714E-04
 7.86431436285947E-05
 3.00606379431008E-04
 8.52460413869531E-05
 6.81158995693317E-04
 0
 1.69682572387797E-04
 0
 1.19265957785152E-03
 0
 0
 0
 0
 8.01282051282051E-05
 1.62334368214931E-04

 0
 0
 1.9784528498714E-04
 7.86431436285947E-05
 2.8181848071657E-04
 8.52460413869531E-05
 6.81158995693317E-04
 0
 1.69682572387797E-04
 0
 1.19265957785152E-03
 0
 0
 0
 0
 8.01282051282051E-05
 1.62334368214931E-04

 0
 0
 0
 0
 1.8787898714438E-05

 0
 0
 1.07915609992985E-04
 3.49525082793754E-05
 3.33485202181275E-04
 0
 4.46781706852606E-04
 1.85513268836554E-05
 4.66627074066443E-04
 0
 1.06014184697913E-05
 0
 0
 0
 1.04499350144666E-04
 5.92948717948718E-04
 3.65252328483594E-04
 3.1827730137223E-05

 0
 0
 1.07915609992985E-04
 3.49525082793754E-05
 3.33485202181275E-04
 0
 4.46781706852606E-04
 1.85513268836554E-05
 4.66627074066443E-04
 0
 1.06014184697913E-05
 0
 0
 0
 1.04499350144666E-04
 5.92948717948718E-04
 3.65252328483594E-04
 3.1827730137223E-05

 0
 0
 1.07915609992985E-04
 3.49525082793754E-05
 2.30151759251866E-04
 0
 3.58890223537339E-04
 1.85513268836554E-05
 1.0908165367787E-04
 0
 0
 0
 0
 0
 1.04499350144666E-04
 5.44871794871795E-04
 2.77321212367173E-04
 3.1827730137223E-05

 0
 0
 0
 0
 1.03333442929409E-04
 0
 8.78914833152667E-05
 0
 3.57545420388573E-04
 0
 0
 0
 0
 0
 0
 4.80769230769231E-05
 8.79311161164208E-05

 0
 0
 0
 0
 0
 0
 0
 0
 0
 0
 1.06014184697913E-05

 0
 0
 1.43887479990647E-04
 1.1359565190797E-04
 9.86364682507997E-05
 1.2786906208043E-04
 5.93267512378051E-04
 0
 3.81785787872544E-04
 0
 0
 0
 0
 0
 0
 2.40384615384615E-04
 3.51724464465683E-04
 2.27340929551593E-05

 0
 0
 1.43887479990647E-04
 1.1359565190797E-04
 9.86364682507997E-05
 1.2786906208043E-04
 5.93267512378051E-04
 0
 3.81785787872544E-04
 0
 0
 0
 0
 0
 0
 2.40384615384615E-04
 3.51724464465683E-04
 2.27340929551593E-05

 0
 0
 1.43887479990647E-04
 1.1359565190797E-04
 9.86364682507997E-05
 1.2786906208043E-04
 5.93267512378051E-04
 0
 3.81785787872544E-04
 0
 0
 0
 0
 0
 0
 2.40384615384615E-04
 3.51724464465683E-04
 2.27340929551593E-05

 0
 0
 1.43887479990647E-04
 1.1359565190797E-04
 9.86364682507997E-05
 1.2786906208043E-04
 5.93267512378051E-04
 0
 3.81785787872544E-04
 0
 0
 0
 0
 0
 0
 2.40384615384615E-04
 3.51724464465683E-04
 2.27340929551593E-05

 0
 0
 3.59718699976618E-05
 0
 0
 0
 0
 0
 0
 0
 0
 0
 0
 0
 0
 0
 1.08222912143287E-04

 0
 0
 3.59718699976618E-05
 0
 0
 0
 0
 0
 0
 0
 0
 0
 0
 0
 0
 0
 1.08222912143287E-04

 0
 0
 3.59718699976618E-05
 0
 0
 0
 0
 0
 0
 0
 0
 0
 0
 0
 0
 0
 1.08222912143287E-04

 0
 0
 3.59718699976618E-05
 0
 0
 0
 0
 0
 0
 0
 0
 0
 0
 0
 0
 0
 1.08222912143287E-04

 0
 0
 0
 4.36906353492193E-05
 1.08030417608019E-04
 1.06557551733691E-04
 1.01075205812557E-03
 3.26364218200707E-02
 3.0179257517544E-03
 4.57166890768084E-02
 8.93805591188101E-02
 3.587224588794E-04
 9.27637165426249E-05
 8.45223649525084E-03

 0
 0
 0
 4.36906353492193E-05
 1.08030417608019E-04
 1.06557551733691E-04
 1.01075205812557E-03
 3.26364218200707E-02
 3.0179257517544E-03
 4.57166890768084E-02
 8.93805591188101E-02
 3.587224588794E-04
 9.27637165426249E-05
 8.45223649525084E-03

 0
 0
 0
 4.36906353492193E-05
 1.08030417608019E-04
 1.06557551733691E-04
 1.01075205812557E-03
 3.26364218200707E-02
 3.0179257517544E-03
 4.57166890768084E-02
 8.93805591188101E-02
 3.587224588794E-04
 9.27637165426249E-05
 8.45223649525084E-03

 0
 0
 0
 4.36906353492193E-05
 1.08030417608019E-04
 1.06557551733691E-04
 1.01075205812557E-03
 3.26364218200707E-02
 2.96338492491546E-03
 4.57166890768084E-02
 8.93805591188101E-02
 3.587224588794E-04
 9.27637165426249E-05
 8.45223649525084E-03

 0
 0
 0
 4.36906353492193E-05
 1.08030417608019E-04
 1.06557551733691E-04
 1.01075205812557E-03
 3.13378289382148E-02
 2.21799362478335E-03
 4.36073743173404E-02
 .080851717959863
 3.587224588794E-04
 9.27637165426249E-05
 8.34530501043744E-03

 0
 0
 0
 0
 0
 0
 0
 1.04351213720561E-03
 3.87845879743537E-04
 1.86715777801852E-03
 6.96513193465286E-03
 0
 0
 6.50887298864202E-05

 0
 0
 0
 0
 0
 0
 0
 2.55080744650261E-04
 0
 2.42156981449501E-04
 1.56370922429421E-03

 0
 0
 0
 0
 0
 0
 0
 0
 3.57545420388573E-04

 0
 0
 0
 0
 0
 0
 0
 0
 0
 0
 0
 0
 0
 4.18427549269844E-05

 0
 0
 0
 0
 0
 0
 0
 0
 5.45408268389349E-05

 0
 0
 0
 0
 0
 0
 0
 0
 5.45408268389349E-05

 0
 0
 .017122610118887
 .014662577223198
 6.48135535901326E-02
 2.21213477399143E-02
 6.05279348431136E-02
 6.86399094695248E-04
 1.26352915510199E-02
 1.18529469867387E-03
 .02310579155491
 3.03568880826692E-02
 3.08689105971843E-02
 7.37827245212491E-03
 4.09114955816369E-02
 6.18108974358974E-02
 9.73397455408778E-02
 9.28005674429602E-02

 0
 0
 6.47493659957913E-04
 1.74762541396877E-05
 7.98485695363616E-05
 2.1737740553673E-03
 2.27052998564439E-04
 6.95674758137076E-05
 1.81802756129783E-04
 7.6470625720895E-05
 2.12028369395825E-05
 1.01909789454375E-04
 2.14070115098365E-05
 6.35080035891785E-03
 9.27431732533913E-04
 6.41025641025641E-05
 .035368600474828
 2.86449571235007E-04

 0
 0
 6.47493659957913E-04
 1.74762541396877E-05
 7.98485695363616E-05
 2.1737740553673E-03
 2.27052998564439E-04
 6.95674758137076E-05
 1.81802756129783E-04
 7.6470625720895E-05
 2.12028369395825E-05
 1.01909789454375E-04
 2.14070115098365E-05
 6.35080035891785E-03
 9.27431732533913E-04
 6.41025641025641E-05
 .035368600474828
 2.86449571235007E-04

 0
 0
 6.47493659957913E-04
 1.74762541396877E-05
 7.98485695363616E-05
 2.1737740553673E-03
 2.27052998564439E-04
 6.95674758137076E-05
 1.81802756129783E-04
 7.6470625720895E-05
 2.12028369395825E-05
 1.01909789454375E-04
 2.14070115098365E-05
 6.35080035891785E-03
 9.27431732533913E-04
 6.41025641025641E-05
 .035368600474828
 2.86449571235007E-04

 0
 0
 6.47493659957913E-04
 1.74762541396877E-05
 7.98485695363616E-05
 2.1737740553673E-03
 2.27052998564439E-04
 6.95674758137076E-05
 1.81802756129783E-04
 7.6470625720895E-05
 2.12028369395825E-05
 1.01909789454375E-04
 2.14070115098365E-05
 6.35080035891785E-03
 9.27431732533913E-04
 6.41025641025641E-05
 .035368600474828
 2.86449571235007E-04

 0
 0
 6.4029928595838E-03
 5.83706888265569E-03
 4.92618704292565E-02
 6.75574877991603E-03
 2.70998740222072E-02
 2.78269903254831E-05
 6.70852170118899E-03
 5.09804171472634E-05
 1.50858184825129E-02
 2.0381957890875E-04
 9.70451188445922E-04
 2.32459749594358E-05
 8.03338754237122E-03
 4.42788461538461E-02
 3.06067923405234E-02
 1.16398555930415E-02

 0
 0
 4.64037122969837E-03
 2.10588862383237E-03
 .027063968098148
 5.02951644183023E-03
 1.21290246975068E-02
 9.27566344182768E-06
 5.50256341886143E-03
 2.54902085736317E-05
 1.40362780540036E-02
 2.8534741047225E-05
 2.21205785601644E-04
 0
 5.29027960107373E-04
 3.90544871794871E-02
 2.20030708251321E-02
 1.7550719761383E-03

 0
 0
 3.39934171477904E-03
 1.62529163499096E-03
 2.29447213050074E-02
 3.83607186241289E-03
 9.60946884246915E-03
 9.27566344182768E-06
 3.41789181523992E-03
 0
 1.16615603167704E-04
 2.0381957890875E-05
 1.35577739562298E-04
 0
 4.3759102873079E-04
 3.73237179487179E-02
 1.91892751094066E-02
 1.41860740040194E-03

 0
 0
 2.82379179481645E-03
 4.01953845212817E-04
 1.27992559992109E-02
 2.55738124160859E-03
 3.26663346321741E-03
 0
 2.29677481910626E-03
 0
 1.16615603167704E-04
 2.0381957890875E-05
 1.2130639855574E-04
 0
 3.52685306738249E-04
 3.21634615384615E-02
 1.38931163463945E-02
 9.23004173979467E-04

 0
 0
 8.99296749941546E-05
 6.99050165587508E-04
 6.66031009426828E-03
 3.40984165547812E-04
 4.4824656490786E-03
 9.27566344182768E-06
 1.21201837419855E-04
 0
 0
 0
 0
 0
 0
 3.52564102564103E-03
 1.49482897397915E-03

 0
 0
 4.85620244968435E-04
 5.24287624190631E-04
 1.54530466926253E-03
 9.37706455256484E-04
 1.68458676354261E-03
 0
 9.99915158713806E-04
 0
 0
 0
 1.42713410065577E-05
 0
 0
 1.63461538461538E-03
 3.72692653693445E-03

 0
 0
 0
 0
 1.93985054226573E-03
 0
 1.75782966630533E-04
 0
 0
 0
 0
 0
 0
 0
 8.49057219925414E-05
 0
 7.44032520985099E-05
 4.95603226422472E-04

 0
 0
 8.27353009946222E-04
 5.24287624190631E-05
 2.48939657966304E-03
 7.88525882829317E-04
 3.07620191603433E-04
 0
 1.12717708800465E-03
 2.54902085736317E-05
 1.38878581954265E-02
 0
 6.42210345295096E-05
 0
 5.87808844563748E-05
 1.6025641025641E-04
 1.19045203357616E-03
 2.13700473778497E-04

 0
 0
 1.07915609992985E-04
 0
 1.37621358083259E-03
 1.2786906208043E-04
 1.75782966630533E-04
 0
 5.02987625292399E-04
 2.54902085736317E-05
 1.38878581954265E-02
 0
 6.42210345295096E-05
 0
 5.87808844563748E-05
 1.6025641025641E-04
 1.69098300223886E-04
 2.13700473778497E-04

 0
 0
 7.19437399953237E-04
 5.24287624190631E-05
 1.11318299883045E-03
 6.60656820748887E-04
 1.318372249729E-04
 0
 6.24189462712255E-04
 0
 0
 0
 0
 0
 0
 0
 1.02135373335227E-03

 0
 0
 4.13676504973111E-04
 4.28168226422349E-04
 1.6298502134775E-03
 4.04918696588027E-04
 2.21193566343421E-03
 0
 9.57494515616857E-04
 0
 3.18042554093738E-05
 8.15278315635E-06
 2.14070115098365E-05
 0
 3.26560469202082E-05
 1.57051282051282E-03
 1.62334368214931E-03
 1.2276410195786E-04

 0
 0
 4.13676504973111E-04
 4.28168226422349E-04
 1.6298502134775E-03
 4.04918696588027E-04
 2.21193566343421E-03
 0
 9.57494515616857E-04
 0
 3.18042554093738E-05
 8.15278315635E-06
 2.14070115098365E-05
 0
 3.26560469202082E-05
 1.57051282051282E-03
 1.62334368214931E-03
 1.2276410195786E-04

 0
 0
 1.72664975988777E-03
 3.73118025882332E-03
 2.20006293946069E-02
 1.7262323380858E-03
 1.49708493247004E-02
 0
 1.20595828232756E-03
 0
 5.56574469664041E-04
 1.141389641889E-04
 5.06632605732798E-04
 0
 7.27576725382239E-03
 5.22435897435898E-03
 8.60372151539133E-03
 8.94813898715069E-03

 0
 0
 1.72664975988777E-03
 3.73118025882332E-03
 2.20006293946069E-02
 1.7262323380858E-03
 1.49708493247004E-02
 0
 1.20595828232756E-03
 0
 5.56574469664041E-04
 1.141389641889E-04
 5.06632605732798E-04
 0
 7.27576725382239E-03
 5.22435897435898E-03
 8.60372151539133E-03
 8.94813898715069E-03

 0
 0
 3.77704634975449E-04
 5.76716386609694E-04
 6.34091581612284E-03
 2.34426613814121E-04
 1.23048076641373E-03
 0
 9.09013780648915E-05
 0
 0
 0
 4.2814023019673E-05
 0
 6.53120938404164E-05
 3.0448717948718E-04
 7.03448928931366E-04
 1.90966380823338E-04

 0
 0
 0
 8.12645817495478E-04
 1.8787898714438E-03
 1.06557551733691E-04
 4.06498110333109E-03
 0
 1.33322021161841E-04
 0
 0
 0
 1.42713410065577E-04
 0
 5.83890118933323E-03
 1.44230769230769E-03
 8.79311161164208E-04
 4.46497585639328E-03

 0
 0
 4.67634309969604E-04
 1.20586153563845E-03
 5.69743028515333E-03
 5.32787758668457E-04
 3.75736091172765E-03
 0
 4.06026155356515E-04
 0
 1.11314893932808E-04
 2.0381957890875E-05
 1.99798774091808E-04
 0
 4.57184656882915E-05
 1.65064102564103E-03
 2.49589091130456E-03
 1.79144652486655E-03

 0
 0
 6.29507724959082E-04
 7.34002673866883E-04
 1.89757777015824E-03
 6.81968331095625E-04
 3.69876658951748E-03
 0
 4.78747257808428E-04
 0
 0
 0
 0
 0
 0
 1.74679487179487E-03
 3.50371678063892E-03

 0
 0
 1.9784528498714E-04
 2.97096320374691E-04
 .002766518085701
 1.70492082773906E-04
 1.41358802332054E-03
 0
 0
 0
 0
 0
 0
 0
 1.18214889851154E-03
 0
 2.84085144376129E-04
 2.20975383524148E-03

 0
 0
 0
 1.04857524838126E-04
 1.70969878301386E-03
 0
 4.46781706852606E-04
 0
 5.45408268389349E-05
 0
 2.27930497100512E-04
 6.9298656828975E-05
 9.27637165426249E-05
 0
 3.91872563042499E-05
 0
 3.17904804420906E-04
 1.09123646184765E-04

 0
 0
 0
 0
 1.57348651733419E-03
 0
 9.5215773591539E-05
 0
 0
 0
 2.17329078630721E-04
 2.445834946905E-05
 2.85426820131154E-05
 0
 1.04499350144666E-04
 8.01282051282051E-05
 2.2320975629553E-04
 1.81872743641274E-04

 0
 0
 5.39578049964927E-05
 0
 1.36212265679676E-04
 0
 2.636744499458E-04
 0
 4.24206430969493E-05
 0
 0
 0
 0
 0
 0
 0
 1.96154028259708E-04

 0
 0
 0
 0
 0
 0
 0
 0
 0
 0
 0
 2.0381957890875E-05
 1.56984751072134E-04
 0
 1.82873862753166E-04
 0
 0
 6.50195058517555E-04

 0
 0
 0
 0
 0
 0
 0
 0
 0
 0
 0
 2.0381957890875E-05
 1.56984751072134E-04
 0
 1.82873862753166E-04
 0
 0
 6.50195058517555E-04

 0
 0
 0
 0
 0
 0
 0
 0
 0
 0
 0
 2.0381957890875E-05
 1.56984751072134E-04
 0
 1.82873862753166E-04
 0
 0
 6.50195058517555E-04

 0
 0
 3.59718699976618E-05
 0
 7.04546201791426E-05
 0
 0
 1.85513268836554E-05
 0
 2.54902085736317E-05
 4.29357448026546E-04
 4.076391578175E-05
 8.56280460393461E-05
 2.32459749594358E-05
 4.57184656882915E-05
 0
 0
 2.86449571235007E-04

 0
 0
 3.59718699976618E-05
 0
 7.04546201791426E-05
 0
 0
 1.85513268836554E-05
 0
 2.54902085736317E-05
 4.29357448026546E-04
 4.076391578175E-05
 8.56280460393461E-05
 2.32459749594358E-05
 4.57184656882915E-05
 0
 0
 2.86449571235007E-04

 0
 0
 3.59718699976618E-05
 0
 7.04546201791426E-05
 0
 0
 1.85513268836554E-05
 0
 2.54902085736317E-05
 4.29357448026546E-04
 4.076391578175E-05
 8.56280460393461E-05
 2.32459749594358E-05
 4.57184656882915E-05
 0
 0
 2.86449571235007E-04

 0
 0
 0
 0
 1.26818316322457E-04
 0
 0
 0
 0
 0
 6.36085108187475E-05

 0
 0
 0
 0
 1.26818316322457E-04
 0
 0
 0
 0
 0
 6.36085108187475E-05

 0
 0
 0
 0
 1.26818316322457E-04
 0
 0
 0
 0
 0
 2.65035461744781E-05

 0
 0
 0
 0
 0
 0
 0
 0
 0
 0
 3.71049646442694E-05

 0
 0
 1.49283260490297E-03
 6.40504714219554E-03
 6.90924975223459E-03
 1.70492082773906E-03
 1.71901092784109E-02
 7.88431392555352E-05
 1.01203534245579E-03
 2.10294220732461E-04
 6.36085108187475E-04
 1.91386584595316E-02
 1.34721459101905E-02
 5.067622541157E-04
 9.25472369718701E-03
 5.91346153846154E-03
 7.03448928931366E-03
 2.84176161939491E-02

 0
 0
 1.49283260490297E-03
 6.40504714219554E-03
 6.90924975223459E-03
 1.70492082773906E-03
 1.71901092784109E-02
 7.88431392555352E-05
 1.01203534245579E-03
 2.10294220732461E-04
 6.36085108187475E-04
 1.91386584595316E-02
 1.34721459101905E-02
 5.067622541157E-04
 9.25472369718701E-03
 5.91346153846154E-03
 7.03448928931366E-03
 2.84176161939491E-02

 0
 0
 1.49283260490297E-03
 6.40504714219554E-03
 6.90924975223459E-03
 1.70492082773906E-03
 1.71901092784109E-02
 7.88431392555352E-05
 1.01203534245579E-03
 2.10294220732461E-04
 6.36085108187475E-04
 1.91386584595316E-02
 1.34721459101905E-02
 5.067622541157E-04
 9.25472369718701E-03
 5.91346153846154E-03
 7.03448928931366E-03
 2.84176161939491E-02

 0
 0
 1.49283260490297E-03
 6.40504714219554E-03
 6.90924975223459E-03
 1.70492082773906E-03
 1.71901092784109E-02
 7.88431392555352E-05
 1.01203534245579E-03
 2.10294220732461E-04
 6.36085108187475E-04
 1.91386584595316E-02
 1.34721459101905E-02
 5.067622541157E-04
 9.25472369718701E-03
 5.91346153846154E-03
 7.03448928931366E-03
 2.84176161939491E-02

 0
 0
 6.6907678195651E-03
 9.96146485962199E-04
 4.22258023606995E-03
 8.14099695245402E-03
 9.38241584390472E-03
 5.10161489300522E-04
 4.0178409104682E-03
 8.47549435073253E-04
 7.32558016262576E-03
 1.08798891221491E-02
 1.63478211230118E-02
 4.97463864131925E-04
 2.21342686025172E-02
 5.83333333333334E-03
 1.66460366740393E-02
 .052238398792365

 0
 0
 6.09723196460368E-03
 8.47598325774854E-04
 3.88439805921007E-03
 7.41640560066492E-03
 8.05671930389945E-03
 5.10161489300522E-04
 3.62999503072467E-03
 8.47549435073253E-04
 7.2778737795117E-03
 1.08798891221491E-02
 1.63478211230118E-02
 4.97463864131925E-04
 2.21342686025172E-02
 5.20833333333334E-03
 1.50159290598811E-02
 .052238398792365

 0
 0
 6.09723196460368E-03
 8.47598325774854E-04
 3.88439805921007E-03
 7.41640560066492E-03
 8.05671930389945E-03
 5.10161489300522E-04
 3.62999503072467E-03
 8.47549435073253E-04
 7.2778737795117E-03
 1.08798891221491E-02
 1.63478211230118E-02
 4.97463864131925E-04
 2.21342686025172E-02
 5.20833333333334E-03
 1.50159290598811E-02
 .052238398792365

 0
 0
 1.06117016493102E-03
 1.22333778977814E-04
 9.39394935721902E-06
 1.12951004837713E-03
 5.49321770720417E-04
 0
 5.93889003357291E-04
 0
 0
 0
 6.27939004288538E-04
 0
 1.56944961498521E-02
 2.40384615384615E-04
 1.1228127134866E-03
 2.10972382623878E-02

 0
 0
 2.32018561484919E-03
 5.33025751260475E-04
 3.26909437631222E-03
 3.11148051062379E-03
 6.28424105704157E-03
 0
 1.31503993600543E-03
 0
 0
 9.7507286549946E-03
 8.25597077229362E-03
 2.78951699513229E-05
 4.49347205622065E-03
 4.15064102564103E-03
 7.5756038500301E-03
 1.77871543281166E-02

 0
 0
 2.55400276983399E-03
 1.31071906047658E-04
 4.93182341253998E-04
 .003175415041664
 9.30184865086573E-04
 4.82334498975039E-04
 1.31503993600543E-03
 8.22059226499621E-04
 3.17512483170248E-03
 1.06393820190367E-03
 7.42109732340999E-03
 4.60270304196828E-04
 1.92670676829228E-03
 6.89102564102564E-04
 5.82374545971064E-03
 1.33403657460875E-02

 0
 0
 0
 0
 0
 0
 0
 2.7826990325483E-05
 4.84807349679421E-05
 2.54902085736317E-05
 2.77757163908531E-03
 0
 0
 9.29838998377431E-06

 0
 0
 0
 0
 0
 0
 0
 0
 3.21184869162617E-04
 0
 1.32517730872391E-03

 0
 0
 1.61873414989478E-04
 6.11668894889069E-05
 1.12727392286628E-04
 0
 2.92971611050889E-04
 0
 3.63605512259566E-05
 0
 0
 6.52222652508E-05
 4.2814023019673E-05
 0
 1.95936281521249E-05
 1.28205128205128E-04
 4.93767036653748E-04
 1.36404557730956E-05

 0
 0
 5.9353585496142E-04
 1.48548160187345E-04
 3.38182176859885E-04
 7.24591351789101E-04
 1.32569654000527E-03
 0
 3.87845879743537E-04
 0
 0
 0
 0
 0
 0
 .000625
 1.63010761415826E-03

 0
 0
 5.9353585496142E-04
 1.48548160187345E-04
 3.38182176859885E-04
 7.24591351789101E-04
 1.32569654000527E-03
 0
 3.87845879743537E-04
 0
 0
 0
 0
 0
 0
 .000625
 1.63010761415826E-03

 0
 0
 5.9353585496142E-04
 1.48548160187345E-04
 3.38182176859885E-04
 7.24591351789101E-04
 1.32569654000527E-03
 0
 3.87845879743537E-04
 0
 0
 0
 0
 0
 0
 .000625
 1.63010761415826E-03

 0
 0
 0
 0
 0
 0
 0
 0
 0
 0
 4.77063831140607E-05

 0
 0
 0
 0
 0
 0
 0
 0
 0
 0
 4.77063831140607E-05

 0
 0
 0
 0
 0
 0
 0
 0
 0
 0
 4.77063831140607E-05

 0
 0
 4.67634309969604E-04
 1.18838528149876E-03
 2.34848733930475E-04
 7.24591351789101E-04
 4.96586880731257E-03
 0
 1.15141745548863E-04
 0
 0
 0
 0
 0
 1.30624187680833E-05
 3.99038461538462E-03
 1.62334368214931E-03

 0
 0
 4.67634309969604E-04
 1.18838528149876E-03
 2.34848733930475E-04
 7.24591351789101E-04
 4.96586880731257E-03
 0
 1.15141745548863E-04
 0
 0
 0
 0
 0
 1.30624187680833E-05
 3.99038461538462E-03
 1.62334368214931E-03

 0
 0
 4.67634309969604E-04
 1.18838528149876E-03
 2.34848733930475E-04
 7.24591351789101E-04
 4.96586880731257E-03
 0
 1.15141745548863E-04
 0
 0
 0
 0
 0
 1.30624187680833E-05
 3.99038461538462E-03
 1.62334368214931E-03

 0
 0
 4.67634309969604E-04
 1.18838528149876E-03
 2.34848733930475E-04
 7.24591351789101E-04
 4.96586880731257E-03
 0
 1.15141745548863E-04
 0
 0
 0
 0
 0
 1.30624187680833E-05
 3.99038461538462E-03
 1.62334368214931E-03

 0
 0
 1.07915609992985E-03
 1.22333778977814E-04
 5.30758138682874E-04
 1.87541291051297E-03
 5.41997480444145E-04
 0
 1.69682572387797E-04
 0
 3.71049646442694E-05
 0
 0
 0
 5.48621588259498E-04
 1.33012820512821E-03
 4.23422143760611E-03
 1.18217283366828E-04

 0
 0
 1.07915609992985E-03
 1.22333778977814E-04
 5.30758138682874E-04
 1.87541291051297E-03
 5.41997480444145E-04
 0
 1.69682572387797E-04
 0
 3.71049646442694E-05
 0
 0
 0
 5.48621588259498E-04
 1.33012820512821E-03
 4.23422143760611E-03
 1.18217283366828E-04

 0
 0
 1.07915609992985E-03
 1.22333778977814E-04
 5.30758138682874E-04
 1.87541291051297E-03
 5.41997480444145E-04
 0
 1.69682572387797E-04
 0
 3.71049646442694E-05
 0
 0
 0
 5.48621588259498E-04
 1.33012820512821E-03
 4.23422143760611E-03
 1.18217283366828E-04

 0
 0
 1.07915609992985E-03
 1.22333778977814E-04
 5.30758138682874E-04
 1.87541291051297E-03
 5.41997480444145E-04
 0
 1.69682572387797E-04
 0
 3.71049646442694E-05
 0
 0
 0
 5.48621588259498E-04
 1.33012820512821E-03
 4.23422143760611E-03
 1.18217283366828E-04

 0
 0
 0
 0
 3.52273100895713E-03
 0
 6.73834705417045E-04
 0
 1.81802756129783E-05
 0
 0
 0
 0
 0
 0
 4.00641025641026E-04

 0
 0
 0
 0
 3.52273100895713E-03
 0
 6.73834705417045E-04
 0
 1.81802756129783E-05
 0
 0
 0
 0
 0
 0
 4.00641025641026E-04

 0
 0
 0
 0
 3.52273100895713E-03
 0
 6.73834705417045E-04
 0
 1.81802756129783E-05
 0
 0
 0
 0
 0
 0
 4.00641025641026E-04

 0
 0
 0
 0
 3.52273100895713E-03
 0
 6.73834705417045E-04
 0
 1.81802756129783E-05
 0
 0
 0
 0
 0
 0
 4.00641025641026E-04

 0
 0
 2.51803089983633E-04
 0
 0
 6.60656820748887E-04
 2.19728708288167E-05
 0
 2.24223399226732E-04
 0
 0
 0
 0
 0
 0
 0
 1.11604878147765E-03
 5.91086416834141E-05

 0
 0
 2.51803089983633E-04
 0
 0
 6.60656820748887E-04
 2.19728708288167E-05
 0
 2.24223399226732E-04
 0
 0
 0
 0
 0
 0
 0
 1.11604878147765E-03
 5.91086416834141E-05

 0
 0
 2.51803089983633E-04
 0
 0
 6.60656820748887E-04
 2.19728708288167E-05
 0
 2.24223399226732E-04
 0
 0
 0
 0
 0
 0
 0
 1.11604878147765E-03
 5.91086416834141E-05

 0
 0
 2.51803089983633E-04
 0
 0
 6.60656820748887E-04
 2.19728708288167E-05
 0
 2.24223399226732E-04
 0
 0
 0
 0
 0
 0
 0
 1.11604878147765E-03
 5.91086416834141E-05

 0
 0
 8.99296749941546E-05
 9.61193977682823E-05
 5.16667214647046E-05
 8.52460413869531E-05
 4.24808836023789E-04
 0
 1.87862848000776E-04
 0
 0
 3.26111326254E-05
 5.70853640262307E-05
 0
 0
 0
 7.10212860940322E-04
 4.09213673192867E-05

 0
 0
 8.99296749941546E-05
 9.61193977682823E-05
 5.16667214647046E-05
 8.52460413869531E-05
 4.24808836023789E-04
 0
 1.87862848000776E-04
 0
 0
 3.26111326254E-05
 5.70853640262307E-05
 0
 0
 0
 7.10212860940322E-04
 4.09213673192867E-05

 0
 0
 8.99296749941546E-05
 9.61193977682823E-05
 5.16667214647046E-05
 8.52460413869531E-05
 4.24808836023789E-04
 0
 1.87862848000776E-04
 0
 0
 3.26111326254E-05
 5.70853640262307E-05
 0
 0
 0
 7.10212860940322E-04
 4.09213673192867E-05

 0
 0
 8.99296749941546E-05
 9.61193977682823E-05
 5.16667214647046E-05
 8.52460413869531E-05
 4.24808836023789E-04
 0
 1.87862848000776E-04
 0
 0
 3.26111326254E-05
 5.70853640262307E-05
 0
 0
 0
 7.10212860940322E-04
 4.09213673192867E-05

 0
 0
 1.05937157143114E-02
 2.96222507667707E-03
 3.10000328788228E-03
 1.47262536495961E-02
 6.98004863328743E-03
 1.02032297860104E-04
 5.1995588253118E-03
 0
 2.01426950926034E-04
 1.2229174734525E-05
 0
 7.90363148620816E-05
 2.74310794129749E-04
 1.46314102564103E-02
 4.14155556908341E-02
 2.00060018005402E-04

 0
 0
 1.03778844943254E-02
 2.96222507667707E-03
 3.10000328788228E-03
 1.43426464633549E-02
 6.96540005273489E-03
 1.02032297860104E-04
 4.36932623898579E-03
 0
 1.6962269551666E-04
 1.2229174734525E-05
 0
 7.90363148620816E-05
 2.74310794129749E-04
 1.44711538461538E-02
 4.04686052095804E-02
 2.00060018005402E-04

 0
 0
 1.83456536988075E-03
 2.1321030050419E-03
 2.28742666848284E-03
 9.73936022845939E-03
 5.97662086543814E-03
 0
 2.1816330735574E-04
 0
 5.30070923489563E-05
 0
 0
 3.71935599350972E-05
 1.1756176891275E-04
 1.29967948717949E-02
 2.70083805117591E-02
 1.68232287868179E-04

 0
 0
 1.83456536988075E-03
 2.1321030050419E-03
 2.28742666848284E-03
 9.73936022845939E-03
 5.97662086543814E-03
 0
 2.1816330735574E-04
 0
 5.30070923489563E-05
 0
 0
 3.71935599350972E-05
 1.1756176891275E-04
 1.29967948717949E-02
 2.70083805117591E-02
 1.68232287868179E-04

 0
 0
 1.83456536988075E-03
 1.38062407703533E-03
 6.20000657576455E-04
 9.73936022845939E-03
 4.53373568101251E-03
 0
 1.03021561806877E-04
 0
 2.12028369395825E-05
 0
 0
 3.71935599350972E-05
 4.57184656882915E-05
 9.26282051282051E-03
 2.52091745973769E-02
 9.5483190411669E-05

 0
 0
 0
 7.51478928006571E-04
 1.66742601090638E-03
 0
 1.44288518442563E-03
 0
 1.15141745548863E-04
 0
 3.18042554093738E-05
 0
 0
 0
 7.18433032244581E-05
 3.73397435897436E-03
 1.79920591438215E-03
 7.27490974565097E-05

 0
 0
 8.54331912444468E-03
 8.30122071635165E-04
 8.12576619399445E-04
 4.60328623489546E-03
 9.8877918729675E-04
 1.02032297860104E-04
 4.15116293163005E-03
 0
 1.16615603167704E-04
 1.2229174734525E-05
 0
 4.18427549269844E-05
 1.56749025216999E-04
 1.47435897435897E-03
 1.34602246978213E-02
 3.1827730137223E-05

 0
 0
 8.54331912444468E-03
 8.30122071635165E-04
 8.12576619399445E-04
 4.60328623489546E-03
 9.8877918729675E-04
 1.02032297860104E-04
 4.15116293163005E-03
 0
 1.16615603167704E-04
 1.2229174734525E-05
 0
 4.18427549269844E-05
 1.56749025216999E-04
 1.47435897435897E-03
 1.34602246978213E-02
 3.1827730137223E-05

 0
 0
 1.07915609992985E-04
 8.73812706984385E-05
 2.34848733930475E-05
 3.40984165547812E-04
 3.2959306243225E-04
 0
 2.36343582968718E-04
 0
 2.12028369395825E-05
 0
 0
 0
 0
 1.29807692307692E-03
 1.16271991233944E-02

 0
 0
 8.4354035144517E-03
 7.42740800936727E-04
 7.56212923256131E-04
 4.26230206934765E-03
 5.20024609615328E-04
 0
 3.91481934866133E-03
 0
 9.54127662281213E-05
 0
 0
 0
 1.56749025216999E-04
 1.76282051282051E-04
 1.83302557442693E-03
 3.1827730137223E-05

 0
 0
 0
 0
 3.28788227502666E-05
 0
 1.39161515249172E-04
 1.02032297860104E-04
 0
 0
 0
 1.2229174734525E-05
 0
 4.18427549269844E-05

 0
 0
 2.15831219985971E-04
 0
 0
 3.83607186241289E-04
 1.46485805525445E-05
 0
 8.30232586326008E-04
 0
 3.18042554093738E-05
 0
 0
 0
 0
 1.6025641025641E-04
 9.46950481253762E-04

 0
 0
 2.15831219985971E-04
 0
 0
 3.83607186241289E-04
 1.46485805525445E-05
 0
 7.87811943229059E-04
 0
 2.12028369395825E-05
 0
 0
 0
 0
 1.6025641025641E-04
 8.86075093173163E-04

 0
 0
 2.15831219985971E-04
 0
 0
 3.83607186241289E-04
 1.46485805525445E-05
 0
 7.87811943229059E-04
 0
 2.12028369395825E-05
 0
 0
 0
 0
 1.6025641025641E-04
 8.86075093173163E-04

 0
 0
 2.15831219985971E-04
 0
 0
 3.83607186241289E-04
 1.46485805525445E-05
 0
 7.87811943229059E-04
 0
 2.12028369395825E-05
 0
 0
 0
 0
 1.6025641025641E-04
 8.86075093173163E-04

 0
 0
 0
 0
 0
 0
 0
 0
 4.24206430969493E-05
 0
 1.06014184697913E-05
 0
 0
 0
 0
 0
 6.0875388080599E-05

 0
 0
 0
 0
 0
 0
 0
 0
 4.24206430969493E-05
 0
 1.06014184697913E-05
 0
 0
 0
 0
 0
 6.0875388080599E-05

 0
 0
 0
 0
 0
 0
 0
 0
 4.24206430969493E-05
 0
 1.06014184697913E-05
 0
 0
 0
 0
 0
 6.0875388080599E-05

 0
 0
 1.61873414989478E-04
 0
 5.49546037397312E-04
 1.02295249664344E-03
 3.22268772155978E-04
 0
 6.42369738325233E-04
 0
 4.77063831140607E-05
 8.15278315635E-06
 2.64733375671645E-03
 1.39475849756615E-05
 5.45355983567477E-03
 2.40384615384615E-04
 1.31896674174631E-02
 3.82842125364882E-03

 0
 0
 1.61873414989478E-04
 0
 5.49546037397312E-04
 1.02295249664344E-03
 3.22268772155978E-04
 0
 6.42369738325233E-04
 0
 4.77063831140607E-05
 8.15278315635E-06
 2.64733375671645E-03
 1.39475849756615E-05
 5.45355983567477E-03
 2.40384615384615E-04
 1.31896674174631E-02
 3.82842125364882E-03

 0
 0
 1.61873414989478E-04
 0
 5.49546037397312E-04
 1.02295249664344E-03
 3.22268772155978E-04
 0
 6.42369738325233E-04
 0
 4.77063831140607E-05
 8.15278315635E-06
 2.64733375671645E-03
 1.39475849756615E-05
 5.45355983567477E-03
 2.40384615384615E-04
 1.31896674174631E-02
 3.82842125364882E-03

 0
 0
 1.61873414989478E-04
 0
 3.33485202181275E-04
 1.02295249664344E-03
 1.53810095801717E-04
 0
 5.6964863587332E-04
 0
 4.77063831140607E-05
 0
 2.64733375671645E-03
 1.39475849756615E-05
 5.45355983567477E-03
 4.80769230769231E-05
 1.30273330492482E-02
 3.82842125364882E-03

 0
 0
 1.61873414989478E-04
 0
 3.33485202181275E-04
 1.02295249664344E-03
 1.53810095801717E-04
 0
 5.6964863587332E-04
 0
 4.77063831140607E-05
 0
 2.64733375671645E-03
 1.39475849756615E-05
 5.45355983567477E-03
 4.80769230769231E-05
 1.30273330492482E-02
 3.82842125364882E-03

 0
 0
 0
 0
 1.50303189715504E-04
 0
 1.68458676354261E-04
 0
 0
 0
 0
 0
 0
 0
 0
 1.92307692307692E-04

 0
 0
 0
 0
 1.50303189715504E-04
 0
 1.68458676354261E-04
 0
 0
 0
 0
 0
 0
 0
 0
 1.92307692307692E-04

 0
 0
 0
 0
 9.39394935721902E-06
 0
 0
 0
 4.84807349679421E-05
 0
 0
 0
 0
 0
 0
 0
 1.21750776161198E-04

 0
 0
 0
 0
 9.39394935721902E-06
 0
 0
 0
 4.84807349679421E-05
 0
 0
 0
 0
 0
 0
 0
 1.21750776161198E-04

 0
 0
 0
 0
 5.63636961433141E-05
 0
 0
 0
 2.42403674839711E-05
 0
 0
 0
 0
 0
 0
 0
 4.05835920537327E-05

 0
 0
 0
 0
 5.63636961433141E-05
 0
 0
 0
 2.42403674839711E-05
 0
 0
 0
 0
 0
 0
 0
 4.05835920537327E-05

 0
 0
 0
 0
 0
 0
 0
 0
 0
 0
 0
 8.15278315635E-06

 0
 0
 0
 0
 0
 0
 0
 0
 0
 0
 0
 8.15278315635E-06

 0
 0
 5.4677242396446E-03
 1.44179096652424E-03
 1.24939526451013E-03
 6.88361784199646E-03
 5.34673190167873E-03
 0
 1.68470554013599E-03
 0
 1.06014184697913E-05
 0
 5.08773306883781E-03
 0
 7.90276335469039E-04
 3.55769230769231E-03
 1.07073043701765E-02
 .011708057871907

 0
 0
 5.4677242396446E-03
 1.44179096652424E-03
 1.24939526451013E-03
 6.88361784199646E-03
 5.34673190167873E-03
 0
 1.68470554013599E-03
 0
 1.06014184697913E-05
 0
 5.08773306883781E-03
 0
 7.90276335469039E-04
 3.55769230769231E-03
 1.07073043701765E-02
 .011708057871907

 0
 0
 5.4677242396446E-03
 1.44179096652424E-03
 1.24939526451013E-03
 6.88361784199646E-03
 5.34673190167873E-03
 0
 1.68470554013599E-03
 0
 1.06014184697913E-05
 0
 5.08773306883781E-03
 0
 7.90276335469039E-04
 3.55769230769231E-03
 1.07073043701765E-02
 .011708057871907

 0
 0
 5.4677242396446E-03
 1.44179096652424E-03
 1.24939526451013E-03
 6.88361784199646E-03
 5.34673190167873E-03
 0
 1.68470554013599E-03
 0
 1.06014184697913E-05
 0
 5.08773306883781E-03
 0
 7.90276335469039E-04
 3.55769230769231E-03
 1.07073043701765E-02
 .011708057871907

 0
 0
 5.4677242396446E-03
 1.44179096652424E-03
 1.24939526451013E-03
 6.88361784199646E-03
 5.34673190167873E-03
 0
 1.68470554013599E-03
 0
 1.06014184697913E-05
 0
 5.08773306883781E-03
 0
 7.90276335469039E-04
 3.55769230769231E-03
 1.07073043701765E-02
 .011708057871907

 0
 0
 1.34894512491232E-03
 2.20200802160065E-03
 3.22212462952612E-03
 1.64098629669885E-03
 7.26569595406205E-03
 0
 1.63016471329705E-03
 0
 8.48113477583301E-05
 8.19354707213175E-04
 1.04537572873035E-02
 7.43871198701945E-05
 3.18723017941232E-03
 7.66025641025641E-03
 1.36293229980452E-02
 1.63367191975775E-02

 0
 0
 1.13311390492635E-03
 1.95734046364502E-03
 2.91212430073789E-03
 1.04426400699018E-03
 6.03521518764832E-03
 0
 9.75674791229835E-04
 0
 4.77063831140607E-05
 8.0712553247865E-04
 1.04180789347871E-02
 7.43871198701945E-05
 3.18723017941232E-03
 3.92628205128205E-03
 7.90027258645996E-03
 1.61093782680259E-02

 0
 0
 1.09714203492869E-03
 1.52917223722267E-03
 2.23575994701812E-03
 9.59017965603222E-04
 4.50443851990742E-03
 0
 8.66593137551965E-04
 0
 1.06014184697913E-05
 4.11715549395675E-04
 5.70140073211979E-03
 0
 1.95936281521249E-03
 2.53205128205128E-03
 5.85756511975542E-03
 1.08896305255213E-02

 0
 0
 1.09714203492869E-03
 1.52917223722267E-03
 2.23575994701812E-03
 9.59017965603222E-04
 4.50443851990742E-03
 0
 8.66593137551965E-04
 0
 1.06014184697913E-05
 4.11715549395675E-04
 5.70140073211979E-03
 0
 1.95936281521249E-03
 2.53205128205128E-03
 5.85756511975542E-03
 1.08896305255213E-02

 0
 0
 1.09714203492869E-03
 1.52917223722267E-03
 2.18409322555342E-03
 9.59017965603222E-04
 4.50443851990742E-03
 0
 8.66593137551965E-04
 0
 1.06014184697913E-05
 4.11715549395675E-04
 5.70140073211979E-03
 0
 1.95936281521249E-03
 2.53205128205128E-03
 5.85756511975542E-03
 1.08896305255213E-02

 0
 0
 0
 0
 5.16667214647046E-05

 0
 0
 3.59718699976618E-05
 8.73812706984385E-05
 1.97272936501599E-04
 0
 4.68754577681423E-04
 0
 1.0908165367787E-04
 0
 3.71049646442694E-05
 3.6687524203575E-04
 1.52703348770167E-03
 7.43871198701945E-05
 4.50653447498873E-04
 3.36538461538462E-04
 6.01989948797035E-04
 4.11941764347486E-03

 0
 0
 0
 5.24287624190631E-05
 1.31515291001066E-04
 0
 4.17484545747517E-04
 0
 0
 0
 3.71049646442694E-05
 3.6687524203575E-04
 1.52703348770167E-03
 7.43871198701945E-05
 4.50653447498873E-04
 3.36538461538462E-04
 4.05835920537327E-04
 4.07849627615557E-03

 0
 0
 0
 5.24287624190631E-05
 0
 0
 0
 0
 0
 0
 0
 2.5273627784685E-04
 1.22019965606068E-03
 7.43871198701945E-05
 4.50653447498873E-04
 0
 0
 3.63745487282548E-03

 0
 0
 0
 0
 1.31515291001066E-04
 0
 4.17484545747517E-04
 0
 0
 0
 3.71049646442694E-05
 1.141389641889E-04
 3.0683383164099E-04
 0
 0
 3.36538461538462E-04
 4.05835920537327E-04
 4.4104140333009E-04

 0
 0
 3.59718699976618E-05
 0
 6.57576455005331E-05
 0
 5.12700319339056E-05
 0
 1.0908165367787E-04
 0
 0
 0
 0
 0
 0
 0
 1.96154028259708E-04
 2.27340929551593E-05

 0
 0
 3.59718699976618E-05
 0
 6.57576455005331E-05
 0
 5.12700319339056E-05
 0
 1.0908165367787E-04
 0
 0
 0
 0
 0
 0
 0
 1.96154028259708E-04
 2.27340929551593E-05

 0
 0
 0
 3.49525082793754E-05
 0
 0
 0
 0
 0
 0
 0
 0
 0
 0
 0
 0
 0
 1.81872743641274E-05

 0
 0
 0
 3.49525082793754E-05
 0
 0
 0
 0
 0
 0
 0
 0
 0
 0
 0
 0
 0
 1.81872743641274E-05

 0
 0
 0
 2.79620066235003E-04
 4.55606543825122E-04
 0
 9.30184865086573E-04
 0
 0
 0
 0
 1.63055663127E-05
 2.01939475242791E-03
 0
 2.08998700289333E-04
 7.37179487179487E-04
 5.68170288752257E-04
 6.77475970063746E-04

 0
 0
 0
 2.79620066235003E-04
 4.55606543825122E-04
 0
 9.30184865086573E-04
 0
 0
 0
 0
 1.63055663127E-05
 2.01939475242791E-03
 0
 2.08998700289333E-04
 7.37179487179487E-04
 5.68170288752257E-04
 6.77475970063746E-04

 0
 0
 0
 2.79620066235003E-04
 4.55606543825122E-04
 0
 9.30184865086573E-04
 0
 0
 0
 0
 1.63055663127E-05
 2.01939475242791E-03
 0
 2.08998700289333E-04
 7.37179487179487E-04
 5.68170288752257E-04
 6.77475970063746E-04

 0
 0
 0
 6.11668894889069E-05
 2.34848733930475E-05
 8.52460413869531E-05
 1.318372249729E-04
 0
 0
 0
 0
 1.2229174734525E-05
 1.17024996253773E-03
 0
 5.68215216411623E-04
 3.20512820512821E-04
 8.72547229155252E-04
 4.22854128965962E-04

 0
 0
 0
 6.11668894889069E-05
 2.34848733930475E-05
 8.52460413869531E-05
 1.318372249729E-04
 0
 0
 0
 0
 1.2229174734525E-05
 1.17024996253773E-03
 0
 5.68215216411623E-04
 3.20512820512821E-04
 8.72547229155252E-04
 4.22854128965962E-04

 0
 0
 0
 6.11668894889069E-05
 2.34848733930475E-05
 8.52460413869531E-05
 1.318372249729E-04
 0
 0
 0
 0
 1.2229174734525E-05
 1.17024996253773E-03
 0
 5.68215216411623E-04
 3.20512820512821E-04
 8.72547229155252E-04
 4.22854128965962E-04

 0
 0
 2.15831219985971E-04
 6.11668894889069E-05
 3.10000328788227E-04
 5.96722289708672E-04
 1.15723786365101E-03
 0
 6.54489922067219E-04
 0
 1.59021277046869E-05
 1.2229174734525E-05
 0
 0
 0
 3.73397435897436E-03
 5.72905041158526E-03
 3.1827730137223E-05

 0
 0
 2.15831219985971E-04
 6.11668894889069E-05
 3.10000328788227E-04
 5.96722289708672E-04
 1.15723786365101E-03
 0
 6.54489922067219E-04
 0
 1.59021277046869E-05
 1.2229174734525E-05
 0
 0
 0
 3.73397435897436E-03
 5.72905041158526E-03
 3.1827730137223E-05

 0
 0
 2.15831219985971E-04
 6.11668894889069E-05
 3.10000328788227E-04
 5.96722289708672E-04
 1.15723786365101E-03
 0
 6.54489922067219E-04
 0
 1.59021277046869E-05
 1.2229174734525E-05
 0
 0
 0
 3.73397435897436E-03
 5.72905041158526E-03
 3.1827730137223E-05

 0
 0
 2.15831219985971E-04
 6.11668894889069E-05
 3.10000328788227E-04
 5.96722289708672E-04
 1.15723786365101E-03
 0
 6.54489922067219E-04
 0
 1.59021277046869E-05
 0
 0
 0
 0
 3.73397435897436E-03
 5.72905041158526E-03
 3.1827730137223E-05

 0
 0
 0
 0
 0
 0
 0
 0
 0
 0
 0
 1.2229174734525E-05

 0
 0
 0
 1.83500668466721E-04
 0
 0
 7.32429027627223E-05
 0
 0
 0
 2.12028369395825E-05
 0
 3.56783525163942E-05
 0
 0
 0
 0
 1.9551319941437E-04

 0
 0
 0
 1.83500668466721E-04
 0
 0
 7.32429027627223E-05
 0
 0
 0
 2.12028369395825E-05
 0
 3.56783525163942E-05
 0
 0
 0
 0
 1.9551319941437E-04

 0
 0
 0
 1.83500668466721E-04
 0
 0
 7.32429027627223E-05
 0
 0
 0
 2.12028369395825E-05
 0
 3.56783525163942E-05
 0
 0
 0
 0
 1.9551319941437E-04

 0
 0
 0
 1.83500668466721E-04
 0
 0
 7.32429027627223E-05
 0
 0
 0
 2.12028369395825E-05
 0
 3.56783525163942E-05
 0
 0
 0
 0
 1.9551319941437E-04

 0
 0
 1.11512796992752E-03
 9.0876521526376E-04
 .015396682996482
 9.80329475949961E-04
 7.03131866522134E-03
 4.63783172091384E-05
 1.99983031742761E-03
 7.0098073577487E-05
 2.25280142483065E-03
 1.07249862421784E-02
 1.19950621160117E-02
 3.71935599350972E-05
 6.96880041277244E-03
 1.38621794871795E-02
 2.57705809541203E-03
 1.21490992752371E-02

 0
 0
 3.41732764977787E-04
 1.39810033117501E-04
 4.03939822360418E-03
 1.2786906208043E-04
 9.81454897020479E-04
 0
 3.27244961033609E-04
 0
 5.8307801583852E-05
 1.03051179096264E-02
 3.6748703091886E-03
 0
 4.89840703803123E-04
 9.13461538461538E-04
 5.54642424734347E-04
 1.81418061782171E-03

 0
 0
 3.41732764977787E-04
 1.39810033117501E-04
 4.03939822360418E-03
 1.2786906208043E-04
 9.81454897020479E-04
 0
 3.27244961033609E-04
 0
 5.8307801583852E-05
 1.03051179096264E-02
 3.6748703091886E-03
 0
 4.89840703803123E-04
 9.13461538461538E-04
 5.54642424734347E-04
 1.81418061782171E-03

 0
 0
 5.39578049964927E-05
 0
 6.01212758862017E-04
 0
 1.53810095801717E-04
 0
 2.06043123613754E-04
 0
 0
 1.02317428612192E-02
 3.38944348905745E-03
 0
 3.9840377242654E-04
 2.24358974358974E-04
 7.44032520985099E-05
 1.57774605108805E-03

 0
 0
 5.39578049964927E-05
 0
 6.01212758862017E-04
 0
 1.53810095801717E-04
 0
 2.06043123613754E-04
 0
 0
 1.02317428612192E-02
 3.38944348905745E-03
 0
 3.9840377242654E-04
 2.24358974358974E-04
 7.44032520985099E-05
 1.57774605108805E-03

 0
 0
 8.99296749941546E-05
 1.04857524838126E-04
 1.63454718815611E-03
 6.39345310402148E-05
 1.61134386077989E-04
 0
 8.48412861938987E-05
 0
 0
 0
 0
 0
 0
 0
 2.57029416340307E-04

 0
 0
 8.99296749941546E-05
 1.04857524838126E-04
 1.63454718815611E-03
 6.39345310402148E-05
 1.61134386077989E-04
 0
 8.48412861938987E-05
 0
 0
 0
 0
 0
 0
 0
 2.57029416340307E-04

 0
 0
 0
 3.49525082793754E-05
 1.17424366965238E-03
 0
 6.29888963759412E-04
 0
 2.42403674839711E-05
 0
 4.77063831140607E-05
 7.337504840715E-05
 2.85426820131154E-04
 0
 9.1436931376583E-05
 6.41025641025641E-04
 1.14986844152243E-04
 2.36434566733656E-04

 0
 0
 0
 3.49525082793754E-05
 1.17424366965238E-03
 0
 6.29888963759412E-04
 0
 2.42403674839711E-05
 0
 4.77063831140607E-05
 7.337504840715E-05
 2.85426820131154E-04
 0
 9.1436931376583E-05
 6.41025641025641E-04
 1.14986844152243E-04
 2.36434566733656E-04

 0
 0
 0
 0
 4.41515619789294E-04
 0
 3.66214513813611E-05

 0
 0
 0
 0
 3.8045494896737E-04
 0
 3.66214513813611E-05

 0
 0
 0
 0
 6.10606708219236E-05

 0
 0
 1.9784528498714E-04
 0
 1.40909240358285E-05
 6.39345310402148E-05
 0
 0
 1.21201837419855E-05
 0
 1.06014184697913E-05
 0
 0
 0
 0
 4.80769230769231E-05
 1.08222912143287E-04

 0
 0
 1.9784528498714E-04
 0
 1.40909240358285E-05
 6.39345310402148E-05
 0
 0
 1.21201837419855E-05
 0
 1.06014184697913E-05
 0
 0
 0
 0
 4.80769230769231E-05
 1.08222912143287E-04

 0
 0
 0
 0
 1.73788063108552E-04

 0
 0
 0
 0
 1.73788063108552E-04

 0
 0
 8.99296749941546E-05
 3.75739464003286E-04
 8.52970601635487E-03
 0
 1.64064102188498E-03
 0
 3.63605512259566E-05
 0
 1.06014184697913E-05
 0
 0
 0
 0
 2.08333333333333E-03

 0
 0
 8.99296749941546E-05
 3.75739464003286E-04
 8.52970601635487E-03
 0
 1.64064102188498E-03
 0
 3.63605512259566E-05
 0
 1.06014184697913E-05
 0
 0
 0
 0
 2.08333333333333E-03

 0
 0
 8.99296749941546E-05
 3.75739464003286E-04
 8.52970601635487E-03
 0
 1.64064102188498E-03
 0
 3.63605512259566E-05
 0
 1.06014184697913E-05
 0
 0
 0
 0
 2.08333333333333E-03

 0
 0
 8.99296749941546E-05
 3.75739464003286E-04
 8.52970601635487E-03
 0
 1.64064102188498E-03
 0
 3.63605512259566E-05
 0
 1.06014184697913E-05
 0
 0
 0
 0
 2.08333333333333E-03

 0
 0
 6.83465529955574E-04
 3.93215718142973E-04
 2.15121440280316E-03
 8.52460413869531E-04
 4.21146690885653E-03
 4.63783172091384E-05
 1.57562388645812E-03
 7.0098073577487E-05
 2.07257731084419E-03
 4.19868332552025E-04
 8.19174973776411E-03
 3.71935599350972E-05
 4.24528609962707E-03
 .010801282051282
 2.00888780665977E-03
 8.96177944292378E-03

 0
 0
 1.79859349988309E-04
 3.32048828654066E-04
 1.64863811219194E-03
 3.40984165547813E-04
 3.71341517007002E-03
 4.63783172091384E-05
 4.78747257808428E-04
 7.0098073577487E-05
 1.74923404751556E-04
 2.0381957890875E-04
 8.12039303273132E-03
 3.71935599350972E-05
 1.34542913311258E-03
 4.08653846153846E-03
 8.65783297146297E-04
 8.33886529595242E-03

 0
 0
 1.25901544991816E-04
 3.32048828654066E-04
 1.64863811219194E-03
 2.98361144854336E-04
 3.71341517007002E-03
 0
 1.03021561806877E-04
 0
 0
 2.0381957890875E-04
 8.12039303273132E-03
 0
 1.34542913311258E-03
 4.08653846153846E-03
 8.65783297146297E-04
 8.33886529595242E-03

 0
 0
 1.25901544991816E-04
 3.32048828654066E-04
 1.64863811219194E-03
 2.98361144854336E-04
 3.71341517007002E-03
 0
 1.03021561806877E-04
 0
 0
 2.0381957890875E-04
 8.12039303273132E-03
 0
 1.34542913311258E-03
 4.08653846153846E-03
 8.65783297146297E-04
 8.33886529595242E-03

 0
 0
 5.39578049964927E-05
 0
 0
 4.26230206934765E-05
 0
 4.63783172091384E-05
 3.75725696001552E-04
 7.0098073577487E-05
 1.74923404751556E-04
 0
 0
 3.71935599350972E-05

 0
 0
 5.39578049964927E-05
 0
 0
 0
 0
 4.63783172091384E-05
 1.57562388645812E-04
 7.0098073577487E-05
 1.59021277046869E-04
 0
 0
 3.71935599350972E-05

 0
 0
 0
 0
 0
 4.26230206934765E-05
 0
 0
 1.27261929290848E-04
 0
 1.59021277046869E-05

 0
 0
 0
 0
 0
 0
 0
 0
 9.09013780648915E-05

 0
 0
 5.03606179967265E-04
 6.11668894889069E-05
 4.60303518503732E-04
 3.62295675894551E-04
 4.46781706852606E-04
 0
 1.04839589368175E-03
 0
 1.37818440107286E-04
 6.1145873672625E-05
 0
 0
 2.86066971021024E-03
 6.71474358974359E-03
 8.11671841074653E-04
 5.4107141233279E-04

 0
 0
 0
 6.11668894889069E-05
 3.75757974288761E-04
 6.39345310402148E-05
 1.83107256906806E-04
 0
 8.48412861938986E-05
 0
 1.37818440107286E-04
 6.1145873672625E-05
 0
 0
 2.86066971021024E-03
 6.47435897435897E-03
 1.69098300223886E-04
 5.4107141233279E-04

 0
 0
 0
 0
 9.39394935721902E-06
 0
 6.59186124864501E-05
 0
 0
 0
 0
 0
 0
 0
 2.41654747209541E-03
 5.67307692307692E-03
 3.38196600447772E-05
 4.63775496285249E-04

 0
 0
 0
 0
 2.8181848071657E-05
 6.39345310402148E-05
 0
 0
 4.24206430969493E-05
 0
 0
 0
 0
 0
 2.80842003513791E-04
 8.01282051282051E-04
 1.35278640179109E-04

 0
 0
 0
 6.11668894889069E-05
 3.19394278145447E-04
 0
 1.17188644420356E-04

 0
 0
 0
 0
 1.8787898714438E-05
 0
 0
 0
 4.24206430969493E-05
 0
 0
 6.1145873672625E-05
 0
 0
 1.63280234601041E-04
 0
 0
 7.72959160475415E-05

 0
 0
 0
 0
 0
 0
 0
 0
 0
 0
 1.37818440107286E-04

 0
 0
 5.03606179967265E-04
 0
 8.45455442149711E-05
 2.98361144854336E-04
 2.636744499458E-04
 0
 9.63554607487849E-04
 0
 0
 0
 0
 0
 0
 2.40384615384615E-04
 6.42573540850767E-04

 0
 0
 3.77704634975449E-04
 0
 8.45455442149711E-05
 2.98361144854336E-04
 2.34377288840711E-04
 0
 5.33288084647363E-04
 0
 0
 0
 0
 0
 0
 2.40384615384615E-04
 5.20822764689569E-04

 0
 0
 1.25901544991816E-04
 0
 0
 0
 2.92971611050889E-05
 0
 4.30266522840486E-04
 0
 0
 0
 0
 0
 0
 0
 1.21750776161198E-04

 0
 0
 0
 0
 0
 0
 0
 0
 0
 0
 1.66442269975723E-03

 0
 0
 0
 0
 0
 0
 0
 0
 0
 0
 1.66442269975723E-03

 0
 0
 0
 0
 0
 0
 0
 0
 0
 0
 1.66442269975723E-03

 0
 0
 0
 0
 4.22727721074856E-05
 1.49180572427168E-04
 5.12700319339056E-05
 0
 1.81802756129783E-05
 0
 9.54127662281213E-05
 1.5490287997065E-04
 7.13567050327884E-05
 0
 3.91872563042499E-05
 0
 3.31432668438817E-04
 8.18427346385734E-05

 0
 0
 0
 0
 4.22727721074856E-05
 1.49180572427168E-04
 5.12700319339056E-05
 0
 1.81802756129783E-05
 0
 9.54127662281213E-05
 1.5490287997065E-04
 7.13567050327884E-05
 0
 3.91872563042499E-05
 0
 3.31432668438817E-04
 8.18427346385734E-05

 0
 0
 0
 0
 0
 1.49180572427168E-04
 0
 0
 0
 0
 9.54127662281213E-05
 1.5490287997065E-04
 7.13567050327884E-05
 0
 0
 0
 2.70557280358218E-04
 3.63745487282548E-05

 0
 0
 0
 0
 4.22727721074856E-05
 0
 5.12700319339056E-05
 0
 1.81802756129783E-05
 0
 0
 0
 0
 0
 3.91872563042499E-05
 0
 6.0875388080599E-05
 4.54681859103186E-05

 0
 0
 0
 0
 0
 0
 0
 0
 3.03004593549638E-05

 0
 0
 0
 0
 0
 0
 0
 0
 3.03004593549638E-05

 0
 0
 0
 0
 0
 0
 0
 0
 3.03004593549638E-05

 0
 0
 0
 0
 0
 0
 0
 0
 0
 0
 1.00713475463017E-04
 0
 1.28442069059019E-04
 0
 1.54136541463383E-03
 0
 1.35278640179109E-05
 6.22914146971364E-04

 0
 0
 0
 0
 0
 0
 0
 0
 0
 0
 0
 0
 1.28442069059019E-04
 0
 1.54136541463383E-03
 0
 0
 6.22914146971364E-04

 0
 0
 0
 0
 0
 0
 0
 0
 0
 0
 0
 0
 1.28442069059019E-04
 0
 1.54136541463383E-03
 0
 0
 6.22914146971364E-04

 0
 0
 0
 0
 0
 0
 0
 0
 0
 0
 0
 0
 1.28442069059019E-04
 0
 1.54136541463383E-03
 0
 0
 6.22914146971364E-04

 0
 0
 0
 0
 0
 0
 0
 0
 0
 0
 1.00713475463017E-04
 0
 0
 0
 0
 0
 1.35278640179109E-05

 0
 0
 0
 0
 0
 0
 0
 0
 0
 0
 7.42099292885388E-05
 0
 0
 0
 0
 0
 1.35278640179109E-05

 0
 0
 0
 0
 0
 0
 0
 0
 0
 0
 7.42099292885388E-05
 0
 0
 0
 0
 0
 1.35278640179109E-05

 0
 0
 0
 0
 0
 0
 0
 0
 0
 0
 2.65035461744781E-05

 0
 0
 0
 0
 0
 0
 0
 0
 0
 0
 2.65035461744781E-05

 0
 0
 0
 0
 6.52879480326722E-04
 0
 1.9775583745935E-04
 0
 6.06009187099276E-05
 0
 1.06014184697913E-05
 0
 0
 0
 6.92308194708414E-04
 6.41025641025641E-05
 0
 7.50225067520256E-04

 0
 0
 0
 0
 5.91818809504798E-04
 0
 1.09864354144083E-04
 0
 0
 0
 0
 0
 0
 0
 6.92308194708414E-04
 0
 0
 7.50225067520256E-04

 0
 0
 0
 0
 5.91818809504798E-04
 0
 1.09864354144083E-04
 0
 0
 0
 0
 0
 0
 0
 6.92308194708414E-04
 0
 0
 7.50225067520256E-04

 0
 0
 0
 0
 5.91818809504798E-04
 0
 1.09864354144083E-04
 0
 0
 0
 0
 0
 0
 0
 6.92308194708414E-04
 0
 0
 7.50225067520256E-04

 0
 0
 0
 0
 6.10606708219236E-05
 0
 8.78914833152667E-05
 0
 0
 0
 0
 0
 0
 0
 0
 6.41025641025641E-05

 0
 0
 0
 0
 3.28788227502666E-05
 0
 8.78914833152667E-05
 0
 0
 0
 0
 0
 0
 0
 0
 6.41025641025641E-05

 0
 0
 0
 0
 3.28788227502666E-05
 0
 8.78914833152667E-05
 0
 0
 0
 0
 0
 0
 0
 0
 6.41025641025641E-05

 0
 0
 0
 0
 2.8181848071657E-05

 0
 0
 0
 0
 2.8181848071657E-05

 0
 0
 0
 0
 0
 0
 0
 0
 4.24206430969493E-05

 0
 0
 0
 0
 0
 0
 0
 0
 4.24206430969493E-05

 0
 0
 0
 0
 0
 0
 0
 0
 4.24206430969493E-05

 0
 0
 0
 0
 0
 0
 0
 0
 1.81802756129783E-05

 0
 0
 0
 0
 0
 0
 0
 0
 1.81802756129783E-05

 0
 0
 0
 0
 0
 0
 0
 0
 1.81802756129783E-05

 0
 0
 0
 0
 0
 0
 0
 0
 0
 0
 1.06014184697913E-05

 0
 0
 0
 0
 0
 0
 0
 0
 0
 0
 1.06014184697913E-05

 0
 0
 0
 0
 0
 0
 0
 0
 0
 0
 1.06014184697913E-05

 0
 0
 0
 0
 2.34848733930475E-05

 0
 0
 0
 0
 2.34848733930475E-05

 0
 0
 0
 0
 2.34848733930475E-05

 0
 0
 0
 0
 2.34848733930475E-05

 0
 0
 2.69789024982464E-04
 1.03109899424157E-03
 2.01969911180209E-03
 8.73771924216269E-04
 .001318372249729
 0
 8.48412861938987E-04
 0
 1.59021277046869E-04
 4.80606567066832E-03
 1.43426977115905E-03
 9.06593023417995E-04
 9.25472369718701E-03
 5.44871794871795E-04
 2.83408751175233E-03
 5.79719370356562E-03

 0
 0
 2.69789024982464E-04
 1.03109899424157E-03
 2.01969911180209E-03
 8.73771924216269E-04
 .001318372249729
 0
 8.48412861938987E-04
 0
 1.59021277046869E-04
 4.80606567066832E-03
 1.43426977115905E-03
 9.06593023417995E-04
 9.25472369718701E-03
 5.44871794871795E-04
 2.83408751175233E-03
 5.79719370356562E-03

 0
 0
 2.69789024982464E-04
 1.03109899424157E-03
 2.01969911180209E-03
 8.73771924216269E-04
 .001318372249729
 0
 8.48412861938987E-04
 0
 1.59021277046869E-04
 4.80606567066832E-03
 1.43426977115905E-03
 9.06593023417995E-04
 9.25472369718701E-03
 5.44871794871795E-04
 2.83408751175233E-03
 5.79719370356562E-03

 0
 0
 2.69789024982464E-04
 1.03109899424157E-03
 2.01969911180209E-03
 8.73771924216269E-04
 .001318372249729
 0
 8.48412861938987E-04
 0
 1.59021277046869E-04
 4.80606567066832E-03
 1.43426977115905E-03
 9.06593023417995E-04
 9.25472369718701E-03
 5.44871794871795E-04
 2.83408751175233E-03
 5.79719370356562E-03

 0
 0
 2.69789024982464E-04
 1.03109899424157E-03
 2.01969911180209E-03
 8.73771924216269E-04
 .001318372249729
 0
 8.48412861938987E-04
 0
 1.59021277046869E-04
 4.80606567066832E-03
 1.43426977115905E-03
 9.06593023417995E-04
 9.25472369718701E-03
 5.44871794871795E-04
 2.83408751175233E-03
 5.79719370356562E-03

 0
 0
 1.79859349988309E-04
 3.9845859438488E-03
 2.48939657966304E-04
 7.67214372482578E-04
 9.1773357161691E-03
 2.31891586045692E-05
 1.27261929290848E-04
 0
 7.95106385234344E-05
 0
 0
 0
 0
 1.07371794871795E-03
 1.04840946138809E-03
 1.81872743641274E-05

 0
 0
 1.79859349988309E-04
 3.9845859438488E-03
 2.48939657966304E-04
 7.67214372482578E-04
 9.1773357161691E-03
 2.31891586045692E-05
 1.27261929290848E-04
 0
 7.95106385234344E-05
 0
 0
 0
 0
 1.07371794871795E-03
 1.04840946138809E-03
 1.81872743641274E-05

 0
 0
 1.79859349988309E-04
 3.9845859438488E-03
 2.48939657966304E-04
 7.67214372482578E-04
 9.1773357161691E-03
 2.31891586045692E-05
 1.27261929290848E-04
 0
 7.95106385234344E-05
 0
 0
 0
 0
 1.07371794871795E-03
 1.04840946138809E-03
 1.81872743641274E-05

 0
 0
 1.79859349988309E-04
 3.9845859438488E-03
 2.48939657966304E-04
 7.67214372482578E-04
 9.1773357161691E-03
 2.31891586045692E-05
 1.27261929290848E-04
 0
 7.95106385234344E-05
 0
 0
 0
 0
 1.07371794871795E-03
 1.04840946138809E-03
 1.81872743641274E-05

 0
 0
 1.79859349988309E-04
 3.9845859438488E-03
 2.48939657966304E-04
 7.67214372482578E-04
 9.1773357161691E-03
 2.31891586045692E-05
 1.27261929290848E-04
 0
 7.95106385234344E-05
 0
 0
 0
 0
 1.07371794871795E-03
 1.04840946138809E-03
 1.81872743641274E-05

 0
 0
 2.23025593985503E-03
 9.0876521526376E-04
 6.01212758862017E-03
 4.04918696588027E-03
 6.41607828201447E-03
 0
 1.61198443768407E-03
 0
 2.12028369395825E-05
 6.97062959867925E-04
 1.06321490498855E-03
 5.43025975052419E-03
 1.22133615481579E-03
 4.05448717948718E-03
 .011992451451878
 2.00969381723608E-03

 0
 0
 2.08636845986439E-03
 8.12645817495478E-04
 1.51242584651226E-03
 4.04918696588027E-03
 5.69829783493979E-03
 0
 1.16353763923061E-03
 0
 0
 6.97062959867925E-04
 1.06321490498855E-03
 5.43025975052419E-03
 1.22133615481579E-03
 3.63782051282051E-03
 1.12890025229466E-02
 2.00969381723608E-03

 0
 0
 1.54679040989946E-03
 4.63120734701724E-04
 3.89848898324589E-04
 2.79180785542271E-03
 3.68411800896493E-03
 0
 1.10293672052068E-03
 0
 0
 2.8534741047225E-05
 1.49849080568856E-04
 2.78951699513229E-05
 1.56749025216999E-04
 3.31730769230769E-03
 7.5756038500301E-03
 3.50105031509453E-04

 0
 0
 1.54679040989946E-03
 4.63120734701724E-04
 3.89848898324589E-04
 2.79180785542271E-03
 3.68411800896493E-03
 0
 1.10293672052068E-03
 0
 0
 2.8534741047225E-05
 1.49849080568856E-04
 2.78951699513229E-05
 1.56749025216999E-04
 3.31730769230769E-03
 7.5756038500301E-03
 3.50105031509453E-04

 0
 0
 1.54679040989946E-03
 4.63120734701724E-04
 3.89848898324589E-04
 2.79180785542271E-03
 3.68411800896493E-03
 0
 1.10293672052068E-03
 0
 0
 2.8534741047225E-05
 1.49849080568856E-04
 2.78951699513229E-05
 1.56749025216999E-04
 3.31730769230769E-03
 7.5756038500301E-03
 3.50105031509453E-04

 0
 0
 5.39578049964927E-04
 3.49525082793754E-04
 1.12257694818767E-03
 1.25737911045756E-03
 2.01417982597486E-03
 0
 6.06009187099276E-05
 0
 0
 6.685282188207E-04
 9.13365824419692E-04
 5.40236458057287E-03
 1.06458712959879E-03
 3.20512820512821E-04
 3.67957901287176E-03
 1.65958878572663E-03

 0
 0
 5.39578049964927E-04
 3.49525082793754E-04
 1.12257694818767E-03
 1.25737911045756E-03
 2.01417982597486E-03
 0
 6.06009187099276E-05
 0
 0
 6.685282188207E-04
 9.13365824419692E-04
 5.40236458057287E-03
 1.06458712959879E-03
 3.20512820512821E-04
 3.67957901287176E-03
 1.65958878572663E-03

 0
 0
 5.39578049964927E-04
 3.49525082793754E-04
 1.12257694818767E-03
 1.25737911045756E-03
 2.01417982597486E-03
 0
 6.06009187099276E-05
 0
 0
 6.685282188207E-04
 9.13365824419692E-04
 5.40236458057287E-03
 1.06458712959879E-03
 3.20512820512821E-04
 3.67957901287176E-03
 1.65958878572663E-03

 0
 0
 0
 0
 0
 0
 0
 0
 0
 0
 0
 0
 0
 0
 0
 0
 3.38196600447772E-05

 0
 0
 0
 0
 0
 0
 0
 0
 0
 0
 0
 0
 0
 0
 0
 0
 3.38196600447772E-05

 0
 0
 0
 0
 0
 0
 0
 0
 0
 0
 0
 0
 0
 0
 0
 0
 3.38196600447772E-05

 0
 0
 1.43887479990647E-04
 9.61193977682823E-05
 4.41045922321433E-03
 0
 2.41701579116984E-04
 0
 4.24206430969493E-04
 0
 0
 0
 0
 0
 0
 6.41025641025641E-05
 1.42042572188064E-04

 0
 0
 1.43887479990647E-04
 9.61193977682823E-05
 4.41045922321433E-03
 0
 2.41701579116984E-04
 0
 4.24206430969493E-04
 0
 0
 0
 0
 0
 0
 6.41025641025641E-05
 1.42042572188064E-04

 0
 0
 1.43887479990647E-04
 9.61193977682823E-05
 4.41045922321433E-03
 0
 2.41701579116984E-04
 0
 4.24206430969493E-04
 0
 0
 0
 0
 0
 0
 6.41025641025641E-05
 1.42042572188064E-04

 0
 0
 1.43887479990647E-04
 9.61193977682823E-05
 4.41045922321433E-03
 0
 2.41701579116984E-04
 0
 3.81785787872544E-04
 0
 0
 0
 0
 0
 0
 6.41025641025641E-05
 1.42042572188064E-04

 0
 0
 0
 0
 0
 0
 0
 0
 4.24206430969493E-05

 0
 0
 0
 0
 0
 0
 4.76078867957695E-04
 0
 0
 0
 2.12028369395825E-05
 0
 0
 0
 0
 3.52564102564103E-04
 5.47878492725391E-04

 0
 0
 0
 0
 0
 0
 4.76078867957695E-04
 0
 0
 0
 2.12028369395825E-05
 0
 0
 0
 0
 3.52564102564103E-04
 5.47878492725391E-04

 0
 0
 0
 0
 0
 0
 4.76078867957695E-04
 0
 0
 0
 2.12028369395825E-05
 0
 0
 0
 0
 3.52564102564103E-04
 5.47878492725391E-04

 0
 0
 0
 0
 0
 0
 4.76078867957695E-04
 0
 0
 0
 2.12028369395825E-05
 0
 0
 0
 0
 3.52564102564103E-04
 5.47878492725391E-04

 0
 0
 0
 0
 8.92425188935806E-05

 0
 0
 0
 0
 8.92425188935806E-05

 0
 0
 0
 0
 8.92425188935806E-05

 0
 0
 0
 0
 8.92425188935806E-05

 0
 0
 0
 0
 0
 0
 0
 0
 2.42403674839711E-05

 0
 0
 0
 0
 0
 0
 0
 0
 2.42403674839711E-05

 0
 0
 0
 0
 0
 0
 0
 0
 2.42403674839711E-05

 0
 0
 0
 0
 0
 0
 0
 0
 2.42403674839711E-05

 0
 0
 0
 0
 0
 0
 0
 0
 0
 0
 0
 0
 0
 0
 0
 0
 1.35278640179109E-05

 0
 0
 0
 0
 0
 0
 0
 0
 0
 0
 0
 0
 0
 0
 0
 0
 1.35278640179109E-05

 0
 0
 0
 0
 0
 0
 0
 0
 0
 0
 0
 0
 0
 0
 0
 0
 1.35278640179109E-05

 0
 0
 0
 0
 0
 0
 0
 0
 0
 0
 0
 0
 0
 0
 0
 0
 1.35278640179109E-05

 0
 0
 5.75549919962589E-04
 4.45644480562036E-04
 2.60682094662828E-03
 5.11476248321718E-04
 3.8232795242141E-03
 5.10161489300522E-05
 6.26007490273552E-03
 0
 1.16615603167704E-03
 0
 0
 6.97379248783073E-05
 1.1756176891275E-03
 6.36217948717949E-03
 7.30504656967189E-04
 4.54681859103186E-05

 0
 0
 3.7770463497545E-04
 1.48548160187346E-04
 1.69560785897803E-03
 3.19672655201074E-04
 1.83839685934433E-03
 5.10161489300522E-05
 4.3572060552438E-03
 0
 1.02303688233486E-03
 0
 0
 6.97379248783073E-05
 3.26560469202082E-05
 5.2724358974359E-03
 7.30504656967189E-04

 0
 0
 3.05760894980126E-04
 7.86431436285947E-05
 1.34333475808232E-03
 3.19672655201074E-04
 1.21583218586119E-03
 0
 1.63016471329705E-03
 0
 1.00713475463017E-04
 0
 0
 0
 0
 5.16025641025641E-03
 5.54642424734347E-04

 0
 0
 3.05760894980126E-04
 7.86431436285947E-05
 1.34333475808232E-03
 3.19672655201074E-04
 1.21583218586119E-03
 0
 1.63016471329705E-03
 0
 1.00713475463017E-04
 0
 0
 0
 0
 5.16025641025641E-03
 5.54642424734347E-04

 0
 0
 3.05760894980126E-04
 7.86431436285947E-05
 1.34333475808232E-03
 3.19672655201074E-04
 1.21583218586119E-03
 0
 1.63016471329705E-03
 0
 1.00713475463017E-04
 0
 0
 0
 0
 5.16025641025641E-03
 5.54642424734347E-04

 0
 0
 7.19437399953237E-05
 6.99050165587508E-05
 2.48939657966304E-04
 0
 3.14944481879706E-04
 0
 2.07255141987953E-03
 0
 3.3924539103332E-04
 0
 0
 0
 3.26560469202082E-05
 0
 1.75862232232842E-04

 0
 0
 7.19437399953237E-05
 6.99050165587508E-05
 2.48939657966304E-04
 0
 3.14944481879706E-04
 0
 2.07255141987953E-03
 0
 3.3924539103332E-04
 0
 0
 0
 3.26560469202082E-05
 0
 1.75862232232842E-04

 0
 0
 7.19437399953237E-05
 6.99050165587508E-05
 2.48939657966304E-04
 0
 3.14944481879706E-04
 0
 2.07255141987953E-03
 0
 3.3924539103332E-04
 0
 0
 0
 3.26560469202082E-05
 0
 1.75862232232842E-04

 0
 0
 0
 0
 3.75757974288761E-05
 0
 0
 0
 1.99983031742761E-04
 0
 4.45259575731233E-04

 0
 0
 0
 0
 3.75757974288761E-05
 0
 0
 0
 1.99983031742761E-04
 0
 4.45259575731233E-04

 0
 0
 0
 0
 3.75757974288761E-05
 0
 0
 0
 1.99983031742761E-04
 0
 4.45259575731233E-04

 0
 0
 0
 0
 5.16667214647046E-05
 0
 3.07620191603434E-04
 0
 1.51502296774819E-04
 0
 0
 0
 0
 0
 0
 1.12179487179487E-04

 0
 0
 0
 0
 5.16667214647046E-05
 0
 3.07620191603434E-04
 0
 1.51502296774819E-04
 0
 0
 0
 0
 0
 0
 1.12179487179487E-04

 0
 0
 0
 0
 5.16667214647046E-05
 0
 3.07620191603434E-04
 0
 1.51502296774819E-04
 0
 0
 0
 0
 0
 0
 1.12179487179487E-04

 0
 0
 0
 0
 1.40909240358285E-05
 0
 0
 5.10161489300522E-05
 3.03004593549638E-04
 0
 1.37818440107286E-04
 0
 0
 6.97379248783073E-05

 0
 0
 0
 0
 1.40909240358285E-05
 0
 0
 5.10161489300522E-05
 3.03004593549638E-04
 0
 1.37818440107286E-04
 0
 0
 6.97379248783073E-05

 0
 0
 0
 0
 1.40909240358285E-05
 0
 0
 5.10161489300522E-05
 3.03004593549638E-04
 0
 1.37818440107286E-04
 0
 0
 6.97379248783073E-05

 0
 0
 1.07915609992985E-04
 1.66024414327033E-04
 7.13940151148646E-04
 1.06557551733691E-04
 1.11329212199338E-03
 0
 4.36326614711479E-04
 0
 9.54127662281213E-05
 0
 0
 0
 1.14296164220729E-03
 1.04166666666667E-03
 0
 4.54681859103186E-05

 0
 0
 1.07915609992985E-04
 6.99050165587508E-05
 6.81061328398379E-04
 1.06557551733691E-04
 9.37509155362845E-04
 0
 4.18146339098501E-04
 0
 4.2405673879165E-05
 0
 0
 0
 1.14296164220729E-03
 9.13461538461538E-04
 0
 4.54681859103186E-05

 0
 0
 1.07915609992985E-04
 6.99050165587508E-05
 6.81061328398379E-04
 1.06557551733691E-04
 9.37509155362845E-04
 0
 4.18146339098501E-04
 0
 4.2405673879165E-05
 0
 0
 0
 1.14296164220729E-03
 9.13461538461538E-04
 0
 4.54681859103186E-05

 0
 0
 1.07915609992985E-04
 6.99050165587508E-05
 6.81061328398379E-04
 1.06557551733691E-04
 9.37509155362845E-04
 0
 4.18146339098501E-04
 0
 4.2405673879165E-05
 0
 0
 0
 1.14296164220729E-03
 9.13461538461538E-04
 0
 4.54681859103186E-05

 0
 0
 0
 9.61193977682823E-05
 3.28788227502666E-05
 0
 1.75782966630533E-04
 0
 0
 0
 4.2405673879165E-05
 0
 0
 0
 0
 1.28205128205128E-04

 0
 0
 0
 9.61193977682823E-05
 3.28788227502666E-05
 0
 1.75782966630533E-04
 0
 0
 0
 4.2405673879165E-05
 0
 0
 0
 0
 1.28205128205128E-04

 0
 0
 0
 9.61193977682823E-05
 3.28788227502666E-05
 0
 1.75782966630533E-04
 0
 0
 0
 4.2405673879165E-05
 0
 0
 0
 0
 1.28205128205128E-04

 0
 0
 0
 0
 0
 0
 0
 0
 1.81802756129783E-05
 0
 1.06014184697913E-05

 0
 0
 0
 0
 0
 0
 0
 0
 1.81802756129783E-05
 0
 1.06014184697913E-05

 0
 0
 0
 0
 0
 0
 0
 0
 1.81802756129783E-05
 0
 1.06014184697913E-05

 0
 0
 8.99296749941546E-05
 1.31071906047657E-04
 1.972729365016E-04
 0
 8.42293381771306E-04
 0
 1.12717708800465E-03
 0
 2.12028369395825E-05
 0
 0
 0
 0
 4.80769230769231E-05

 0
 0
 8.99296749941546E-05
 1.04857524838126E-04
 4.22727721074856E-05
 0
 8.42293381771306E-04
 0
 1.10293672052068E-03
 0
 2.12028369395825E-05

 0
 0
 8.99296749941546E-05
 1.04857524838126E-04
 4.22727721074856E-05
 0
 8.42293381771306E-04
 0
 1.10293672052068E-03
 0
 2.12028369395825E-05

 0
 0
 8.99296749941546E-05
 1.04857524838126E-04
 4.22727721074856E-05
 0
 8.42293381771306E-04
 0
 1.10293672052068E-03
 0
 2.12028369395825E-05

 0
 0
 0
 2.62143812095315E-05
 1.55000164394114E-04
 0
 0
 0
 0
 0
 0
 0
 0
 0
 0
 4.80769230769231E-05

 0
 0
 0
 2.62143812095315E-05
 1.55000164394114E-04
 0
 0
 0
 0
 0
 0
 0
 0
 0
 0
 4.80769230769231E-05

 0
 0
 0
 2.62143812095315E-05
 1.55000164394114E-04
 0
 0
 0
 0
 0
 0
 0
 0
 0
 0
 4.80769230769231E-05

 0
 0
 0
 0
 0
 0
 0
 0
 2.42403674839711E-05

 0
 0
 0
 0
 0
 0
 0
 0
 2.42403674839711E-05

 0
 0
 0
 0
 0
 0
 0
 0
 2.42403674839711E-05

 0
 0
 0
 0
 0
 8.52460413869531E-05
 0
 0
 3.39365144775595E-04
 0
 2.65035461744782E-05

 0
 0
 0
 0
 0
 8.52460413869531E-05
 0
 0
 3.39365144775595E-04
 0
 2.65035461744782E-05

 0
 0
 0
 0
 0
 8.52460413869531E-05
 0
 0
 2.24223399226732E-04
 0
 1.06014184697913E-05

 0
 0
 0
 0
 0
 8.52460413869531E-05
 0
 0
 2.24223399226732E-04
 0
 1.06014184697913E-05

 0
 0
 0
 0
 0
 0
 0
 0
 1.15141745548863E-04
 0
 1.59021277046869E-05

 0
 0
 0
 0
 0
 0
 0
 0
 1.15141745548863E-04
 0
 1.59021277046869E-05

 0
 0
 0
 0
 0
 0
 2.92971611050889E-05

 0
 0
 0
 0
 0
 0
 2.92971611050889E-05

 0
 0
 0
 0
 0
 0
 2.92971611050889E-05

 0
 0
 0
 0
 0
 0
 2.92971611050889E-05

 0
 0
 1.04318422993219E-03
 0
 7.98485695363616E-05
 1.74754384843254E-03
 2.19728708288167E-04
 0
 3.81785787872545E-03
 0
 1.37818440107286E-04
 0
 0
 2.66863792534323E-03
 0
 9.93589743589743E-04
 1.44748144991647E-03

 0
 0
 2.33817154984802E-04
 0
 5.16667214647046E-05
 8.52460413869531E-05
 0
 0
 3.62999503072467E-03
 0
 0
 0
 0
 2.64539195038379E-03

 0
 0
 2.33817154984802E-04
 0
 5.16667214647046E-05
 8.52460413869531E-05
 0
 0
 3.62999503072467E-03
 0
 0
 0
 0
 2.64539195038379E-03

 0
 0
 2.33817154984802E-04
 0
 5.16667214647046E-05
 8.52460413869531E-05
 0
 0
 3.62999503072467E-03
 0
 0
 0
 0
 2.64539195038379E-03

 0
 0
 2.33817154984802E-04
 0
 5.16667214647046E-05
 8.52460413869531E-05
 0
 0
 3.62999503072467E-03
 0
 0
 0
 0
 2.64539195038379E-03

 0
 0
 8.09367074947391E-04
 0
 0
 1.66229780704559E-03
 1.61134386077989E-04
 0
 1.87862848000776E-04
 0
 0
 0
 0
 2.32459749594358E-05
 0
 1.76282051282051E-04
 1.44748144991647E-03

 0
 0
 8.09367074947391E-04
 0
 0
 1.66229780704559E-03
 1.61134386077989E-04
 0
 1.87862848000776E-04
 0
 0
 0
 0
 2.32459749594358E-05
 0
 1.76282051282051E-04
 1.44748144991647E-03

 0
 0
 8.09367074947391E-04
 0
 0
 1.66229780704559E-03
 1.61134386077989E-04
 0
 1.87862848000776E-04
 0
 0
 0
 0
 2.32459749594358E-05
 0
 1.76282051282051E-04
 1.44748144991647E-03

 0
 0
 8.09367074947391E-04
 0
 0
 1.66229780704559E-03
 1.61134386077989E-04
 0
 1.87862848000776E-04
 0
 0
 0
 0
 2.32459749594358E-05
 0
 1.76282051282051E-04
 1.44748144991647E-03

 0
 0
 0
 0
 2.8181848071657E-05
 0
 5.85943222101778E-05
 0
 0
 0
 1.37818440107286E-04
 0
 0
 0
 0
 8.17307692307692E-04

 0
 0
 0
 0
 2.8181848071657E-05
 0
 5.85943222101778E-05
 0
 0
 0
 3.18042554093738E-05
 0
 0
 0
 0
 8.17307692307692E-04

 0
 0
 0
 0
 2.8181848071657E-05
 0
 5.85943222101778E-05
 0
 0
 0
 3.18042554093738E-05
 0
 0
 0
 0
 8.17307692307692E-04

 0
 0
 0
 0
 2.8181848071657E-05
 0
 5.85943222101778E-05
 0
 0
 0
 3.18042554093738E-05
 0
 0
 0
 0
 8.17307692307692E-04

 0
 0
 0
 0
 0
 0
 0
 0
 0
 0
 6.36085108187475E-05

 0
 0
 0
 0
 0
 0
 0
 0
 0
 0
 6.36085108187475E-05

 0
 0
 0
 0
 0
 0
 0
 0
 0
 0
 6.36085108187475E-05

 0
 0
 0
 0
 0
 0
 0
 0
 0
 0
 4.2405673879165E-05

 0
 0
 0
 0
 0
 0
 0
 0
 0
 0
 4.2405673879165E-05

 0
 0
 0
 0
 0
 0
 0
 0
 0
 0
 4.2405673879165E-05

 0
 0
 2.57198870483282E-03
 8.12645817495478E-04
 1.82571405757551E-02
 2.47213520022164E-03
 3.22268772155978E-03
 4.03491359719504E-04
 1.32110002787642E-03
 3.56862920030843E-04
 3.16452341323269E-03
 6.033059535699E-04
 2.19778651500988E-03
 2.92899284488892E-04
 9.60087779454121E-04
 1.36217948717949E-03
 4.21392964157924E-03
 2.34615839297243E-03

 0
 0
 2.5000449648375E-03
 6.90312038517664E-04
 8.22440266224524E-03
 2.47213520022164E-03
 2.9516889813377E-03
 9.27566344182768E-06
 9.69614699358843E-04
 1.91176564302237E-05
 1.40468794724734E-03
 0
 2.14070115098365E-05
 9.29838998377431E-06
 0
 1.36217948717949E-03
 4.10570672943596E-03
 2.00060018005402E-04

 0
 0
 2.51803089983633E-04
 9.61193977682823E-05
 3.51803403427852E-03
 3.40984165547812E-04
 4.24808836023789E-04
 0
 3.69665604130559E-04
 0
 5.14168795784876E-04
 0
 0
 0
 0
 1.76282051282051E-04
 2.29973688304485E-04

 0
 0
 2.51803089983633E-04
 9.61193977682823E-05
 3.51803403427852E-03
 3.40984165547812E-04
 4.24808836023789E-04
 0
 1.57562388645812E-04
 0
 0
 0
 0
 0
 0
 1.76282051282051E-04
 2.29973688304485E-04

 0
 0
 2.51803089983633E-04
 9.61193977682823E-05
 3.51803403427852E-03
 3.40984165547812E-04
 4.24808836023789E-04
 0
 1.57562388645812E-04
 0
 0
 0
 0
 0
 0
 1.76282051282051E-04
 2.29973688304485E-04

 0
 0
 0
 0
 0
 0
 0
 0
 2.12103215484747E-04
 0
 5.14168795784876E-04

 0
 0
 0
 0
 0
 0
 0
 0
 2.12103215484747E-04
 0
 5.14168795784876E-04

 0
 0
 2.24824187485386E-03
 5.94192640749382E-04
 4.70636862796672E-03
 2.13115103467383E-03
 2.52688014531392E-03
 9.27566344182768E-06
 5.99949095228284E-04
 1.91176564302237E-05
 8.90519151462465E-04
 0
 2.14070115098365E-05
 9.29838998377431E-06
 0
 1.18589743589744E-03
 3.87573304113147E-03
 2.00060018005402E-04

 0
 0
 1.78060756488426E-03
 2.79620066235003E-04
 1.40909240358285E-03
 1.49180572427168E-03
 1.72120821492397E-03
 0
 3.69665604130559E-04
 0
 1.53720567811973E-04
 0
 0
 0
 0
 8.33333333333333E-04
 3.10464479211055E-03
 1.31857739139924E-04

 0
 0
 1.78060756488426E-03
 2.79620066235003E-04
 1.40909240358285E-03
 1.49180572427168E-03
 1.72120821492397E-03
 0
 2.36343582968718E-04
 0
 0
 0
 0
 0
 0
 8.33333333333333E-04
 3.10464479211055E-03
 1.31857739139924E-04

 0
 0
 0
 0
 0
 0
 0
 0
 1.33322021161841E-04
 0
 1.53720567811973E-04

 0
 0
 0
 0
 1.59227441604862E-03
 0
 5.12700319339056E-05
 0
 0
 1.91176564302237E-05
 0
 0
 0
 0
 0
 0
 8.79311161164208E-05

 0
 0
 0
 0
 1.59227441604862E-03
 0
 5.12700319339056E-05
 0
 0
 1.91176564302237E-05
 0
 0
 0
 0
 0
 0
 8.79311161164208E-05

 0
 0
 0
 0
 8.87728214257197E-04
 0
 6.59186124864501E-05
 0
 5.45408268389349E-05

 0
 0
 0
 0
 8.87728214257197E-04
 0
 6.59186124864501E-05
 0
 5.45408268389349E-05

 0
 0
 4.67634309969603E-04
 3.14572574514379E-04
 8.17273594078054E-04
 6.39345310402148E-04
 6.8848328596959E-04
 9.27566344182768E-06
 1.7574266425879E-04
 0
 7.36798583650492E-04
 0
 2.14070115098365E-05
 9.29838998377431E-06
 0
 3.52564102564103E-04
 6.83157132904501E-04
 6.82022788654778E-05

 0
 0
 1.79859349988309E-04
 1.04857524838126E-04
 9.39394935721902E-05
 4.26230206934765E-04
 5.41997480444145E-04
 9.27566344182768E-06
 1.7574266425879E-04
 0
 7.36798583650492E-04
 0
 0
 9.29838998377431E-06
 0
 3.20512820512821E-04
 3.44960532456728E-04

 0
 0
 1.25901544991816E-04
 2.62143812095315E-05
 4.32121670432075E-04
 0
 0
 0
 0
 0
 0
 0
 0
 0
 0
 0
 1.01458980134332E-04

 0
 0
 1.61873414989478E-04
 1.83500668466721E-04
 2.91212430073789E-04
 2.13115103467383E-04
 1.46485805525445E-04
 0
 0
 0
 0
 0
 2.14070115098365E-05
 0
 0
 3.20512820512821E-05
 2.36737620313441E-04
 6.82022788654778E-05

 0
 0
 7.19437399953236E-05
 1.22333778977814E-04
 8.64713038332011E-03
 0
 2.70998740222072E-04
 3.94215696277676E-04
 3.33305052904602E-04
 3.37745263600619E-04
 1.73863262904577E-03
 6.033059535699E-04
 2.17637950350005E-03
 2.83600894505117E-04
 9.60087779454121E-04
 0
 7.44032520985099E-05
 2.14609837496703E-03

 0
 0
 3.59718699976618E-05
 3.49525082793754E-05
 2.41424498480528E-03
 0
 6.59186124864501E-05
 1.85513268836554E-04
 0
 1.72058907872013E-04
 7.31497874415597E-04
 1.467500968143E-04
 3.21105172647548E-04
 1.06931484813405E-04
 4.57184656882915E-05
 0
 2.02917960268663E-05
 2.86449571235007E-04

 0
 0
 3.59718699976618E-05
 0
 2.41424498480528E-03
 0
 6.59186124864501E-05
 1.85513268836554E-04
 0
 1.33823595011566E-04
 7.31497874415597E-04
 1.467500968143E-04
 2.99698161137711E-04
 1.06931484813405E-04
 4.57184656882915E-05
 0
 2.02917960268663E-05
 2.86449571235007E-04

 0
 0
 3.59718699976618E-05
 0
 2.34379036462614E-03
 0
 6.59186124864501E-05
 1.85513268836554E-04
 0
 1.33823595011566E-04
 7.31497874415597E-04
 1.467500968143E-04
 2.99698161137711E-04
 1.06931484813405E-04
 4.57184656882915E-05
 0
 2.02917960268663E-05
 2.86449571235007E-04

 0
 0
 0
 0
 7.04546201791426E-05

 0
 0
 0
 3.49525082793754E-05
 0
 0
 0
 0
 0
 3.82353128604475E-05
 0
 0
 2.14070115098365E-05

 0
 0
 0
 3.49525082793754E-05
 0
 0
 0
 0
 0
 3.82353128604475E-05
 0
 0
 2.14070115098365E-05

 0
 0
 0
 3.49525082793754E-05
 2.92151825009512E-03
 0
 1.68458676354261E-04
 3.24648220463969E-05
 1.03021561806877E-04
 9.55882821511187E-05
 4.2405673879165E-04
 3.6687524203575E-05
 7.84923755360672E-05
 6.97379248783073E-05
 0
 0
 5.41114560716436E-05

 0
 0
 0
 0
 2.32969944059032E-03
 0
 1.68458676354261E-04
 0
 0
 0
 0
 3.6687524203575E-05
 0
 0
 0
 0
 5.41114560716436E-05

 0
 0
 0
 0
 2.32969944059032E-03
 0
 1.68458676354261E-04
 0
 0
 0
 0
 3.6687524203575E-05
 0
 0
 0
 0
 5.41114560716436E-05

 0
 0
 0
 3.49525082793754E-05
 5.91818809504798E-04
 0
 0
 3.24648220463969E-05
 0
 9.55882821511187E-05
 4.2405673879165E-04
 0
 7.84923755360672E-05
 6.97379248783073E-05

 0
 0
 0
 3.49525082793754E-05
 5.91818809504798E-04
 0
 0
 3.24648220463969E-05
 0
 9.55882821511187E-05
 4.2405673879165E-04
 0
 7.84923755360672E-05
 6.97379248783073E-05

 0
 0
 0
 0
 0
 0
 0
 0
 1.03021561806877E-04

 0
 0
 0
 0
 0
 0
 0
 0
 1.03021561806877E-04

 0
 0
 3.59718699976618E-05
 5.24287624190631E-05
 1.79424432722883E-03
 0
 3.66214513813611E-05
 1.06670129581018E-04
 1.69682572387798E-04
 7.0098073577487E-05
 5.4597305119425E-04
 3.70951633613925E-04
 7.84923755360672E-04
 1.06931484813405E-04
 8.7518205746158E-04
 0
 0
 1.85964880373203E-03

 0
 0
 0
 5.24287624190631E-05
 1.60166836540584E-03
 0
 3.66214513813611E-05
 0
 1.0908165367787E-04
 0
 1.90825532456243E-04
 2.89423802050425E-04
 7.06431379824605E-04
 1.06931484813405E-04
 8.7518205746158E-04
 0
 0
 1.79599334345758E-03

 0
 0
 0
 5.24287624190631E-05
 8.40758467471102E-04
 0
 3.66214513813611E-05
 0
 0
 0
 9.54127662281213E-05
 2.89423802050425E-04
 7.06431379824605E-04
 1.06931484813405E-04
 6.85776985324372E-04
 0
 0
 1.79599334345758E-03

 0
 0
 0
 0
 6.95152252434207E-04
 0
 0
 0
 0
 0
 7.42099292885388E-05

 0
 0
 0
 0
 6.57576455005331E-05
 0
 0
 0
 0
 0
 0
 0
 0
 0
 1.89405072137208E-04

 0
 0
 0
 0
 0
 0
 0
 0
 1.0908165367787E-04

 0
 0
 0
 0
 0
 0
 0
 0
 0
 0
 2.12028369395825E-05

 0
 0
 3.59718699976618E-05
 0
 0
 0
 0
 1.06670129581018E-04
 6.06009187099276E-05
 7.0098073577487E-05
 3.55147518738007E-04
 8.15278315635E-05
 7.84923755360672E-05
 0
 0
 0
 0
 6.3655460274446E-05

 0
 0
 0
 0
 0
 0
 0
 1.06670129581018E-04
 0
 7.0098073577487E-05
 3.23343263328633E-04
 8.15278315635E-05
 7.84923755360672E-05
 0
 0
 0
 0
 6.3655460274446E-05

 0
 0
 0
 0
 0
 0
 0
 0
 6.06009187099276E-05
 0
 3.18042554093738E-05

 0
 0
 3.59718699976618E-05

 0
 0
 0
 0
 1.9257596182299E-04

 0
 0
 0
 0
 1.9257596182299E-04

 0
 0
 0
 0
 1.35272870743954E-03
 0
 0
 0
 0
 0
 0
 0
 5.70853640262307E-05
 0
 3.91872563042499E-05

 0
 0
 0
 0
 1.35272870743954E-03
 0
 0
 0
 0
 0
 0
 0
 5.70853640262307E-05
 0
 3.91872563042499E-05

 0
 0
 0
 0
 1.35272870743954E-03
 0
 0
 0
 0
 0
 0
 0
 5.70853640262307E-05
 0
 3.91872563042499E-05

 0
 0
 0
 0
 1.64394113751333E-04
 0
 0
 6.95674758137076E-05
 0
 0
 0
 4.89166989381E-05
 9.34772835929528E-04

 0
 0
 0
 0
 1.64394113751333E-04
 0
 0
 6.95674758137076E-05
 0
 0
 0
 4.89166989381E-05
 8.56280460393461E-04

 0
 0
 0
 0
 0
 0
 0
 0
 0
 0
 0
 0
 6.1366766328198E-04

 0
 0
 0
 0
 0
 0
 0
 0
 0
 0
 0
 4.89166989381E-05
 2.42612797111481E-04

 0
 0
 0
 0
 1.64394113751333E-04

 0
 0
 0
 0
 0
 0
 0
 6.95674758137076E-05

 0
 0
 0
 0
 0
 0
 0
 0
 0
 0
 0
 0
 7.84923755360672E-05

 0
 0
 0
 0
 0
 0
 0
 0
 0
 0
 0
 0
 7.84923755360672E-05

 0
 0
 0
 0
 0
 0
 0
 0
 6.06009187099276E-05
 0
 2.65035461744781E-05

 0
 0
 0
 0
 0
 0
 0
 0
 6.06009187099276E-05
 0
 2.65035461744781E-05

 0
 0
 0
 0
 0
 0
 0
 0
 6.06009187099276E-05
 0
 2.65035461744781E-05

 0
 0
 0
 0
 0
 0
 0
 0
 0
 0
 1.06014184697913E-05

 0
 0
 0
 0
 0
 0
 0
 0
 0
 0
 1.06014184697913E-05

 0
 0
 0
 0
 0
 0
 0
 0
 0
 0
 1.06014184697913E-05

 0
 0
 0
 0
 1.32924383404649E-03
 0
 0
 0
 1.81802756129783E-05
 0
 2.12028369395825E-05
 0
 0
 0
 0
 0
 3.38196600447772E-05

 0
 0
 0
 0
 6.48182505648112E-04

 0
 0
 0
 0
 6.48182505648112E-04

 0
 0
 0
 0
 6.48182505648112E-04

 0
 0
 0
 0
 6.29394606933674E-04
 0
 0
 0
 1.81802756129783E-05
 0
 2.12028369395825E-05
 0
 0
 0
 0
 0
 3.38196600447772E-05

 0
 0
 0
 0
 6.05909733540627E-04
 0
 0
 0
 1.81802756129783E-05
 0
 2.12028369395825E-05
 0
 0
 0
 0
 0
 3.38196600447772E-05

 0
 0
 0
 0
 6.05909733540627E-04
 0
 0
 0
 1.81802756129783E-05
 0
 2.12028369395825E-05
 0
 0
 0
 0
 0
 3.38196600447772E-05

 0
 0
 0
 0
 2.34848733930475E-05

 0
 0
 0
 0
 2.34848733930475E-05

 0
 0
 0
 0
 5.16667214647046E-05

 0
 0
 0
 0
 5.16667214647046E-05

 0
 0
 0
 0
 5.16667214647046E-05

 0
 0
 0
 0
 5.63636961433141E-05

 0
 0
 0
 0
 5.63636961433141E-05

 0
 0
 0
 0
 5.63636961433141E-05

 0
 0
 0
 0
 5.63636961433141E-05

 0
 0
 2.60796057483049E-03
 6.1166889488907E-04
 2.77591203505822E-03
 8.7377192421627E-04
 2.99563472299534E-03
 1.39134951627415E-05
 3.72089640878956E-03
 0
 3.11681703011863E-03
 2.2012514522145E-04
 0
 1.85967799675486E-05
 0
 1.39423076923077E-03
 2.38090406715232E-03
 6.82022788654778E-05

 0
 0
 0
 0
 0
 0
 0
 0
 8.72653229422958E-04
 0
 2.76697022061552E-03

 0
 0
 0
 0
 0
 0
 0
 0
 8.72653229422958E-04
 0
 2.74576738367594E-03

 0
 0
 0
 0
 0
 0
 0
 0
 8.72653229422958E-04
 0
 2.74576738367594E-03

 0
 0
 0
 0
 0
 0
 0
 0
 8.72653229422958E-04
 0
 2.74576738367594E-03

 0
 0
 0
 0
 0
 0
 0
 0
 0
 0
 2.12028369395825E-05

 0
 0
 0
 0
 0
 0
 0
 0
 0
 0
 2.12028369395825E-05

 0
 0
 0
 0
 0
 0
 0
 0
 0
 0
 2.12028369395825E-05

 0
 0
 2.60796057483049E-03
 6.1166889488907E-04
 2.70545741487908E-03
 8.7377192421627E-04
 2.92239182023262E-03
 0
 2.81188262814065E-03
 0
 2.0672766016093E-04
 1.34520922079775E-04
 0
 1.85967799675486E-05
 0
 1.39423076923077E-03
 2.38090406715232E-03
 6.82022788654778E-05

 0
 0
 2.06838252486556E-03
 4.28168226422349E-04
 1.34803173276093E-03
 8.31148903522793E-04
 1.1865350247561E-03
 0
 2.00589040929861E-03
 0
 6.36085108187476E-05
 0
 0
 0
 0
 1.05769230769231E-03
 1.82626164241797E-03

 0
 0
 2.06838252486556E-03
 4.28168226422349E-04
 1.34803173276093E-03
 8.31148903522793E-04
 1.1865350247561E-03
 0
 2.00589040929861E-03
 0
 5.30070923489563E-05
 0
 0
 0
 0
 1.05769230769231E-03
 1.82626164241797E-03

 0
 0
 2.06838252486556E-03
 4.28168226422349E-04
 1.34803173276093E-03
 8.31148903522793E-04
 1.1865350247561E-03
 0
 2.00589040929861E-03
 0
 5.30070923489563E-05
 0
 0
 0
 0
 1.05769230769231E-03
 1.82626164241797E-03

 0
 0
 0
 0
 0
 0
 0
 0
 0
 0
 1.06014184697913E-05

 0
 0
 0
 0
 0
 0
 0
 0
 0
 0
 1.06014184697913E-05

 0
 0
 5.39578049964927E-04
 1.83500668466721E-04
 1.35742568211815E-03
 0
 1.73585679547652E-03
 0
 8.05992218842038E-04
 0
 1.32517730872391E-04
 1.34520922079775E-04
 0
 1.85967799675486E-05
 0
 3.36538461538462E-04
 5.00530968662703E-04
 6.82022788654778E-05

 0
 0
 5.39578049964927E-04
 1.83500668466721E-04
 1.35742568211815E-03
 0
 1.73585679547652E-03
 0
 8.05992218842038E-04
 0
 1.32517730872391E-04
 1.34520922079775E-04
 0
 1.85967799675486E-05
 0
 3.36538461538462E-04
 5.00530968662703E-04
 6.82022788654778E-05

 0
 0
 5.39578049964927E-04
 1.83500668466721E-04
 1.35742568211815E-03
 0
 1.73585679547652E-03
 0
 8.05992218842038E-04
 0
 1.32517730872391E-04
 1.34520922079775E-04
 0
 1.85967799675486E-05
 0
 3.36538461538462E-04
 5.00530968662703E-04
 6.82022788654778E-05

 0
 0
 0
 0
 0
 4.26230206934765E-05
 0
 0
 0
 0
 0
 0
 0
 0
 0
 0
 5.41114560716436E-05

 0
 0
 0
 0
 0
 4.26230206934765E-05
 0
 0
 0
 0
 0
 0
 0
 0
 0
 0
 5.41114560716436E-05

 0
 0
 0
 0
 0
 4.26230206934765E-05
 0
 0
 0
 0
 0
 0
 0
 0
 0
 0
 5.41114560716436E-05

 0
 0
 0
 0
 0
 0
 0
 0
 0
 0
 1.06014184697913E-05

 0
 0
 0
 0
 0
 0
 0
 0
 0
 0
 1.06014184697913E-05

 0
 0
 0
 0
 0
 0
 0
 0
 0
 0
 1.06014184697913E-05

 0
 0
 0
 0
 4.22727721074856E-05
 0
 0
 0
 1.81802756129783E-05
 0
 9.54127662281213E-05

 0
 0
 0
 0
 4.22727721074856E-05
 0
 0
 0
 1.81802756129783E-05
 0
 9.54127662281213E-05

 0
 0
 0
 0
 4.22727721074856E-05
 0
 0
 0
 1.81802756129783E-05
 0
 9.54127662281213E-05

 0
 0
 0
 0
 4.22727721074856E-05
 0
 0
 0
 1.81802756129783E-05
 0
 9.54127662281213E-05

 0
 0
 0
 0
 1.40909240358285E-05
 0
 7.32429027627223E-05
 0
 1.81802756129783E-05

 0
 0
 0
 0
 1.40909240358285E-05
 0
 7.32429027627223E-05
 0
 1.81802756129783E-05

 0
 0
 0
 0
 1.40909240358285E-05
 0
 7.32429027627223E-05
 0
 1.81802756129783E-05

 0
 0
 0
 0
 1.40909240358285E-05
 0
 7.32429027627223E-05
 0
 1.81802756129783E-05

 0
 0
 0
 0
 0
 0
 0
 1.39134951627415E-05
 0
 0
 0
 5.706948209445E-05

 0
 0
 0
 0
 0
 0
 0
 1.39134951627415E-05
 0
 0
 0
 5.706948209445E-05

 0
 0
 0
 0
 0
 0
 0
 1.39134951627415E-05
 0
 0
 0
 5.706948209445E-05

 0
 0
 0
 0
 0
 0
 0
 1.39134951627415E-05
 0
 0
 0
 5.706948209445E-05

 0
 0
 0
 0
 1.40909240358285E-05
 0
 0
 0
 0
 0
 4.77063831140607E-05
 2.8534741047225E-05

 0
 0
 0
 0
 1.40909240358285E-05
 0
 0
 0
 0
 0
 4.77063831140607E-05
 2.8534741047225E-05

 0
 0
 0
 0
 1.40909240358285E-05
 0
 0
 0
 0
 0
 4.77063831140607E-05
 2.8534741047225E-05

 0
 0
 0
 0
 1.40909240358285E-05
 0
 0
 0
 0
 0
 4.77063831140607E-05
 2.8534741047225E-05

 0
 0
 0
 0
 0
 0
 0
 0
 2.08467160362151E-03
 0
 2.86238298684364E-04

 0
 0
 0
 0
 0
 0
 0
 0
 2.06649132800853E-03
 0
 2.86238298684364E-04

 0
 0
 0
 0
 0
 0
 0
 0
 2.06649132800853E-03
 0
 2.86238298684364E-04

 0
 0
 0
 0
 0
 0
 0
 0
 2.06649132800853E-03
 0
 2.86238298684364E-04

 0
 0
 0
 0
 0
 0
 0
 0
 2.06649132800853E-03
 0
 2.12028369395825E-04

 0
 0
 0
 0
 0
 0
 0
 0
 0
 0
 7.42099292885388E-05

 0
 0
 0
 0
 0
 0
 0
 0
 1.81802756129783E-05

 0
 0
 0
 0
 0
 0
 0
 0
 1.81802756129783E-05

 0
 0
 0
 0
 0
 0
 0
 0
 1.81802756129783E-05

 0
 0
 0
 0
 0
 0
 0
 0
 1.81802756129783E-05

 0
 0
 0
 0
 9.39394935721902E-05
 0
 2.05080127735622E-04
 0
 3.45425236646588E-04
 0
 1.11314893932808E-04
 8.15278315635E-06
 1.42713410065577E-05
 0
 1.30624187680833E-05
 1.66666666666667E-03
 4.53183444600015E-04

 0
 0
 0
 0
 9.39394935721902E-05
 0
 2.05080127735622E-04
 0
 3.45425236646588E-04
 0
 1.11314893932808E-04
 8.15278315635E-06
 1.42713410065577E-05
 0
 1.30624187680833E-05
 1.66666666666667E-03
 4.53183444600015E-04

 0
 0
 0
 0
 9.39394935721902E-05
 0
 2.05080127735622E-04
 0
 3.45425236646588E-04
 0
 1.11314893932808E-04
 8.15278315635E-06
 1.42713410065577E-05
 0
 1.30624187680833E-05
 1.66666666666667E-03
 4.53183444600015E-04

 0
 0
 0
 0
 9.39394935721902E-05
 0
 2.05080127735622E-04
 0
 3.45425236646588E-04
 0
 1.11314893932808E-04
 8.15278315635E-06
 1.42713410065577E-05
 0
 1.30624187680833E-05
 1.66666666666667E-03
 4.53183444600015E-04

 0
 0
 0
 0
 9.39394935721902E-05
 0
 2.05080127735622E-04
 0
 3.45425236646588E-04
 0
 1.11314893932808E-04
 8.15278315635E-06
 1.42713410065577E-05
 0
 1.30624187680833E-05
 1.66666666666667E-03
 4.53183444600015E-04

 0
 0
 0
 0
 1.16954669497377E-03
 0
 1.68458676354261E-04

 0
 0
 0
 0
 1.16954669497377E-03
 0
 1.68458676354261E-04

 0
 0
 0
 0
 1.16954669497377E-03
 0
 1.68458676354261E-04

 0
 0
 0
 0
 1.16954669497377E-03
 0
 1.68458676354261E-04

 0
 0
 0
 0
 1.16954669497377E-03
 0
 1.68458676354261E-04

 0
 0
 1.07915609992985E-04
 0
 1.50303189715504E-04
 0
 1.318372249729E-04
 0
 3.69665604130559E-04
 0
 9.64729080751005E-04
 0
 0
 0
 0
 6.41025641025641E-05
 4.73475240626881E-05

 0
 0
 1.07915609992985E-04
 0
 1.50303189715504E-04
 0
 1.318372249729E-04
 0
 3.69665604130559E-04
 0
 9.64729080751005E-04
 0
 0
 0
 0
 6.41025641025641E-05
 4.73475240626881E-05

 0
 0
 1.07915609992985E-04
 0
 1.50303189715504E-04
 0
 1.318372249729E-04
 0
 3.69665604130559E-04
 0
 9.64729080751005E-04
 0
 0
 0
 0
 6.41025641025641E-05
 4.73475240626881E-05

 0
 0
 1.07915609992985E-04
 0
 1.50303189715504E-04
 0
 1.318372249729E-04
 0
 3.69665604130559E-04
 0
 9.64729080751005E-04
 0
 0
 0
 0
 6.41025641025641E-05
 4.73475240626881E-05

 0
 0
 1.07915609992985E-04
 0
 1.50303189715504E-04
 0
 1.318372249729E-04
 0
 3.69665604130559E-04
 0
 9.64729080751005E-04
 0
 0
 0
 0
 6.41025641025641E-05
 4.73475240626881E-05

 0
 0
 0
 0
 1.40909240358285E-05
 1.49180572427168E-04
 0
 0
 8.05992218842038E-04
 0
 1.06014184697913E-05
 0
 0
 0
 0
 9.61538461538462E-05
 1.08222912143287E-04

 0
 0
 0
 0
 1.40909240358285E-05
 1.49180572427168E-04
 0
 0
 8.05992218842038E-04
 0
 1.06014184697913E-05
 0
 0
 0
 0
 9.61538461538462E-05
 1.08222912143287E-04

 0
 0
 0
 0
 1.40909240358285E-05
 1.49180572427168E-04
 0
 0
 8.05992218842038E-04
 0
 1.06014184697913E-05
 0
 0
 0
 0
 9.61538461538462E-05
 1.08222912143287E-04

 0
 0
 0
 0
 1.40909240358285E-05
 1.49180572427168E-04
 0
 0
 8.05992218842038E-04
 0
 1.06014184697913E-05
 0
 0
 0
 0
 9.61538461538462E-05
 1.08222912143287E-04

 0
 0
 0
 0
 1.40909240358285E-05
 1.49180572427168E-04
 0
 0
 8.05992218842038E-04
 0
 1.06014184697913E-05
 0
 0
 0
 0
 9.61538461538462E-05
 1.08222912143287E-04

 0
 0
 3.9569056997428E-04
 0
 4.18030746396246E-04
 2.34426613814121E-04
 1.83107256906806E-04
 0
 5.029876252924E-04
 0
 2.59734752509886E-04
 0
 0
 0
 0
 9.61538461538462E-05
 1.62334368214931E-04
 4.09213673192867E-05

 0
 0
 3.9569056997428E-04
 0
 3.52273100895713E-04
 2.34426613814121E-04
 1.61134386077989E-04
 0
 4.90867441550414E-04
 0
 4.77063831140607E-05
 0
 0
 0
 0
 9.61538461538462E-05
 1.62334368214931E-04
 4.09213673192867E-05

 0
 0
 3.9569056997428E-04
 0
 3.52273100895713E-04
 2.34426613814121E-04
 1.61134386077989E-04
 0
 4.90867441550414E-04
 0
 4.77063831140607E-05
 0
 0
 0
 0
 9.61538461538462E-05
 1.62334368214931E-04
 4.09213673192867E-05

 0
 0
 3.9569056997428E-04
 0
 3.52273100895713E-04
 2.34426613814121E-04
 1.61134386077989E-04
 0
 4.90867441550414E-04
 0
 4.77063831140607E-05
 0
 0
 0
 0
 9.61538461538462E-05
 1.62334368214931E-04
 4.09213673192867E-05

 0
 0
 3.9569056997428E-04
 0
 3.52273100895713E-04
 2.34426613814121E-04
 1.61134386077989E-04
 0
 4.90867441550414E-04
 0
 4.77063831140607E-05
 0
 0
 0
 0
 9.61538461538462E-05
 1.62334368214931E-04
 4.09213673192867E-05

 0
 0
 0
 0
 0
 0
 0
 0
 0
 0
 1.59021277046869E-04

 0
 0
 0
 0
 0
 0
 0
 0
 0
 0
 1.59021277046869E-04

 0
 0
 0
 0
 0
 0
 0
 0
 0
 0
 1.59021277046869E-04

 0
 0
 0
 0
 0
 0
 0
 0
 0
 0
 1.59021277046869E-04

 0
 0
 0
 0
 6.57576455005331E-05

 0
 0
 0
 0
 6.57576455005331E-05

 0
 0
 0
 0
 6.57576455005331E-05

 0
 0
 0
 0
 6.57576455005331E-05

 0
 0
 0
 0
 0
 0
 0
 0
 1.21201837419855E-05
 0
 5.30070923489563E-05

 0
 0
 0
 0
 0
 0
 0
 0
 0
 0
 5.30070923489563E-05

 0
 0
 0
 0
 0
 0
 0
 0
 0
 0
 5.30070923489563E-05

 0
 0
 0
 0
 0
 0
 0
 0
 0
 0
 5.30070923489563E-05

 0
 0
 0
 0
 0
 0
 0
 0
 1.21201837419855E-05

 0
 0
 0
 0
 0
 0
 0
 0
 1.21201837419855E-05

 0
 0
 0
 0
 0
 0
 0
 0
 1.21201837419855E-05

 0
 0
 0
 0
 0
 0
 2.19728708288167E-05

 0
 0
 0
 0
 0
 0
 2.19728708288167E-05

 0
 0
 0
 0
 0
 0
 2.19728708288167E-05

 0
 0
 0
 0
 0
 0
 2.19728708288167E-05

 0
 0
 0
 0
 0
 0
 0
 0
 4.54506890324457E-04
 0
 2.0672766016093E-04

 0
 0
 0
 0
 0
 0
 0
 0
 4.54506890324457E-04
 0
 2.0672766016093E-04

 0
 0
 0
 0
 0
 0
 0
 0
 4.54506890324457E-04
 0
 2.0672766016093E-04

 0
 0
 0
 0
 0
 0
 0
 0
 4.54506890324457E-04
 0
 2.0672766016093E-04

 0
 0
 0
 0
 0
 0
 0
 0
 4.54506890324457E-04
 0
 2.0672766016093E-04

 0
 0
 0
 0
 0
 0
 0
 0
 0
 0
 3.97553192617172E-04

 0
 0
 0
 0
 0
 0
 0
 0
 0
 0
 3.97553192617172E-04

 0
 0
 0
 0
 0
 0
 0
 0
 0
 0
 3.97553192617172E-04

 0
 0
 0
 0
 0
 0
 0
 0
 0
 0
 3.97553192617172E-04

 0
 0
 0
 0
 0
 0
 0
 0
 0
 0
 3.97553192617172E-04

 0
 0
 5.39578049964927E-05
 1.74762541396877E-05
 3.42879151538494E-04
 0
 7.32429027627223E-05
 1.85513268836554E-05
 9.69614699358842E-05
 0
 1.64321986281764E-04
 0
 0
 1.39475849756615E-05
 1.1756176891275E-04
 0
 0
 3.04636845599134E-04

 0
 0
 5.39578049964927E-05
 1.74762541396877E-05
 3.42879151538494E-04
 0
 7.32429027627223E-05
 1.85513268836554E-05
 9.69614699358842E-05
 0
 1.64321986281764E-04
 0
 0
 1.39475849756615E-05
 1.1756176891275E-04
 0
 0
 3.04636845599134E-04

 0
 0
 5.39578049964927E-05
 1.74762541396877E-05
 3.42879151538494E-04
 0
 7.32429027627223E-05
 1.85513268836554E-05
 9.69614699358842E-05
 0
 1.64321986281764E-04
 0
 0
 1.39475849756615E-05
 1.1756176891275E-04
 0
 0
 3.04636845599134E-04

 0
 0
 5.39578049964927E-05
 1.74762541396877E-05
 3.42879151538494E-04
 0
 7.32429027627223E-05
 1.85513268836554E-05
 9.69614699358842E-05
 0
 1.64321986281764E-04
 0
 0
 1.39475849756615E-05
 1.1756176891275E-04
 0
 0
 3.04636845599134E-04

 0
 0
 5.39578049964927E-05
 1.74762541396877E-05
 3.42879151538494E-04
 0
 7.32429027627223E-05
 1.85513268836554E-05
 9.69614699358842E-05
 0
 1.64321986281764E-04
 0
 0
 1.39475849756615E-05
 1.1756176891275E-04
 0
 0
 3.04636845599134E-04

 0
 0
 2.51803089983633E-04
 2.62143812095315E-05
 0
 3.40984165547812E-04
 1.61134386077989E-04
 0
 5.45408268389349E-05
 0
 2.65035461744781E-05
 0
 0
 0
 0
 0
 2.77321212367173E-04

 0
 0
 2.51803089983633E-04
 2.62143812095315E-05
 0
 3.40984165547812E-04
 1.61134386077989E-04
 0
 5.45408268389349E-05
 0
 2.65035461744781E-05
 0
 0
 0
 0
 0
 2.77321212367173E-04

 0
 0
 2.51803089983633E-04
 2.62143812095315E-05
 0
 3.40984165547812E-04
 1.61134386077989E-04
 0
 5.45408268389349E-05
 0
 2.65035461744781E-05
 0
 0
 0
 0
 0
 2.77321212367173E-04

 0
 0
 2.51803089983633E-04
 2.62143812095315E-05
 0
 3.40984165547812E-04
 1.61134386077989E-04
 0
 5.45408268389349E-05
 0
 2.65035461744781E-05
 0
 0
 0
 0
 0
 2.77321212367173E-04

 0
 0
 2.51803089983633E-04
 2.62143812095315E-05
 0
 3.40984165547812E-04
 1.61134386077989E-04
 0
 5.45408268389349E-05
 0
 2.65035461744781E-05
 0
 0
 0
 0
 0
 2.77321212367173E-04

 0
 0
 0
 1.66024414327033E-04
 6.10606708219236E-05
 0
 2.92971611050889E-04
 0
 3.03004593549638E-05
 0
 2.65035461744781E-05
 0
 0
 0
 0
 0
 2.29973688304485E-04

 0
 0
 0
 1.66024414327033E-04
 6.10606708219236E-05
 0
 2.92971611050889E-04
 0
 3.03004593549638E-05
 0
 2.65035461744781E-05
 0
 0
 0
 0
 0
 2.29973688304485E-04

 0
 0
 0
 1.66024414327033E-04
 0
 0
 2.92971611050889E-04
 0
 3.03004593549638E-05
 0
 2.65035461744781E-05
 0
 0
 0
 0
 0
 2.29973688304485E-04

 0
 0
 0
 1.66024414327033E-04
 0
 0
 2.92971611050889E-04
 0
 3.03004593549638E-05
 0
 2.65035461744781E-05
 0
 0
 0
 0
 0
 2.29973688304485E-04

 0
 0
 0
 1.66024414327033E-04
 0
 0
 2.92971611050889E-04
 0
 3.03004593549638E-05
 0
 2.65035461744781E-05
 0
 0
 0
 0
 0
 2.29973688304485E-04

 0
 0
 0
 0
 6.10606708219236E-05

 0
 0
 0
 0
 6.10606708219236E-05

 0
 0
 0
 0
 6.10606708219236E-05

 0
 0
 3.59718699976618E-05
 8.73812706984385E-05
 2.8181848071657E-05
 6.39345310402148E-05
 1.83107256906806E-04
 0
 1.81802756129783E-05
 0
 6.36085108187475E-05
 0
 0
 0
 0
 2.24358974358974E-04
 3.65252328483594E-04

 0
 0
 3.59718699976618E-05
 8.73812706984385E-05
 2.8181848071657E-05
 6.39345310402148E-05
 1.83107256906806E-04
 0
 1.81802756129783E-05
 0
 6.36085108187475E-05
 0
 0
 0
 0
 2.24358974358974E-04
 3.65252328483594E-04

 0
 0
 3.59718699976618E-05
 8.73812706984385E-05
 2.8181848071657E-05
 6.39345310402148E-05
 1.83107256906806E-04
 0
 1.81802756129783E-05
 0
 6.36085108187475E-05
 0
 0
 0
 0
 2.24358974358974E-04
 3.65252328483594E-04

 0
 0
 3.59718699976618E-05
 8.73812706984385E-05
 2.8181848071657E-05
 6.39345310402148E-05
 1.83107256906806E-04
 0
 1.81802756129783E-05
 0
 6.36085108187475E-05
 0
 0
 0
 0
 2.24358974358974E-04
 3.65252328483594E-04

 0
 0
 0
 8.73812706984385E-05
 1.40909240358285E-05
 6.39345310402148E-05
 9.5215773591539E-05
 0
 1.81802756129783E-05
 0
 3.71049646442694E-05
 0
 0
 0
 0
 2.24358974358974E-04
 1.69098300223886E-04

 0
 0
 3.59718699976618E-05
 0
 0
 0
 2.19728708288167E-05
 0
 0
 0
 0
 0
 0
 0
 0
 0
 1.14986844152243E-04

 0
 0
 0
 0
 1.40909240358285E-05
 0
 6.59186124864501E-05
 0
 0
 0
 2.65035461744781E-05
 0
 0
 0
 0
 0
 8.11671841074653E-05

 0
 0
 0
 1.74762541396877E-05
 1.17424366965238E-04
 0
 2.92971611050889E-05

 0
 0
 0
 1.74762541396877E-05
 1.17424366965238E-04
 0
 2.92971611050889E-05

 0
 0
 0
 1.74762541396877E-05
 1.17424366965238E-04
 0
 2.92971611050889E-05

 0
 0
 0
 1.74762541396877E-05
 1.17424366965238E-04
 0
 2.92971611050889E-05

 0
 0
 0
 1.74762541396877E-05
 1.17424366965238E-04
 0
 2.92971611050889E-05

 0
 0
 0
 0
 0
 0
 0
 0
 1.21201837419855E-05
 0
 9.54127662281213E-05

 0
 0
 0
 0
 0
 0
 0
 0
 1.21201837419855E-05
 0
 9.54127662281213E-05

 0
 0
 0
 0
 0
 0
 0
 0
 1.21201837419855E-05
 0
 9.54127662281213E-05

 0
 0
 0
 0
 0
 0
 0
 0
 1.21201837419855E-05
 0
 9.54127662281213E-05

 0
 0
 0
 0
 0
 0
 0
 0
 1.21201837419855E-05
 0
 9.54127662281213E-05

 0
 0
 0
 0
 5.63636961433141E-05
 0
 0
 0
 1.81802756129783E-05

 0
 0
 0
 0
 5.63636961433141E-05
 0
 0
 0
 1.81802756129783E-05

 0
 0
 0
 0
 5.63636961433141E-05
 0
 0
 0
 1.81802756129783E-05

 0
 0
 0
 0
 5.63636961433141E-05
 0
 0
 0
 1.81802756129783E-05

 0
 0
 0
 0
 5.63636961433141E-05
 0
 0
 0
 1.81802756129783E-05

 0
 0
 0
 0
 0
 0
 0
 0
 0
 0
 1.59021277046869E-05

 0
 0
 0
 0
 0
 0
 0
 0
 0
 0
 1.59021277046869E-05

 0
 0
 0
 0
 0
 0
 0
 0
 0
 0
 1.59021277046869E-05

 0
 0
 0
 0
 0
 0
 0
 0
 0
 0
 1.59021277046869E-05

 0
 0
 0
 0
 0
 0
 0
 0
 0
 0
 1.59021277046869E-05

 0
 0
 0
 0
 0
 0
 0
 0
 1.21201837419855E-05

 0
 0
 0
 0
 0
 0
 0
 0
 1.21201837419855E-05

 0
 0
 0
 0
 0
 0
 0
 0
 1.21201837419855E-05

 0
 0
 0
 0
 0
 0
 0
 0
 1.21201837419855E-05

 0
 0
 0
 0
 0
 0
 0
 0
 1.21201837419855E-05
